# Development of a Reporting Guideline for Trochim’s Concept Mapping

**DOI:** 10.3390/mps8020024

**Published:** 2025-03-03

**Authors:** Sandesh Pantha, Martin Jones, Richard Gray

**Affiliations:** 1 George Singer Building, School of Nursing and Midwifery, La Trobe University, Bundoora, VIC 3086, Australia; r.gray@latrobe.edu.au; 2 School of Nursing and Midwifery, Edith Cowan University, Bunbury, WA 6230, Australia; martin.jones@ecu.edu.au

**Keywords:** reporting guidelines, guideline development, concept mapping

## Abstract

Reporting guidelines are created with the intention to enhance the quality and transparency of reporting different research methods. Trochim’s concept mapping (often referred to as group concept mapping) is a six-phase, participatory mixed-method approach to understanding complex constructs. Currently, there is no reporting guideline for concept mapping. Developing a reporting guideline typically follows a three-step process: 1. a systematic review to establish the need for a reporting guideline, 2. a Delphi study to identify candidate items, and 3. consolidation process to inform a draft guideline. We have previously reported our step 1, a systematic review of health-related concept mapping studies. In this paper, we report on steps 2 and 3, guideline development. In developing our reporting guideline, we opted to use concept mapping rather than the Delphi method. Stakeholders including researchers and experts in concept mapping were identified from papers included in our systematic review and invited to participate. Thirty-two stakeholders participated in the brainstorming phase of the concept mapping generating 96 discrete statements. The prioritisation and clustering phase involved 24 stakeholders. The final concept map included 11 clusters that represented key concepts for inclusion in the reporting guideline. The clusters were relatively small and positioned in a circle around the edge of the map, suggesting each was of equal importance and conceptually discreet. In phase 3, a guideline was drafted using the findings from both the phase 1 systematic review and phase 2 concept mapping study. The draft was reviewed by eight stakeholders (who had participated in our phase 2 concept mapping study) to check the completeness and clarity of expression of the items included in the guideline. The final reporting guideline (called the ConMapT) has 27-items organised under 14 headings. The guideline will be made freely available via the EQUATOR network. Registration: The study protocol was registered with the Open Science Framework (OSF) before recruiting the first study participant. The EQUATOR network has listed the study as a guideline under development.

## 1. Background

Concept mapping is a participatory mixed-methods research design to elaborate a deep understanding of a particular issue or topic [1]. Two approaches to concept mapping are widely used in research [1,2]. In Trochim’s approach (often described as group concept mapping), ideas and concepts on a particular topic are generated using brainstorming, prioritising, and clustering and presented as a two-dimensional cluster map [3]. An alternative approach to concept mapping has been described by Novak [2]. In Novak’s concept mapping, the relationship between participants’ generated ideas is shown as a hierarchical flow diagram (using arrows and directions) [2]. Novak [4] and Trochim [5] acknowledge that these methodologies are essentially different but mistaken for each other by researchers. In this manuscript, we will use concept mapping to mean ‘Group Concept Mapping (GCM)’, as described by Trochim [5].

There are typically six discrete, connected phases in Trochim’s concept mapping: 1. preparation, 2. idea generation (brainstorming), 3. idea structuring (prioritisation/rating and clustering), 4. concept mapping analysis, 5. interpretation, and 6. utilisation [5]. Stakeholders actively participate in all phases of the research process [6]. Computerised software packages (notably, Ariadne 3.0 and Concept Systems 12) are commonly used to manage concept mapping research. Concept mapping has been extensively used in health research to develop a deep understanding of complex phenomena [7,8]. For example, Kikkert et al. (2006) used concept mapping to understand factors influencing treatment adherence in people with schizophrenia. Eighty-one patients, carers, and health professionals identified ten factors affecting medication adherence, including professional and non-professional support, side effect self-management, and patients’ insight into their illness [9]. Understanding the role of general practitioners working in Australia in the management of age-related hearing loss is another example of health-related concept mapping [10]. General practitioners, allied health workers, and patients participated in the research. A six-cluster concept map including stakeholder partnership and patient empowerment was identified as a successful approach to the management of hearing loss [10].

Over recent years, there has been an increase in concept mapping research. In 2017, *Planning and Evaluation* published a special issue on Trochim concept mapping methodology. Four papers published in that special issue showed an exponential increase in the use of concept mapping research since the beginning of this millennium [7,11,12,13]. Trochim (2017), who developed the concept mapping methodology, noted that the original methodology paper was cited in nearly 500 publications in Scopus between 1989 and 2015 [7]. Trochim (2017) further noted that these concept mapping papers had themselves been cited over 6000 times. There has been considerable interest in concept mapping by mental health researchers in the Netherlands. A paper by Nabitz et al. reported over 90 papers from Dutch authors using the concept mapping approach to evaluate different aspects of mental health care and treatment [13]. More than half of these studies were published between 2000 and 2014 [13]. Finally, in a review of the use of concept mapping by PhD students, Donnelly (2017) reported a fivefold increment in the number of doctoral theses using concept mapping between 2001 and 2014 [12].

Complete, transparent reporting of research enables the reader to make an accurate determination of the quality and significance of the work [14]. There is some evidence to suggest that the quality of reporting of Trochim’s concept mapping is sub-optimal [8,12]. For example, Donnelly (2017) conducted a systematic review of 104 doctoral dissertations that used concept mapping, noting important omissions in the quality of reporting in many included studies. For example, few study authors described the process that was followed when developing the focus prompt for the research and tended not to report the number of participants involved in each phase of the study [12]. A pooled analysis of concept mapping studies using a specific software (Concept Systems) was reported by Scott and Rosas (2012). The authors note that value—an indicator of the validity of a concept—were often not reported in published manuscripts [8]. The authors of both reviews suggest that having a reporting guideline may potentially enhance the quality of reporting for concept mapping studies [8,12].

Typically, reporting guidelines take the form of a checklist of required information to comprehensively describe the conduct, findings, and implications of the research [15]. There is some evidence indicating that reporting guidelines positively impact the quality of research reporting [16]. For example, Tunis et al. (2013) reviewed 130 systematic reviews and reported an improvement in the quality of reporting of systematic reviews following the publication of the PRISMA (Preferred Reporting Items for Systematic Review and Meta-Analysis) guideline [16].

Journal editors mandating authors follow reporting guidelines when submitting work has also seemingly enhanced the quality of research reporting [16,17,18,19]. An umbrella review of 53 systematic reviews evaluating the quality of reporting of clinical trials before and after journal endorsement of the CONSORT (Consolidated Standards of Reporting Trials) guideline was reported by Turner et al. (2012). The CONSORT endorsement was associated with more comprehensive reporting in 69 of 81 (86%) meta-analyses (Turner et al. 2012). Similar enhancements in the quality of reporting have been observed by authors evaluating journal endorsement of guidelines for observational research [17] qualitative studies [20], and systematic reviews [16].

In 2006, a group of methodologists established the EQUATOR (Enhancing the Quality and Transparency of Health Research) network—a publicly accessible repository of reporting guidelines [21]. The EQUATOR repository includes a list of 617 reporting guidelines across different methodologies including observational, qualitative and interventional studies and literature reviews [22]. A standard process for the development of reporting guidelines has been developed and published by the EQUATOR consortium [23,24].

The EQUATOR network does not currently (as of 31 January 2024) list a reporting guideline for concept mapping [22]. We did, however, identify a 10-point checklist on the website of the Group Wisdom software (formally Concept Systems), a package used to generate concept maps. The checklist provides recommendations on the conduct and reporting of the concept mapping research [25]. We concluded that the checklist could not be recommended for use as the EQUATOR network guideline development process was not followed. Further, the checklist only applies to the concept mapping studies using the Concept Systems software package. Further, no detail is provided as to how the checklist was developed.

The authors of two concept mapping studies have attempted to adapt reporting guidelines from other study designs [26,27]. For example, Cardwell et al. (2021) applied the STROBE (Strengthening the Reporting of Observational Studies in Epidemiology) guideline developed for observational studies). The GRAMMS (Good Reporting of A Mixed-Methods Study) checklist developed for mixed-methods research was used by Dopp et al. (2020). Neither STROBE nor GRAMMS addresses the specific methodological requirements of concept mapping such as idea generation (brainstorming) and structuring statements (sorting and rating). There is justification for developing a specific reporting guideline for Trochim’s concept mapping following the EQUATOR methodology. We followed the three-staged approach to guideline development described by the EQUATOR network consortium [23,24]: stage 1, a systematic review of concept mapping studies to examine the quality of reporting and identify candidate items for inclusion in the guideline; stage 2, a concept mapping study to identify candidate items to include in the reporting guideline; and stage 3, a consolidation and stakeholder review. Additionally, we conducted a small pilot test of a draft reporting guideline to establish face validity.

We (prospectively) registered our study protocol with the Open Science Framework (OSF) https://osf.io/h54k6/ (accessed on 17 July 2021). This study was also registered with the EQUATOR network as a guideline under development (URL: https://www.equator-network.org, accessed on 18 February 2025). We have previously reported the systematic review (stage 1) [28]. In the review, we extracted data on the quality of reporting of 75 concept mapping studies, published between 2019 and 2021. The included studies consistently reported the rationale for the study, the focus prompt, a description of the stakeholder groups, procedures for brainstorming and structuring statements, and the process of involving stakeholders in data interpretation [28]. We also identified important consistent omissions in the reporting of concept mapping studies. For example, authors omitted the process they followed in developing the focus prompt, did not give a rationale for deciding on the stakeholder groups, or did not report the number of cluster solutions considered for interpretation [28]. Almost all studies (3/75, 4%) indicated a publicly available study protocol.

This paper reports stages 2 (concept mapping) and 3 (consolidation) of the guideline development process. Our protocol describing and discussing the methodology for guideline development has been previously reported [29]. In our protocol paper, we gave a detailed and considered discussion of the methodological decisions we made and direct interested readers to that paper. Key facts about our concept mapping methodology are described below for clarity and brevity.

## 2. Concept Mapping Study

We used concept mapping to generate candidate items for inclusion in the reporting guideline. The EQUATOR network suggests using the Delphi methodology to identify potential items for inclusion [24]. Concept mapping is often used as an alternative to Delphi and may therefore be well suited to support the development of reporting guidelines [26]. It also seemed to make sense to use concept mapping to inform the development of a reporting guideline about concept mapping. We strictly followed the six phases of concept mapping proposed by Trochim: 1. preparation, 2. idea generation, 3. idea structuring, 4. data analysis, 5. interpretation, and 6. utilisation [1]. We used the ARIADNE concept mapping software package for phases 3 through 5 [30].

### 2.1. Phase 1: Preparation

The preparation phase involved determining the focus question, stakeholder identification, and participant recruitment.

#### 2.1.1. Focus Prompt

The focus prompt for the study was “What are the items that need to be included in the report of a concept mapping study?” The focus prompt was drafted by the research team and reviewed with two additional researchers.

#### 2.1.2. Identification of Stakeholder Groups

We identified six stakeholder groups with relevant experience: 1. concept mapping researchers, 2. concept mapping experts, 3. statisticians involved in concept mapping, 4. people who have participated in concept mapping research, 5. peer reviewers of concept mapping research, and 6. journal editors who had handled concept mapping papers [24].

#### 2.1.3. Stakeholder Recruitment

We adopted a systematic approach to identify and recruit each of the six stakeholder groups [29]. In summary, potential stakeholders (with the exception of people who had participated in concept mapping) were identified from our systematic review [28]. To identify concept mapping researchers and concept mapping experts, we extracted details of the corresponding authors from the 258 concept mapping studies included in our review [28]. We then checked if the corresponding author of the included paper had been involved in other concept mapping studies. We a priori defined concept mapping experts as researchers who had been an author of at least five concept mapping papers.

Peer reviewers of a concept mapping study were identified by checking the peer reviewer report or similar section of manuscripts included in our review. The name, email, and institutional affiliation of potential participants were extracted from the paper. Where necessary, we also checked relevant institutional researcher profiles to find contact details.

To identify the handling editors of papers included in our systematic review, we reviewed the journal website and extracted contact information. We reviewed the author contribution section of the included manuscripts to identify statisticians. Names and contact information were extracted from papers where we could clearly determine whether a researcher identified themselves and a statistician.

Potential participants across these five stakeholder groups were contacted by email explaining that we were developing a reporting guideline for concept mapping and enquiring if they were interested in participating. We attached a copy of the participant information sheet and consent form to the email (Supplementary Document S1 is a copy of the PICF form used in this study). Participants were asked to contact the researcher if additional information was required. They were asked to send a scanned copy of the consent form if they wished to take part. A follow-up email was sent to potential participants after two weeks if we did not receive a response from them.

Recruitment of people who had participated in concept mapping research was carried out via social media (the post we used to recruit can be accessed as Supplementary Document S2). People interested in participating in the research could directly contact a study researcher (SP) to express their interest in participating. They were then sent a copy of the information and consent form and invited to contact us if they wished to discuss the research. If they wanted to participate, they were asked to email us the signed consent form.

#### 2.1.4. Ethical Considerations

Several ethical issues need consideration in our study. We extracted contact information from publicly available sources (published manuscripts and institutional websites) and directly contacted researchers, statisticians, editors, and peer reviewers. It is possible that receiving these emails may have caused some worry or anxiety or confused potential participants. The participants were not paid for taking part in the research. On the other hand, we considered that because participants were employed in public sector organisations (universities, hospitals), they might be willing to contribute to our study because they would perceive that there is the potential to enhance the quality of reporting of concept mapping research. The Human Research Ethics Committee (HREC) of La Trobe University reviewed and approved this study (ethics approval number—HEC21286).

### 2.2. Phase 2: Idea Generation

#### 2.2.1. Brainstorming

Individual brainstorming interviews were conducted via video conferencing. The audio part of the interviews was retained. Participants were asked to respond to the focus prompt (‘What are the items that need to be included in the report of concept mapping research?’). Participants were reminded that the focus of the research was on Trochim’s concept mapping. For further elaboration, open-ended questions were asked to elicit additional details from participants. For example, ‘What information needs to be included to report the brainstorming phase?’ and ‘How would you conduct the process of reducing the number of statements?’

#### 2.2.2. Statement Reduction (Idea Synthesis)

We listened to audio interviews of the brainstorming sessions between two and four times to identify and extract verbatim statements. A statement was considered a phrase or sentence that was singular, specific, and not overlapping [5]. Any similar statements were combined to produce a final list of no more than 98 statements (the maximum number of statements “Ariadne” can handle) [30].

### 2.3. Phase 3, Idea Structuring

Participants completed two structuring tasks (prioritising and clustering) using the web link of the Ariadne software package. For the sorting task (often described by researchers as clustering or grouping), participants were asked to organise statements that seemed to belong together into clusters. Participants were asked to propose labels for each cluster (this was not a mandatory requirement). The rating (sometimes referred to as prioritisation or ranking) task required participants to rank statements on their perceived importance on a five-point scale ranging from “not important” to “extremely important”. Participants were asked to place an equal number of statements on each point on the scale (Supplementary Document S3 is a copy of the instructions sent to the participants explaining how to complete the structuring tasks). Participants were sent two reminder emails (two and three weeks after the first email was sent) if they had not completed the structuring tasks.

### 2.4. Phase 4, Data Analysis

Data from the participants who completed either of the structuring tasks were included in the analysis. The analysis of sorting data involved three steps: group similarity matrix (GSM), principal component analysis (PCA) and hierarchical cluster analysis (HCA) [30]. Ariadne generates a group similarity matrix to determine how frequently pairs of statements were clustered together by participants, which is then used to undertake a principal component analysis to transform and locate individual statements onto a two-dimensional “point map”. Each statement is represented on the point map as a dot. Statements that are frequently grouped appear closer on the map. Ariadne then undertakes a hierarchical cluster analysis to produce 17 candidate concept maps with between 2 and 18 discreet clusters [30].

Rating data were used to calculate the mean (95% confidence interval) importance rating for individual statements and clusters.

### 2.5. Phase 5, Data Interpretation

The final cluster solution was determined following a two-step process. First, the research team reviewed all 17 candidate concept maps to exclude cluster solutions that did not seem to represent the data; for example, where a cluster seemed to capture multiple concepts. Next, we invited 24 participants to take part in a data interpretation workshop to review the shortlisted candidate cluster solutions. The workshop aimed to agree on a final concept map that best represented the data.

### 2.6. Phase 6, Utilisation

The results of this study were used to inform the development of a candidate reporting guideline for Trochim concept mapping research (along with the results of the systematic review [28]).

### 2.7. Results of the Concept Mapping Study

#### 2.7.1. Participants

From our systematic review, we identified 258 concept mapping studies (a complete list of studies included in the systematic review is available as Supplementary Document S4). We were able to extract author contact information from 240 studies (204 discreet authors), of which 14 met our criteria for methodological experts. We were also able to identify three statisticians from the author list of included papers. Peer-review reports were available for 29 studies from which we identified 63 peer-reviewers. Finally, we were able to identify 10 handling editors.

**Figure 1:**
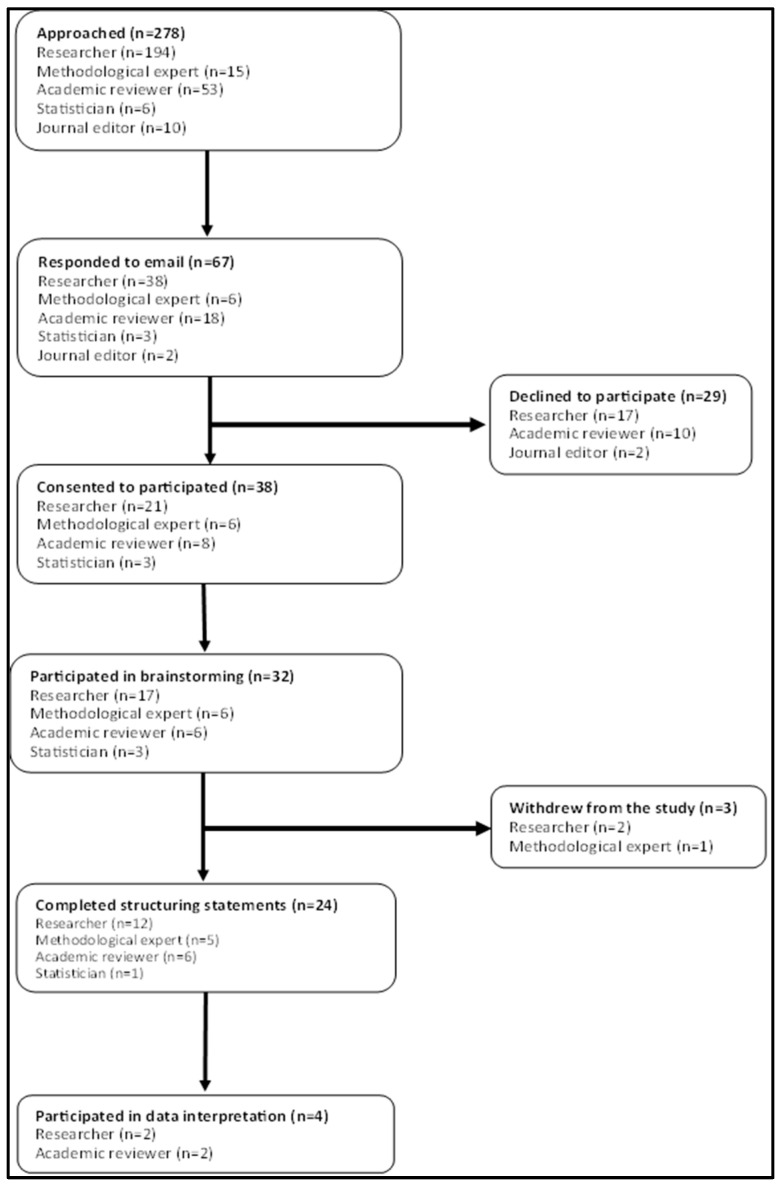
Flow of participants through the different phases of concept mapping.

Figure 1 shows the flow of participants through the study. Six concept mapping experts, seventeen researchers, six peer reviewers, and three statisticians participated in the research. No journal editors and people who had previously been involved in concept mapping responded to our invitations to participate. Of the 278 potential stakeholders we contacted, 67 responded to our email and 38 consented to participate. Thirty-two completed the brainstorming task. Three quarters of the stakeholder that completed the brainstorming task also participated in the prioritising and clustering task (structuring phase).

The demographic characteristics of the study participants at different phases of the study are shown in Table 1. Participants were working across three geographical regions, North America, Europe, and Australasia. They mostly identified as female, were in their late forties (age), and held a doctoral-level qualification. There was no apparent systematic difference in the demographic profile of participants in the brainstorming, structuring, and interpretation phases of concept mapping.

#### 2.7.2. Idea Generation (Brainstorming and Statement Reduction)

The brainstorming interviews lasted for an average of 23 (SD ± 7.7) minutes. In total, 895 discreet statements were generated (Supplementary Document S5). From the full list of statements, duplicates were combined, resulting in 115 statements. The research team then reviewed this list to identify any additional duplicate statements to produce a final list of no more than 98 statements, as this is the maximum that can be handled by the concept mapping computer software we were using. A final list of the 96 statements generated from the brainstorming phase is available as Supplementary Document S6.

#### 2.7.3. Structuring (Sorting and Rating) of Statements

Stakeholders sorted statements into between 4 and 10 clusters (mean 7, SD ± 2).

Scores from the prioritisation task ranged from 1.80 to 4.74 (on a one [least important]-to-five-point [most important] scale). The highest ranked statements, and priorities to include in our reporting guideline were the following: describe the final product (clusters and axis) of the concept mapping research (4.74), the initial question or focus prompt used in the study is clearly (explicitly) defined (4.68), and present the final number of statements included in the card sorting (clustering and ranking) (4.40). The three statements ranked least important were the following: a timeframe of how long to complete the individual stages (1.88), talk about the training (of the researchers) on group concept mapping (2.0), and information on whether any [brainstorming] sessions were recorded (2.08). A table showing the ranking of individual statements can be accessed as Supplementary Document S6.

#### 2.7.4. Data Analysis

Ariadne uses a principal component analysis (PCA) to plot individual statements onto a two-dimensional map, known as a point map. The point map generated from this study is available as Supplementary Document S7. The hierarchical cluster analysis (HCA) in Ariadne was then used to generate a series of candidate concept maps with between 2 and 18 clusters. Supplementary Document S8 is a list of all 17 candidate concept maps.

#### 2.7.5. Interpretation

As per the study protocol, we undertook a preliminary review of candidate concept maps to determine a more manageable number of maps to review by stakeholders. Maps with between 8 and 12 clusters were retained for review by stakeholders at the data interpretation workshop. Concept maps with fewer than eight clusters were rejected as the clusters did not seem to capture a discreet concept. Maps with 13 or more clusters were rejected as they contained clusters that addressed related concepts.

Four research participants attended the data interpretation workshop. During the discussion, participants strongly asserted the need to rerun the analysis, removing one outlying statement from the dataset (‘Provide information on the use of concept mapping software at different phases of the study’) as it formed an individual cluster from concept map (cluster solution) four onwards. We removed this statement and repeated our analysis. Supplementary Document S9 shows the 17 candidate concept maps produced after the removal of this statement. We then reviewed all candidate concept maps, determining that the 11-cluster solution best captured the data.

Description of the Final Concept Map

**Figure 2:**
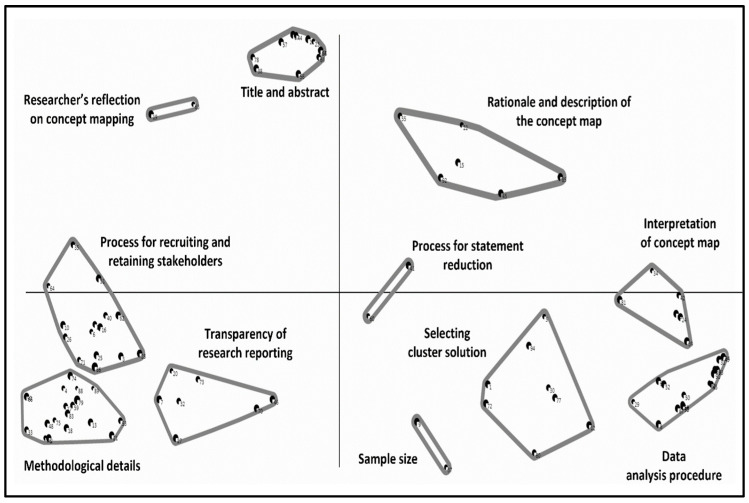
Final concept map with 11 clusters.

Figure 2 shows the final 11-cluster concept map. We labelled the clusters based on suggestions from the stakeholders and our review of statements included in each cluster. Supplementary Document S6 provides information on the statements contained in each cluster of the 11-cluster solution.

There were several notable features of the final concept map. Often, the clusters formed a circle around the middle of the map, suggesting that each of the clusters is of broadly equal importance. Three clusters (interpretation of concept map, process for statement reduction, and process for recruiting and retaining stakeholders) intercepted the *x*-axis, suggesting that they may be thematically related to nearby clusters.

Six clusters (rationale and description of the concept map, selecting cluster solution, data analysis procedures, methodological details, transparency of research reporting, and process for recruiting and retaining stakeholders) were larger, suggesting a lack of clear conceptual focus.

Three clusters (sample size, process for statement reduction, and researcher’s reflection on concept mapping) only contained two statements, potentially reflecting that these clusters represent specific constructs.

## 3. Development of the Draft Reporting Guideline

The final stage of the guideline development included combining the findings of the systematic review [28] and concept mapping. In total, we generated 167 candidate items to include in the reporting guideline, 71 from the systematic review and 96 from concept mapping. Supplementary Document S10 is a list of 167 items used for drafting the reporting guideline. Next, we coded each candidate item against the phase of concept mapping it related to (for example, ‘the number of statements used for structuring phase was reported’ was coded as a part of the structuring phase). We then combined similar candidate items. This resulted in a list of 60 items arranged under 14 headings and 28 sub-headings. The draft checklist (Supplementary Document S11) was then sent to stakeholders (who participated in the structuring phase of the concept mapping, inviting them to give comments on the draft guideline. Eight participants returned feedback. The stakeholders suggested that some items needed to be described more clearly.

### Reporting Guideline

The final version of the reporting guideline comprised a 27-item checklist (the ConMapT) organised under 14 headings: title (item 1); abstract (item 2); background (items 3–5); phase 1, preparation (items 6–10); phase 2, generating the ideas (items 11–13); phase 3, structuring the statements (item 14–16); phase 4, concept mapping analysis (item 17–18); phase 5, interpreting the map (item 19–21); phase 6, utilisation (item 22); discussion (item 23); study limitations (item 24); ethics (item 25); conclusion (item 26); and registration and protocol (item 27). The reporting guideline checklist items is provided as Supplementary Document S12.

The concept map informed how we structured the checklist items. For example, one of the clusters included ‘title and abstract’. For clarity and consistency with existing reporting guidelines (for example, PRISMA and STROBE), ‘title and abstract’ were organised as 1. title and 2. abstract. In addition, two clusters, ‘Details of concept mapping methodology’ and ‘Description of data analysis procedures and reporting’ had 18 and 17 statements, respectively. To add clarity to the checklist items, we arranged these clusters to refine the checklist based on the six phases of the concept mapping process. The draft guideline was pilot-tested against ten concept mapping studies included in our systematic review (phase 1) that we selected at random. Supplementary Document S13 shows the results of the pilot study against each reporting guideline checklist item. No specific issues were identified during the pilot testing. We made one post hoc amendment to the study protocol. Rather than undertaking a second data interpretation workshop, we solicited feedback from stakeholders via email correspondence. We did this because of logistical reasons; it was problematic organising an online workshop across multiple time zones.

## 4. Discussion

The aim of this study was to develop a reporting guideline for concept mapping research following the process described by the EQUATOR network [24]. To identify candidate items for inclusion in the reporting guideline, we conducted a systematic review of the quality of reporting concept mapping studies and completed our concept mapping research. The process of developing the guideline was participatory and involved a range of key stakeholders including concept mapping researchers and concept mapping methodological experts. We undertook the selection and recruitment of stakeholders following a systematic and replicable process.

The final version of our reporting guideline contains 27 items under 14 sub-headings. There is only one alternative to our reporting guideline, which is a 10-point checklist described by Group Wisdom [25]. The checklist provides limited guidance as to what to include in a report of Trochim’s concept mapping research [25]. The process of developing the guide has not been reported. We are therefore describing the first reporting guideline for Trochim’s concept mapping research. We intend to make the guideline freely available through the EQUATOR network.

Moher et al. (2011) has been critical of how researchers have developed reporting guidelines. In a systematic review of 81 reporting guidelines in health research, the authors noted that important aspects of guideline development are often not described [31]. For example, the process for developing the STROBE [32] reporting guidelines—an extensively used reporting guideline for observational research—does not provide details on how checklist items were derived. The process that we followed adhered strictly to the methods described by Moher et al. (2010) and endorsed by the Equator network [23]. Our protocol for developing the guideline was pre-registered, and deviations against the protocol have been fully described and justified. All data from our research—including the candidate concept maps—are available for checking (and reuse). Consequently, we consider that the ConMapT (Concept Mapping Trochim) guideline has been developed in a manner that is consistent with the best current practice.

The EQUATOR network does recommend using Delphi methodology to inform guideline development [24]. We did not do this, opting instead to use concept mapping. We made this decision, primarily because the methodology is well suited to the task and confers some notable advantages over the Delphi methodology. Concept mapping is more participatory as participants themselves generate ideas on the reporting items. It is also a valid critique of the Delphi methodology that there is a possibility that important information could be lost during the process of acquiring consensus among study participants [33,34,35].

The EQUATOR network recommends a systematic review to identify potential candidate reporting items and a Delphi approach to consolidate the guideline items. However, we choose to use concept mapping. Concept mapping is best suited to develop a better understanding of a complex phenomenon. However, the use of a concept mapping approach in guideline development has not been tested previously. Future guideline development may test the use of the concept mapping approach in guideline development.

Moher (2011) reported a systematic review of 81 reporting guidelines in health research [31]. The authors report that the median number of participants in guideline development was 22. While we invited 278 stakeholders, our guideline was developed with input from a maximum of 32 participants (although this varied somewhat across the different phases of concept mapping). Although the number of participants may be sufficient for the guideline development process, the modest response rate could have potentially introduced some non-response bias. Guideline development is a continuously evolving process. Some of the widely used guidelines like PRISMA have been reviewed and updated periodically. We recommend that our reporting guideline for concept mapping research be reviewed in the future and updated.

There are important limitations to our research that should be considered. There were two stakeholder groups—journal editors and research participants—that despite our best efforts, we were unable to recruit. We were not able to actively engage our expert group in drafting the reporting guideline; instead, they chose to provide written feedback on the draft we sent to them. The definition of a concept mapping expert that we used was somewhat arbitrarily: a researcher who had published at least five concept mapping papers. It may have been preferable to have recruited researchers who had published concept mapping methodological papers as they would likely have had a deeper knowledge of the approach. In addition, we did not solicit feedback from concept mapping methodological experts on the reporting guideline. A critical review of the checklist items from methodological experts could have enhanced the rigor of our guideline development. Future studies to refine the existing reporting guideline could engage methodological experts as part of this process.

The cluster solutions identified were based on the principal component analysis (PCA) and hierarchical cluster analysis (HCA). Trochim, however, suggests using Multidimensional Scaling (MDS) and Cluster Analysis (CA) for the analysis of concept mapping data [36]. Both PCA and MDS are well established for the analysis of multi-dimensional data and locate statements based on how frequently they are paired together [37]. However, there is an argument that the final concept map produced from these statistical procedures may be different [5]. We were unable to undertake a data analysis using MDS as the software we used for concept mapping (Ariadne) incorporates PCA for data analysis [30].

We removed one statement from the analysis of the concept map. Experts in concept mapping research who participated in the interpretation phase insisted on undertaking a data analysis after removing statements that made up individual clusters. We agree that rejecting the maps that contain clusters with only single statements may lead to the inadvertent omission of a unique item. However, as concept mapping is a participatory approach, we did not override what was suggested by our participants.

Concept mapping has been widely used in social sciences and other disciplines [11,38]. Our reporting guideline primarily focused on concept mapping in health research. Therefore, our reporting guideline may not comprehensively address the reporting needs of concept mapping research in other disciplines. Future research could focus on developing an extension of the reporting guideline to address the comprehensive reporting of concept mapping studies in other disciplines.

## 5. Conclusions

We have reported the development of a reporting guideline for concept mapping following the EQUATOR network’s recommended three-stage approach for guideline development. Stage 1, the systematic review, has been reported elsewhere. In this paper, we report on stages two and three. The final guideline consists of a checklist of 27 items, will be made freely available through the EQUATOR network, and can be accessed as Supplementary Document S12. By developing this guideline, we aim to improve the quality and transparency of the reporting of concept mapping research.

## Figures and Tables

**Table 1:** Demographic characteristics.

Characteristics	Brainstorming (n = 32)	Structuring (n = 24) ^1^	Interpretation (n = 4)
Age in years mean *(SD)*	49 (11)	49 (12)	58 (14)
Gender *(Female)*	23 (72%)	18 (75%)	4 (100%)
Country of Residence			
The USA	9 (28%)	7 (29%)	1 (25%)
Australia	5 (15%)	4 (17%)	-
The Netherlands	5 (15%)	4 (17%)	-
The UK	4 (13%)	3 (13%)	-
Canada	3 (9%)	2 (8%)	1 (25%)
Sweden	2 (6%)	1 (4%)	-
Others ^2^	4 (13%)	3 (13%)	2 (50%)
Highest completed education			
Doctoral Degree	29 (91%)	21 (88%)	4 (100%)
Postgraduate	3 (9%)	3 (12%)	-

^1^ Of the 24 participants, 22 completed sorting and 23 completed rating. ^2^ Others include France, Slovakia, South Africa, and Hong Kong.

## Data Availability

Data from the study can be publicly accessed from the institutional repository of Latrobe University (https://doi.org/10.26181/24415912.v1).

## Supplementary Materials

The following supplementary documents can be downloaded at: https://www.mdpi.com/article/10.3390/mps8020024/s1, S1: Copy of PICF used in the study, S2: Social media post used for recruitment of consumers, S3: Copy of the instructions sent to the participants explaining how to complete the structuring tasks, S4: Complete list of studies included in the systematic review, S5: Complete list of statements generated from the brainstorming, S6: Final list 96 statements generated from the brainstorming phase, S7: Point map with 96 statements, S8: List of 17 candidate concept maps with 96 statements, S9: List of 17 candidate concept maps produced after the removal of one statement, S10: List of 167 items used for drafting the reporting guideline, S11: Draft checklist sent to stakeholders for review and feedback, S12: Final draft checklist for ConMapT reporting guideline, S13: Results of the pilot study against each reporting guideline checklist item.
