# Supplementary material for: Development of a Reporting Guideline for Trochim’s Concept Mapping
Source: Methods Protoc. 2025 Mar 3;8(2):24. doi: 10.3390/mps8020024 (PMC11932253; doi:10.3390/mps8020024)
Supplement: Supplementary file 1 [file mps-08-00024-s001.zip › Supplementary document 1, PICF-researcher.pdf]

What are the items to be included in the report of a concept mapping study: a concept mapping study

This research is being carried out in partial fulfilment of PhD under the supervision of Professor Richard Gray. This study has been approved by La Trobe University human research ethics committee (ethics approval number ***). The following researchers will be conducting the study:		
**Role**	**Name**	**Organization**
Chief Investigator	Prof. Richard Gray	Theme Lead (Research and Engagement)<br>Deputy Vice-Chancellor Office<br>La Trobe University
Co-investigator	Associate Prof. Martin Jones	School of Nursing and Midwifery<br>La Trobe University
Student Investigator	Sandesh Pantha	School of Nursing and Midwifery<br>La Trobe University
**Research funder**	This research is supported by a La Trobe University studentship	

1. What is the study about?

The aim of this study is to develop a reporting guideline for a concept mapping study. We are interested to know views and experiences from the following colleagues: 1. Researchers that have led concept mapping studies, 2. Journal editors involved in publishing concept mapping studies, 3. Academic reviewers who have peer-reviewed a concept mapping manuscript, 4. Concept mapping research methodologists, 5. Consumers of concept mapping study (participant in a concept mapping study), and 6. Statisticians who have analysed the concept mapping study.

As a person previously involved in a concept mapping study, your views on what needs to be included in the report of a concept mapping study will be valuable for our research.

2. Why am I asked to participate?

We are asking you to participate in this study because you have published a concept mapping study as a corresponding author in a peer-reviewed journal.

3. Do I have to participate?

No, participating in this study is entirely voluntary.

4. What will I be asked to do?

We will ask you to complete two online tasks:

Task 1: involves a brainstorming (an interview) in which you will be asked to tell us your views about what items need to be included in the report of a concept mapping study. The interview will be completed using the ZOOM video conferencing. We anticipate that the interview will take about 30 minutes to complete.

Task 2: involves sorting items identified from the interviews - into groups or clusters. You will then be asked to rank these items into five groups in the order of importance (1 “least important” to 5 “most important”). It will probably take about an hour to complete these tasks using an online computer program called ARIADNE. We will send you a link and step by step instructions to complete the card sorting tasks.

5. How the research interview will be conducted?

We will conduct the interview by a video conferencing call. However, if you do not feel comfortable with a video call, we can carry out the interview by turning off the video.

Include information for card sorting task as well.

6. Will my interview be recorded?

Yes. We will audio record your interview using the recording facility in video conferencing package.

7. What are the benefits?

Findings from this study will help to develop a set of checklists to be used during the reporting of a concept mapping study. We will submit the guideline to be included with the enhancing the quality and transparency of health research (equator) network. The results of this study will help to improve the comprehensive reporting of a concept mapping study.

8. What are the risks?

We do not foresee any risks associated with this study. However, if you have any concerns, or if you experience something that you aren’t sure about, please contact us immediately so we can discuss the best way to manage your concerns.

Name/Organisation	Position	Telephone	Email
Richard Gray<br>La Trobe University	Theme Lead (Research and Engagement)<br>Deputy Vice-Chancellor Office	+61 3 9479 6852	r.gray@latrobe.edu.au
Martin Jones<br>La Trobe University	Associate Professor	+61401198633	martin.jones@unisa.edu.au
Sandesh Pantha<br>La Trobe University	PhD Candidate	+61466031345	s.pantha@latrobe.edu.au

9. What will happen to information about me?

We will comply with the La Trobe University Research Data Management Policy for the collection, storage and destruction of data collected during the study. This policy can be viewed online at

https://policies.latrobe.edu.au/document/view.php?id=106.

We will **collect and store** information about you in ways that will not reveal who you are. All information will be de-identified. We will not record any information about you in a paper record. All the information obtained in a digital form (including your consent form, demographic information, and interview records) will be stored in a password protected file in a secure University research drive.

We will **publish** information about you in ways that you will not be identified in any type of publication.

We will **keep** your information for five years after the project is completed. The data will be stored securely in a password protected research repository of La Trobe University (Figshare). Data will be destroyed after this time.

We will handle your personal information under the applicable privacy laws; any health information collected will be handled under the Health Records Act 2001 (Vic). You will have the right to access and correct your personal information by contacting the research team.

10. Will I hear about the results of the study?

Results from the study will be reported in a Ph.D. thesis submitted to the School of Nursing and Midwifery, La Trobe University. Findings from the study will also be reported in a peer-reviewed journal.

We will share a summary of the research findings via an email sent to all study participants.

11. What if I change my mind?

You have the right to decide not to be a part of the study. If you decide that you do not want to participate in the study, please let us know as soon as possible.

You can let us know by:

1. Completing the 'Withdrawal of Consent Form' (provided at the end of this document) or
2. Call us or
3. Email us

If you wish us to remove your data from the study, we can do this if you tell us within four weeks of your interview or within four weeks of completion of the clustering/prioritization task (task 2, as mentioned in section 4).

12. Who can I contact for questions or want more information?

If you would like to speak to us, please use the contact details below:

Name/Organisation	Position	Telephone	Email
Richard Gray<br>La Trobe University	Theme Lead (Research and Engagement)<br>Deputy Vice-Chancellor Office	+61 3 9479 6852	r.gray@latrobe.edu.au
Martin Jones<br>La Trobe University	Associate Professor	+61401198633	martin.jones@unisa.edu.au
Sandesh Pantha<br>La Trobe University	PhD Candidate	+61466031345	s.pantha@latrobe.edu.au

13. What if I have a complaint?

If you have a complaint about any part of this study, please contact:

Ethics Reference Number	Position	Telephone	Email
HEC***	Senior Research Ethics Officer	+61 3 9479 1443	humanethics@latrobe.edu.au

Consent Form – Declaration by Participant

I (the participant) have read (or, where appropriate, have had read to me) and understood the participant information statement, and any questions have been answered to my satisfaction. I agree to participate in the study, I know I can withdraw at any time until [four weeks] following the collection of my data. I agree information provided by me or with my permission during the project may be included in a thesis, presentation and published in journals on the condition that I cannot be identified.

I would like my information collected for this research study to be:

☐ Only used for this study

I agree to communicate with the research team with my email ____________________________________.

☐I understand that the study will help to understand the important items to be included in the report of a concept mapping study. Findings from the study will help to develop a checklist to enhance the reporting of a concept mapping study.

☐ I would like to receive a copy of the results via email or post. I have provided my details below and ask that they only be used for this purpose and not stored with my information or for future contact.

Participant Signature

☐ I have received a signed copy of the Participant Information Statement and Consent Form to keep

Participant's printed name	
Participant's signature	
Date	

Declaration by Researcher

☐ I have given a verbal explanation of the study, what it involves, and the risks and I believe the participant has understood

☐ I am a person qualified to explain the study, the risks and answer questions

Researcher's printed name	Sandesh Pantha
Researcher's signature	
Date	Saturday, 12 August 2023

* All parties must sign and date their own signature

Withdrawal of Consent

I wish to withdraw my consent to participate in this study. I understand withdrawal will not affect my relationship with La Trobe University of any other organisation or professionals listed in the Participant Information Statement. I understand the researchers cannot withdraw my information once it has been analysed.

I understand my information will be withdrawn as outlined below:

- ✓ Any identifiable information about me will be withdrawn from the study.
- ✓ The researchers cannot withdraw my information once it has been analysed.

I would like my already collected and unanalysed data

☐ Destroyed and not used for any analysis

☐ Used for analysis

Participant Signature

Participant's printed name	
Participant's signature	
Date	

Please forward this form to

CI Name	Richard Gray
Email	r.gray@latrobe.edu.au
Phone	+61 3 9479 6852
Postal Address	Level 3, George Singer Building<br>La Trobe University<br>Bundoora, VIC 3086
