# Supplementary material for: Development of a Reporting Guideline for Trochim’s Concept Mapping
Source: Methods Protoc. 2025 Mar 3;8(2):24. doi: 10.3390/mps8020024 (PMC11932253; doi:10.3390/mps8020024)
Supplement: Supplementary file 1 [file mps-08-00024-s001.zip › Supplementary document 10, List of 167 checklist item.pdf]

List of 167 items for checklist development

S. No	Origin	Component	Item
1	Systematic review	Title and abstract	Concept mapping was stated in the study title
2	Systematic review	Title and abstract	Concept mapping was reported as a methodology in the abstract
3	Systematic review	Title and abstract	Rationale (background information) of study was provided in the abstract
4	Systematic review	Title and abstract	Focus question/prompt was reported in the abstract
5	Systematic review	Title and abstract	Stakeholders who participated in the study were identified in the abstract
6	Systematic review	Title and abstract	Information on the phases of concept mapping was provided in the abstract
7	Systematic review	Title and abstract	Study site was reported in the abstract
8	Systematic review	Title and abstract	Number of participants in the study was provided in the abstract
9	Systematic review	Title and abstract	Information on total number of statements generated in the study is provided in the abstract
10	Systematic review	Title and abstract	Number of clusters in the concept map was reported in the abstract
11	Systematic review	Title and abstract	Label for all clusters was provided in the abstract
12	Systematic review	Title and abstract	Concept mapping software used in the study was stated in the abstract
13	Systematic review	Background	Rationale for the study was explained
14	Systematic review	Background	Rationale/Justification for concept mapping as a study design was provided
15	Systematic review	Background	A clear aim/objective of the study was reported
16	Systematic review	Methods - preparation	The development of the focus prompt was elaborated
17	Systematic review	Methods - preparation	Involvement of the stakeholders in the development of focus prompt was reported
18	Systematic review	Methods - preparation	Focus prompt used in the study was stated
19	Systematic review	Methods - preparation	All the stakeholder groups were identified in the manuscript
20	Systematic review	Methods - preparation	Rationale for the stakeholder groups was provided
21	Systematic review	Methods - preparation	Participant recruitment was elaborated
22	Systematic review	Methods - preparation	Inclusion and exclusion criteria was provided
23	Systematic review	Methods – preparation	Data collection period was reported in the manuscript
24	Systematic review	Methods - ideas generation	The process of idea generation was outlined
25	Systematic review	Methods - ideas generation	Rationale was provided for the number of participants in the idea generation phase

26	Systematic review	Methods - ideas generation	Information was provided on how brainstorming session was conducted (face-to-face, remote, or both)
27	Systematic review	Methods - ideas generation	The process of idea synthesis (statement reduction) was detailed
28	Systematic review	Methods - ideas generation	Involvement of stakeholders in idea synthesis was reported
29	Systematic review	Methods - structuring the statements	Rationale was provided for the number of participants engaged for structuring the statements
30	Systematic review	Methods - structuring the statements	Instructions for structuring the statements was reported
31	Systematic review	Methods - structuring the statements	Information on how statements were structured (face-to-face, remote, or both) was reported
32	Systematic review	Methods - structuring the statements	Web application/software used to structure the statements remotely was reported
33	Systematic review	Methods - structuring the statements	Information was provided on the duration of structuring of the statements
34	Systematic review	Methods - structuring the statements	Information was provided on the number of prioritization task and type of Likert scale
35	Systematic review	Methods - data analysis	Name of the software used for data analysis was reported
36	Systematic review	Methods - data analysis	Authors outline the steps (statistical procedures) involved in the analysis of concept mapping data
37	Systematic review	Methods - data analysis	Information was provided on how cluster solution was identified
38	Systematic review	Methods - data analysis	The process of providing cluster labels was reported
39	Systematic review	Methods - data analysis	Information was provided on who interpreted the data
40	Systematic review	Methods - data analysis	Study participants and or stakeholders were engaged in data interpretation
41	Systematic review	Additional information	Name of the review board providing ethics approval was mentioned
42	Systematic review	Additional information	Authors report the ethics approval number
43	Systematic review	Additional information	The process of obtaining consent from participants was reported
44	Systematic review	Additional information	Information on participant reimbursement was provided
45	Systematic review	Results - participants	Authors report the total number of participants in the study
46	Systematic review	Results - participants	Flow of participants through the different phases of concept mapping was provided
47	Systematic review	Results - participants	Sample size for idea generation was reported
48	Systematic review	Results - participants	Participant response rate for idea generation was stated

49	Systematic review	Results - participants	Number of participants who structured the statements was reported
50	Systematic review	Results - participants	Response rate was provided for the statement structuring phase of concept mapping
51	Systematic review	Results - participants	Demographic characteristics was reported for all stakeholder groups
52	Systematic review	Results - statements	Number (total) of statements generated by the participants was reported
53	Systematic review	Results - statements	The number of statements used for structuring phase was reported
54	Systematic review	Results - statements	Number of statements beyond those generated by participants was reported
55	Systematic review	Results - statements	List of statements used to generate the concept map was provided
56	Systematic review	Results - statements	Information was provided on the most and least important statements
57	Systematic review	Results - statements	Statements were classified on the based on a go-zone graph
58	Systematic review	Results - clusters	Number of clusters generated by the participants (example, mean) was reported
59	Systematic review	Results - clusters	The number of cluster solutions considered for interpretation was reported
60	Systematic review	Results - clusters	All clusters were identified in the report
61	Systematic review	Results - clusters	Authors provide characteristics of the clusters identified in the study
62	Systematic review	Results - clusters	The most and least important clusters were reported
63	Systematic review	Results - clusters	Authors report the cluster bridging value
64	Systematic review	Results - clusters	Information is provided on the stress value and its significance
65	Systematic review	Results - clusters	Information is provided on the number of statements in each cluster
66	Systematic review	Results - clusters	A ladder graph was computed to report prioritization between stakeholder groups or prioritization tasks
67	Systematic review	Discussion	Authors discuss the relevance of the study results
68	Systematic review	Discussion	A summary of findings from the study was provided
69	Systematic review	Discussion	The possible use of the results from the study was reported
70	Systematic review	Limitations	A discussion was provided on the limitations of the study
71	Systematic review	Registration & protocol	Study was pre-registered, or protocol was published before results
72	Concept mapping	CM cluster 1	An overview of the results [for each stakeholder group] per stage.
73	Concept mapping	CM cluster 1	Present the final number of statements included in the card sorting (clustering and ranking).
74	Concept mapping	CM cluster 1	Describe the final product [clusters and axis] of the concept mapping research.
75	Concept mapping	CM cluster 1	Make it clear to the readers how the map should be interpreted.
76	Concept mapping	CM cluster 1	Examples of the statements to demonstrate the individual clusters.
77	Concept mapping	CM cluster 1	Relationship of the cluster and statements [should be described]

78	Concept mapping	CM cluster 1	Provide a clear description of how cluster configuration was selected.
79	Concept mapping	CM cluster 2	The title and abstract clearly states the study used a concept mapping approach.
80	Concept mapping	CM cluster 2	The title and abstract describes the core problem being investigated.
81	Concept mapping	CM cluster 2	Explanation of why concept mapping [in background] is the right solution for this research question.
82	Concept mapping	CM cluster 2	Abstract has a clear description of the different participant cohorts.
83	Concept mapping	CM cluster 2	The abstract reflects on the methodological steps.
84	Concept mapping	CM cluster 2	The abstract reflects on the analytical approaches for the study.
85	Concept mapping	CM cluster 2	The abstract contains basic information about what we found.
86	Concept mapping	CM cluster 2	A manuscript/report has a good description of the relevant literature with proper references.
87	Concept mapping	CM cluster 2	Describe how the concept map will be utilized.
88	Concept mapping	CM cluster 2	Summary of how study findings fit with the bigger literature and help us.
89	Concept mapping	CM cluster 2	The conclusion is a summary of the core findings from the study.
90	Concept mapping	CM cluster 3	Provide some examples of what the authors felt was redundant or duplicate statements.
91	Concept mapping	CM cluster 3	Information about the total number of statements generated from the participants.
92	Concept mapping	CM cluster 3	If we have a go-zone plot, include in a table into which quadrant each item falls.
93	Concept mapping	CM cluster 3	Look for patterns within the clusters/whole data.
94	Concept mapping	CM cluster 3	Talk about cluster thickness to show the relative importance of each cluster.
95	Concept mapping	CM cluster 3	Give a few examples of the cluster range data (least and most important clusters).
96	Concept mapping	CM cluster 3	Report on ladder plot if we want to see the comparison between stakeholders.
97	Concept mapping	CM cluster 3	Report a higher-order interpretation of the map (if done).
98	Concept mapping	CM cluster 5	Some supplementary data to clearly present how the statement synthesis process was done.
99	Concept mapping	CM cluster 5	Justify the rationale behind the rating scale.
100	Concept mapping	CM cluster 5	Information on the Likert scale used for rating question.
101	Concept mapping	CM cluster 5	Information on incomplete or excluded data is provided.
102	Concept mapping	CM cluster 5	Note on how many cluster solutions were reviewed before the final solution was determined.
103	Concept mapping	CM cluster 5	Any adjustments made in the cluster map should be reported.
104	Concept mapping	CM cluster 5	Note of how we managed outstanding items (during data analysis) that do not belong to any clusters.
105	Concept mapping	CM cluster 6	Description of how we assessed the saturation of the conceptual space.
106	Concept mapping	CM cluster 6	Talk about the minimum sample size to have reliable structuring data.

107	Concept mapping	CM cluster 7	A detailed description/justification of the phases of concept mapping that may include a flowchart.
108	Concept mapping	CM cluster 7	The planning phase of the study is clearly described.
109	Concept mapping	CM cluster 7	Information on working with an advisory group (if involved).
110	Concept mapping	CM cluster 7	The initial question or focus prompt used in the study is clearly (explicitly) defined.
111	Concept mapping	CM cluster 7	Justify the different stakeholder groups included in each stage.
112	Concept mapping	CM cluster 7	Describe the contribution of the stakeholder groups during different phases of the study.
113	Concept mapping	CM cluster 7	Explain how researchers ensured broad representation within the stakeholder groups.
114	Concept mapping	CM cluster 7	Report if anybody influenced the selection of the participants.
115	Concept mapping	CM cluster 7	A concept mapping research is transparent about the power dynamics.
116	Concept mapping	CM cluster 7	Any issues on language translation are to be reported.
117	Concept mapping	CM cluster 7	If statements are returned to participants for validation, we should note which group was involved.
118	Concept mapping	CM cluster 7	Report the exact wording of the statements used for card sorting tasks.
119	Concept mapping	CM cluster 7	Justify why stakeholder groups were not involved in interpreting the map.
120	Concept mapping	CM cluster 7	Any deviations from the study protocol are explained/justified.
121	Concept mapping	CM cluster 7	Ethical considerations are detailed and discussed.
122	Concept mapping	CM cluster 7	Information on decisions to remunerate the participants.
123	Concept mapping	CM cluster 8	The actual experience of the concept mapping process is talked in the discussion.
124	Concept mapping	CM cluster 8	Talk about the limitations of the concept mapping project/process.
125	Concept mapping	CM cluster 9	Information on how credibility, trustworthiness was applied in interviews conducted during study.
126	Concept mapping	CM cluster 9	Provide some details on how much interaction occurred within the groups.
127	Concept mapping	CM cluster 9	Information on how the card sorting [clustering and ranking] data is used in analyses.
128	Concept mapping	CM cluster 9	Information about who was involved in interpreting the clusters.
129	Concept mapping	CM cluster 9	Provide details on the origin of all statements.
130	Concept mapping	CM cluster 9	Was there any feedback on the final cluster solution from the stakeholders?
131	Concept mapping	CM cluster 9	The extent to which sorted material was managed or edited by the research team.
132	Concept mapping	CM cluster 10	Describe how (hierarchical) cluster analysis was conducted.
133	Concept mapping	CM cluster 10	The authors mention underlying analytical steps carried out [statistical algorithm] in software.
134	Concept mapping	CM cluster 10	Information on multi-dimensional scaling.
135	Concept mapping	CM cluster 10	Applying the split-half reliability test to measure the validity of a map.

136	Concept mapping	CM cluster 10	Did you do any sensitivity analysis?
137	Concept mapping	CM cluster 10	Any further analysis specific to a software package is reported.
138	Concept mapping	CM cluster 10	Use Cronbach's alpha for an estimate of internal consistency.
139	Concept mapping	CM cluster 10	Presenting the mean and standard deviations of statements included in the prioritisation task.
140	Concept mapping	CM cluster 10	Report the mean and the range of the number of groups [clusters] generated by the participants.
141	Concept mapping	CM cluster 10	Further analysis of the content within the cluster to identify the pattern in the data.
142	Concept mapping	CM cluster 10	Present the mean value for each cluster.
143	Concept mapping	CM cluster 10	Report an R-value if the authors conduct an item level rating analysis for each cluster.
144	Concept mapping	CM cluster 10	Present the stress value (with interpretation) for the map.
145	Concept mapping	CM cluster 10	Show the eigenvalue of the eigenvectors.
146	Concept mapping	CM cluster 10	Report a test of significance to the rank order data of the ladder (pattern match) graph.
147	Concept mapping	CM cluster 10	If authors conduct means tests between clusters, they need to report t-test output.
148	Concept mapping	CM cluster 10	The correlation coefficient can be helpful if we are looking at different rating scales.
149	Concept mapping	CM cluster 11	What was the process of developing the research/focus question?
150	Concept mapping	CM cluster 11	Report on the details of pilots (if performed) to get our research prompt.
151	Concept mapping	CM cluster 11	Approach used for [recruitment of] participant groups at each phase of concept mapping is explicitly stated.
152	Concept mapping	CM cluster 11	A timeframe of how long to complete the individual stages.
153	Concept mapping	CM cluster 11	How do we collect their [participants] demographic data?
154	Concept mapping	CM cluster 11	Each of the methods used to generate the ideas [brainstorming] is carefully described.
155	Concept mapping	CM cluster 11	Information on whether any [brainstorming] sessions was recorded.
156	Concept mapping	CM cluster 11	Talk about the [number of] brainstorming sessions.
157	Concept mapping	CM cluster 11	Information on who facilitated the interpretation session.
158	Concept mapping	CM cluster 11	Was there any warm-up activities prior to idea generation?
159	Concept mapping	CM cluster 11	Describe the role of the moderator of the brainstorming session.
160	Concept mapping	CM cluster 11	Talk about the training (of the researchers) on group concept mapping.
161	Concept mapping	CM cluster 11	Information on how much detailing [level of support] was required during brainstorming/card sorting.
162	Concept mapping	CM cluster 11	Report how brainstorming data was transcribed/translated.
163	Concept mapping	CM cluster 11	Information on how interview data was processed and made into statements.
164	Concept mapping	CM cluster 11	Instructions provided to the participants [for different tasks] is clearly described.

165	Concept mapping	CM cluster 11	Explain how the card sorting sessions were conducted.
166	Concept mapping	CM cluster 11	Report how card sorting (prioritising and rating) activities are sequenced.
167	Concept mapping	Statement removed from cluster analysis	Provide information on the use of concept mapping software at different phases of the study
