# Supplementary material for: Development of a Reporting Guideline for Trochim’s Concept Mapping
Source: Methods Protoc. 2025 Mar 3;8(2):24. doi: 10.3390/mps8020024 (PMC11932253; doi:10.3390/mps8020024)
Supplement: Supplementary file 1 [file mps-08-00024-s001.zip › Supplementary document 11, Draft checklist sent to participants for feedback.pdf]

Draft checklist sent to participants for feedback.

**Phase of Concept mapping**	**Section and topic**	**Item #**	**Checklist item**
Title	Title	1	State the report as a concept mapping study .
Abstract	Abstract	2a	Describe the core problem that is the focus of the research.
		2b	State concept mapping as the study methodology.
		2c	Give a brief description of the site(s) where fieldwork was conducted.
		2d	State the stakeholder groups involved in the research.
		2e	List which phases of concept mapping were undertaken.
		2f	Give a description of the final concept map, including names of individual clusters.
Background	Rationale	3a	Give a sound scientific rationale for the study.
	Aim	3b	State the aim of the study.
Methods	Methodological justification	4	Provide a justification for using concept mapping as a methodology.
	Phases	5	State which of the six phases of concept mapping were undertaken and justify any deviations.
Preparation phase	Determining the focus prompt	6a	Describe the process for developing the focus prompt.
		6b	State the focus prompt.
		6c	Describe which stakeholder groups were involved in the development of focus prompt.
	Participants	7a	Justify how stakeholder group(s) were determined.
		7b	Describe how each stakeholder group was recruited to the study.
		7c	State eligibility criteria for each stakeholder group.
		7d	Provide a rationale for the sample size in each phase of concept mapping.
		8a	Describe the flow of participants through each phase of concept mapping (ideally as a flow diagram).
		8b	Tabulate and summarise the demographic and clinical (if relevant) characteristics of each stakeholder group.

	Public involvement	9a	State how the public or other stakeholder groups were involved in the research.
	Software packages	10	State which software packages were used in the research and to what purpose (e.g., Ariadne, Concept Systems, Stata).
Idea generation phase	Brainstorming	11a	State methods used for idea generation (e.g., brainstorming, literature review).
		11b	Describe how the brainstorming sessions were conducted and recorded.
		11c	Explain the process for generating statements from brainstorming sessions.
		11d	Describe factors (e.g., interpersonal disagreements) that may have negatively impacted idea generation during brainstorming sessions.
		12	State the maximum number of statements permitted in the structuring phase of concept mapping.
	Statement reduction	13a	Describe and justify the procedure for statement reduction (e.g., eliminating overlapping statements, combining similar statements).
		13b	State that participant wording was retained are far as possible.
		13c	Describe how stakeholders were involved in statement reduction.
		14	Report the total number of statements generated during the idea generation phase. Ensure a complete list of statements is available to the reader (e.g., as a supplementary document or data file).
Structuring statements phase	Prioritisation and clustering	15a	Describe the procedure for structuring statements (prioritising and clustering tasks).
		15b	Specify the instructions provided to the participants about how to complete prioritising and clustering tasks.
		16a	For each statement report the mean prioritisation score (consider reporting as a supplementary document or data file).
		16b	State the mean number of clusters generated by participants.
Data analysis phase	Data cleaning	17a	Describe procedures for data checking and cleaning.
		17b	Describe procedures for handling any missing data.
	Statistical procedures	18a	Specify what statistical procedures were undertaken.
		18b	Specify what statistical tests were undertaken to determine the validity of the concept map.
	Additional data analysis	19a	State any additional planned analyses (e.g., Go-zone graph).
		19b	State any unplanned analyses not in the study protocol.

Data interpretation phase	Data interpretation process	20a	Specify the process for determining the final cluster solution.
		20b	Specify the process for finalising cluster labels.
		20c	Specify, if stakeholders provided feedback on the final cluster solution.
	Concept map	21	Provide a detailed description of the final concept map.
	Clusters	22a	Describe each cluster in the final concept map using illustrative statements.
		22b	State if any post-hoc adjustments were made to the concept map (for example, moving statements from one cluster to another or combining clusters).
Utilization phase	Utilization	23	Provide a statement on how the concept map can be utilised.
Discussion	Discussion	24	Locate the concept map within the context of previous evidence.
Limitations	Limitations	25a	State all study limitations.
		25b	Describe and justify any deviation between the study protocol and report.
Ethics	Ethics	26a	State which ethics (or Institutional Review Board) approved the study (include ethics committee reference number).
		26b	Describe the procedure for obtaining informed consent from stakeholders.
		26c	State how stakeholders were compensated for participating (e.g., cash payment, gift card)
Conclusion	Conclusion	27	Summarise the key findings from the study.
Registration and protocol	Registration and protocol	28a	State if the study was prospectively registered before the first stakeholder was recruited.
		28b	Provide information on where the study protocol can be accessed (if available).
