# Supplementary material for: Development of a Reporting Guideline for Trochim’s Concept Mapping
Source: Methods Protoc. 2025 Mar 3;8(2):24. doi: 10.3390/mps8020024 (PMC11932253; doi:10.3390/mps8020024)
Supplement: Supplementary file 1 [file mps-08-00024-s001.zip › Supplementary document 12, ConMapT reporting guideline checklist items.pdf]

Checklist items for reporting Trochim's concept mapping research (ConMapT)

Heading	Section and topic	Item #	Checklist item	Page
Title	Title	1	State that the manuscript is reporting a concept mapping study.	
Abstract	Abstract	2a	Describe the core problem (focus) of the research.	
		2b	State concept mapping as the study methodology and list the phases of concept mapping* undertaken.	
		2c	Indicate where the fieldwork was conducted.	
		2d	State the stakeholder groups involved in the research. Report the total number of participants involved in the study.	
		2e	Give a brief description of the final concept map that should include cluster labels.	
Background	Rationale	3	Provide a sound scientific rationale for the study.	
	Aim	4	State the study aim(s).	
	Methodological justification	5a	Provide a justification for using concept mapping to address study aim(s).	
		5b	State which of the six phases* of concept mapping were undertaken. Justify why phase(s) of concept mapping were omitted.	
Phase 1, Preparation	Determining the focus prompt	6a	State the focus prompt for the study.	
		6b	Describe the process for developing the focus prompt and, if applicable, how stakeholders were involved.	
	Participants	7a	Justify why each stakeholder group was selected.	
		7b	State the eligibility criteria for each stakeholder group.	
		7c	Describe how study participants were recruited.	
		7d	Provide a rationale for the number of participants at each phase of concept mapping.	
		8a	Describe the flow of participants (ideally as a flow diagram) through each phase of concept mapping.	
		8b	Tabulate and describe the demographic and clinical (if relevant) characteristics of each of the stakeholder groups.	
	Public involvement	9	Explain how patients and/or the public were involved in each phases of the research (from conception through dissemination).	
	Software package	10	State which software package (e.g., Ariadne, Group wisdom [formally known as concept systems], Stata) was used in each phase of concept mapping.	

Phase 2, Generating the ideas	Brainstorming	11a	State all methods used for idea generation (e.g., brainstorming, literature review).	
		11b	Describe in detail how brainstorming sessions were conducted (e.g., individual interviews, focus groups) and recorded.	
		11c	Explain how data from the brainstorming sessions was turned into statements.	
		11d	Describe factors that may have influenced (e.g. group dynamics) idea generation.	
		11e	Report the number of statements generated from each approach to idea generation (literature review, brainstorming).	
	Statement reduction	12a	Describe and justify the procedure for statement reduction (e.g., eliminating overlapping statements, or combining similar statements).	
		12b	Describe to what extent participant wording was retained in the statements.	
		12c	Describe how stakeholders were involved in statement reduction.	
		13	Report the final number of statements following statement reduction. Ensure a complete list of the statements is available (e.g., as a supplementary document or data file).	
Phase 3, Structuring the statements	Clustering and rating (or prioritisation**)	14a	Describe the procedures followed for structuring statements (clustering and rating tasks).	
		14b	Provide information about the instructions to participants as to how to complete the clustering and rating tasks.	
		15	Report the average (mean, median) number of clusters generated by the participants.	
		16	Report the mean rating score for each statement (consider reporting as a supplementary document or data file).	
Phase 4, Concept mapping analysis	Data cleaning	17a	Describe procedures for data checking and cleaning.	
		17b	Describe procedures for handling missing data.	
	Statistical procedures	18a	Specify what statistical procedures were undertaken to generate candidate concept maps.	
		18b	Specify the statistical tests undertaken to determine the validity of the concept map (e.g., stress value, split-half reliability test).	
		18c	Describe any additional analyses not in the study protocol.	
Phase 5, Interpreting the map	Data interpretation process	19a	State how the final concept map was selected.	
		19b	Provide a description of how cluster (on the final concept map) labels were determined.	
		19c	Describe how stakeholders provided feedback on the final concept map.	

	Description of the final concept map	20a	Describe the final concept map.	
		20b	State and justify any post hoc adjustments to the concept map (e.g., moving statements between or combining clusters).	
		20c	Describe each cluster (including illustrative statements) in the final concept map.	
	Rating data	21	If applicable, describe the go-zone (include examples of statements in each quadrant), or pattern match (ladder graph).	
Phase 6, Utilization	Utilization	22	Provide a statement on how the concept map will be utilised.	
Discussion	Discussion	23	Locate the concept map within the context of existing evidence.	
Limitations	Limitations	24	State all study limitations (including those that may have arisen because of deviations from the study protocol).	
Ethics	Ethics	25a	State which ethics committee (or Institutional Review Board) reviewed the study (include the ethics committee reference number).	
		25b	Describe the ethical issues in the study.	
		25c	Describe the procedure for obtaining informed consent from the study participants.	
		25d	State how participants were compensated for taking part in the study.	
Conclusion	Conclusion	26	Summarise the key findings from the study.	
Registration and protocol	Registration and protocol	27a	Give details of the registration status of the study (including registry and registration number)	
		27b	State how the study protocol can be accessed.	
		27c	List any deviations from the study protocol.	

* Six phases of concept mapping methodology, described by Trochim (Kane & Trochim, 2007) are 1. Preparation, 2. Ideas generation, 3. Structuring of statements, 4 Data analysis, 5. Interpretation, and 6. Utilization of maps;

** In some descriptions of concept mapping prioritising is described as rating.

Please refer to the following citation for detailed information on the concept mapping process:

Kane, M., & Trochim, W. (2007). *Concept mapping for planning and evaluation* (Vol. 50). Sage Publications. https://doi.org/10.4135/9781412983730
