# Supplementary material for: Development of a Reporting Guideline for Trochim’s Concept Mapping
Source: Methods Protoc. 2025 Mar 3;8(2):24. doi: 10.3390/mps8020024 (PMC11932253; doi:10.3390/mps8020024)
Supplement: Supplementary file 1 [file mps-08-00024-s001.zip › Supplementary document 13, Pilot test of ConMap checklist items.pdf]

Results from the pilot test of the ConMap checklist items

Item #	Item Name	Beckers et. al. (2018)[1]	Buitenweg et. al. (2018)[2]	Chow et. al. (2021)[3]	Hvidt et. al. (2020)[4]	Keita et et. al. (2016)[5]	Klokker et. al. (2015)[6]	Lobb et et. al. (2013)[7]	McCaffey et. al. (2019)[8]	Nielsen et. al. (2019)[9]	Soule et. al. (2020)[10]
1	State the manuscript is reporting a concept mapping study.	Yes	Yes	Yes	Yes	Yes	No	Yes	Yes	No	No
2a	Describe the core problem (focus) of the research.	Yes	Yes	No	Yes	Yes	No	Yes	Yes	Yes	Yes
2b	State concept mapping as the study methodology and list the phases of concept mapping undertaken.	Yes	Yes	Yes	Yes	Yes	Yes	Yes	Yes	Yes	Yes
2c	Indicate where the fieldwork was conducted.	Yes	No	Yes	Yes	No	Yes	Yes	Yes	No	No
2d	State the stakeholder groups involved in the research. Report the total number of participants involved in the study	Yes	Yes	Yes	Yes	Yes	Yes	Yes	Yes	Yes	Yes
2e	Give brief description of the final concept map that should include cluster labels.	Yes	Yes	Yes	Yes	Yes	Yes	Yes	Yes	Yes	Yes
3	Give a sound scientific rationale for the study.	Yes	Yes	Yes	Yes	Yes	Yes	Yes	Yes	Yes	Yes
4	State the study aim(s).	No	Yes	Yes	Yes	Yes	Yes	No	Yes	Yes	Yes
5a	Provide a justification for using concept mapping to address the study aim(s).	Yes	No	Yes	Yes	Yes	Yes	No	Yes	Yes	Yes
5b	State which of the six phases[1] of concept mapping were undertaken. Justify why any phase(s) of concept mapping were omitted.	Yes	Yes	Yes	Yes	Yes	Yes	Yes	Yes	Yes	Yes
6a	State the focus prompt for the study.	Yes	Not applicable	Not applicable	Yes	Yes	Yes	Yes	Yes	Yes	Yes
6b	Describe the process for developing the focus prompt and, if applicable, how stakeholders were involved.	No	Not applicable	Not applicable	No	No	No	No	No	No	No
7a	Justify why each stakeholder group was selected.	Yes	No	Yes	Yes	Yes	No	No	No	No	No
7b	State eligibility criteria for each stakeholder group.	No	No	Yes	No	Yes	Yes	No	Yes	No	Yes
7c	Describe how study participants were recruited.	No	Yes	No	No	Yes	Yes	No	Yes	Yes	Yes
7d	Provide a rationale for the number of participants at each phase of concept mapping.	No	No	Yes	No	No	No	No	No	Yes	No

8a	Describe the flow of participants (ideally as a flow diagram) through each phase of concept mapping.	Yes	Yes	Yes	Yes	Yes	Yes	Yes	Yes	Yes	Yes
8b	Tabulate and describe the demographic and clinical (if relevant) characteristics of each stakeholder group.	Yes	Yes	Yes	Yes	Yes	Yes	No	Yes	Yes	Yes
9	Explain how patients and/or the public were involved in each phase of the research (from conception through dissemination).	No	Yes	No	Yes	Yes	No	No	No	No	No
10	State which software package (e.g., Ariadne, Group wisdom [formally known as concept systems], Stata) was used in each phase of concept mapping.	Yes	Yes	Yes	Yes	Yes	Yes	Yes	Yes	Yes	Yes
11a	State all methods used for idea generation (e.g., brainstorming, literature review).	Yes	Yes	Yes	Yes	Yes	Yes	Yes	Yes	Yes	Yes
11b	Describe in detail how brainstorming sessions were conducted (e.g., individual interviews, focus groups) and recorded.	Not applicable	Yes	Yes	Yes	Yes	Yes	Yes	Yes	Yes	Yes
11c	Explain how data from the brainstorming sessions were turned into statements.	Not applicable	Not applicable	No	No	No	No	No	No	Yes	No
11d	Describe factors that may have influenced (e.g., group dynamics) idea generation.	Not applicable	Not applicable	Not applicable	Not applicable	No	No	No	No	No	Not applicable
11e	Report the number of statements generated from each approach to idea generation (literature review, brainstorming).	Yes	Yes	No	Yes	Yes	Yes	Yes	Yes	Yes	Yes
12a	Describe and justify the procedure for statement reduction (e.g., eliminating overlapping statements, or combining similar statements).	Yes	Yes	No	Yes	Yes	Yes	Yes	Yes	Yes	Yes
12b	Describe to what extent participant wording was retained in the statements.	No	No	No	No	No	No	No	Yes	No	No
12c	Describe how stakeholders were involved in statement reduction.	Yes	No	No	No	No	No	No	No	No	No
13	Report the final number of statements following statement reduction. Ensure a complete list of the statements is available (e.g., as a supplementary document or data file).	List of statement not provided	Yes	List of statement not provided	List of statement not provided	List of statement not provided	No	List of statement not provided	List of statement not provided	List of statement not provided	No
14a	Describe the procedures followed for structuring statements (clustering and rating tasks).	Yes	Yes	Yes	Yes	Yes	Yes	Yes	Yes	No	Yes

14b	Provide information about the instructions to participants as to how to complete the clustering and rating tasks.	Yes	Yes	No	No	Yes	No	Yes	No	No	Yes
15	Report the average (mean, median) number of clusters generated by participants.	No	Yes	No	Yes	Yes	No	No	No	No	No
16a	Report the mean rating score for each statement (consider reporting as a supplementary document or data file).	Yes	Not applicable	Yes	Yes	Yes	No	No	No	Yes	Yes
17a	Describe procedures for data checking and cleaning.	No	No	No	Yes	No	No	No	No	Yes	Yes
17b	Describe procedures for handling missing data.	No	No	No	Yes	No	No	No	No	Yes	No
18a	Specify what statistical procedures were undertaken to generate concept maps.	Yes	Yes	Yes	Yes	Yes	No	No	No	Yes	Yes
18b	Specify the statistical tests undertaken to determine the validity of the concept map (e.g., stress value, split-half reliability test).	Yes	Yes	Yes	No	Yes	No	Yes	No	No	No
18c	Describe any additional analyses not in the study protocol.	Yes	No	No	No	Yes	No	No	No	No	No
19a	State how the final concept map was selected.	Yes	Yes	No	Yes	Yes	Yes	Yes	Yes	No	No
19b	Provide a description of how cluster (on the final concept map) labels were determined.	No	Yes	No	Yes	Yes	No	No	Yes	No	No
19c	State how stakeholders provided feedback on the final concept map.	No	No	No	Yes	No	No	Yes	No	Yes	No
20a	Describe the final concept map.	Yes	Yes	No	Yes	Yes	Yes	Yes	Yes	Yes	Yes
20b	State and justify any post hoc adjustments made to the concept map (e.g., moving statements between or combining clusters).	No	No	No	Yes	No	Yes	Yes	No	Yes	No
20c	Describe each cluster (using illustrative statements) in the final concept map.	Yes	Yes	No	No	Yes	Yes	No	Yes	Yes	Yes
21	If applicable, describe the go-zone (include examples of statements in each quadrant), or pattern match (ladder graph).	No	No	No	No	Yes	No	No	Yes	No	Yes
22	Provide a statement on how the concept map will be utilised.	No	No	No	No	No	Yes	Yes	No	Yes	No
23	Locate the concept map within the context of existing evidence.	Yes	Yes	Yes	Yes	Yes	Yes	Yes	Yes	Yes	Yes
24	State all study limitations (including those that may have arisen because of deviations from the study protocol).	Yes	Yes	Yes	Yes	Yes	No	Yes	Yes	Yes	Yes

25a	State which ethics committee (or Institutional Review Board) reviewed the study (include reference number).	Yes	Yes	Yes	Yes	Yes	No	No	Yes	No	No
25b	Describe the ethical issues in the study.	No	No	No	No	No	No	No	No	Yes	No
25c	Describe the procedure for obtaining informed consent from the study participants.	No	No	No	No	No	No	No	Yes	No	No
25d	State how participants were compensated for taking part in the study.	No	No	Yes	Yes	Yes	Yes	No	Yes	No	Yes
26	Summarise the key findings from the study.	Yes	Yes	Yes	Yes	Yes	Yes	Yes	No	Yes	Yes
27a	Give details of the registration status of the study (including registry and registration number)	No	No	No	No	No	No	No	No	No	No
27 b	State how the study protocol can be accessed.	No	No	No	No	No	No	No	No	No	No
27c	List any deviations from the study protocol.	No	No	No	No	No	No	No	No	No	No

References

1. Beckers T, Koekkoek B, Hutschemaekers G, Tiemens B. Potential predictive factors for successful referral from specialist mental-health services to less intensive treatment: A concept mapping study. PloS one. 2018;13(6):e0199668.
2. Buitenweg DC, Bongers IL, van de Mheen D, van Oers HA, Van Nieuwenhuizen C. Worth a thousand words? V isual concept mapping of the quality of life of people with severe mental health problems. International journal of methods in psychiatric research. 2018;27(3):e1721.
3. Chow KM, Chan CWH, Choi KC, White ID, Siu KY, Sin WH. A practice model of sexuality nursing care: a concept mapping approach. Supportive care in cancer : official journal of the Multinational Association of Supportive Care in Cancer. 2021;29(3):1663-73.
4. Hvidt NC, Nielsen KT, Kørup AK, Prinds C, Hansen DG, Viftrup DT, et al. What is spiritual care? Professional perspectives on the concept of spiritual care identified through group concept mapping. BMJ open. 2020;10(12):e042142.
5. Keita AD, Whittaker S, Wynter J, Kidanu TW, Chhay C, Cardel M, et al. Applying concept mapping methodology to identify the perceptions of risk and protective factors for childhood obesity among Southeast Asian refugees. Journal of health care for the poor and underserved. 2016;27(4):1909-33.
6. Klokker L, Osborne R, Wæhrens EE, Norgaard O, Bandak E, Bliddal H, et al. The concept of physical limitations in knee osteoarthritis: as viewed by patients and health professionals. Quality of life research. 2015;24:2423-32.
7. Lobb R, Pinto AD, Lofters A. Using concept mapping in the knowledge-to-action process to compare stakeholder opinions on barriers to use of cancer screening among South Asians. Implementation Science. 2013;8(1):1-12.
8. McCaffrey SA, Chiauzzi E, Chan C, Hoole M. Understanding 'good health care' from the patient's perspective: Development of a conceptual model using group concept mapping. The Patient: Patient-Centered Outcomes Research. 2019;12(1):83-95.
9. Nielsen KT, Klokker L, Guidetti S, Waehrens EE. Identifying, organizing and prioritizing ideas on how to enhance ADL ability. Scandinavian journal of occupational therapy. 2019;26(5):382-93.
10. Soule EK, Mayne S, Snipes W, Guy MC, Breland A, Fagan P. Impacts of COVID-19 on Electronic Cigarette Purchasing, Use and Related Behaviors. International journal of environmental research and public health. 2020;17(18).
