# Supplementary material for: Development of a Reporting Guideline for Trochim’s Concept Mapping
Source: Methods Protoc. 2025 Mar 3;8(2):24. doi: 10.3390/mps8020024 (PMC11932253; doi:10.3390/mps8020024)
Supplement: Supplementary file 1 [file mps-08-00024-s001.zip › Supplementary document 2, Social media advert.pdf]

LA TROBE UNIVERSITY

Have you ever participated in a concept mapping study? We are developing a reporting guideline for concept mapping research and would like to get your input. It's a bit weird, but we are doing a concept mapping study to inform the development of a concept mapping reporting guideline. If you can spare us one and half hour, please email (s.pantha@latrobe.edu.au) or text (0466031345) me and I can send you some more information about the study.

(This study has been approved by La Trobe human research ethics committee.
Ethics approval number. **HEC****)

LA TROBE UNIVERSITY

Have you ever participated in a concept mapping study? We are developing a reporting guideline for concept mapping research and would like to get your input.

It's a bit weird, but we are doing a concept mapping study to inform the development of a concept mapping reporting guideline.

If you can spare us one and half hour, please email (s.pantha@latrobe.edu.au) or text (0466031345) me and I can send you some more information about the study.

(This study has been approved by La Trobe human research ethics committee: ethics approval number. **HEC****)
