# Supplementary material for: Development of a Reporting Guideline for Trochim’s Concept Mapping
Source: Methods Protoc. 2025 Mar 3;8(2):24. doi: 10.3390/mps8020024 (PMC11932253; doi:10.3390/mps8020024)
Supplement: Supplementary file 1 [file mps-08-00024-s001.zip › Supplementary document 3, Instructions for sorting and ranking task.pdf]

Step by step guide on completing task using ARIADNE

Once we click the link sent in the email, we will see the following page.

We can see two red boxes for the two tasks. We can begin either of the two tasks. Our step by step guide will provide guidance for both tasks. However, we will begin with the prioritizing task (mentioned as importance in the first red box).

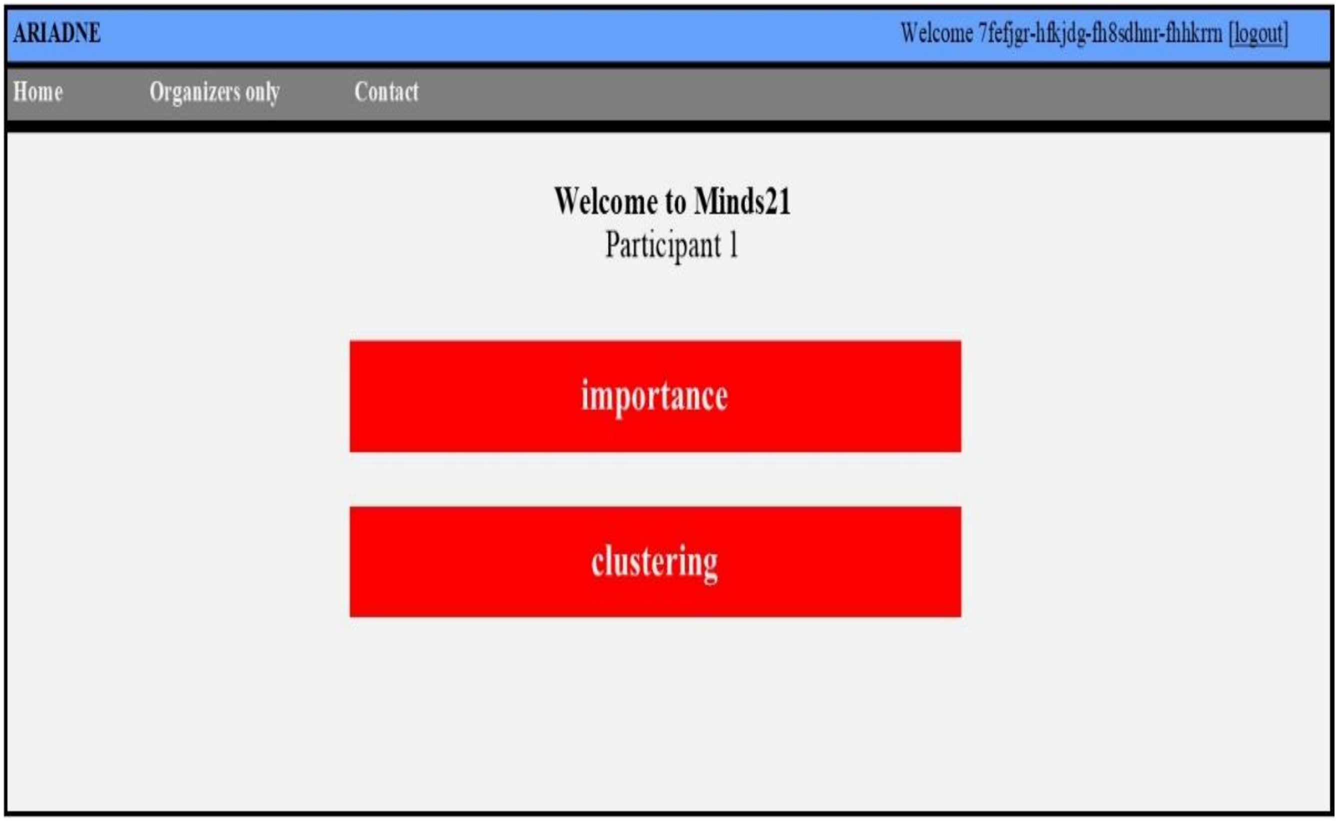

Figure 1: Ariadne programme home page

Here on the top right corner we can see 'logout'. Click it if you want to logout at this time and visit the program at a later time.

Task 1: Prioritization

In this task, we will divide the individual statements on nurse-doctor communication into five equal groups from 1 'least important' to 5 'most important'.

Step 1: Click on "Importance"

Once we click the importance (figure 1), it will take us to this page (figure 2).

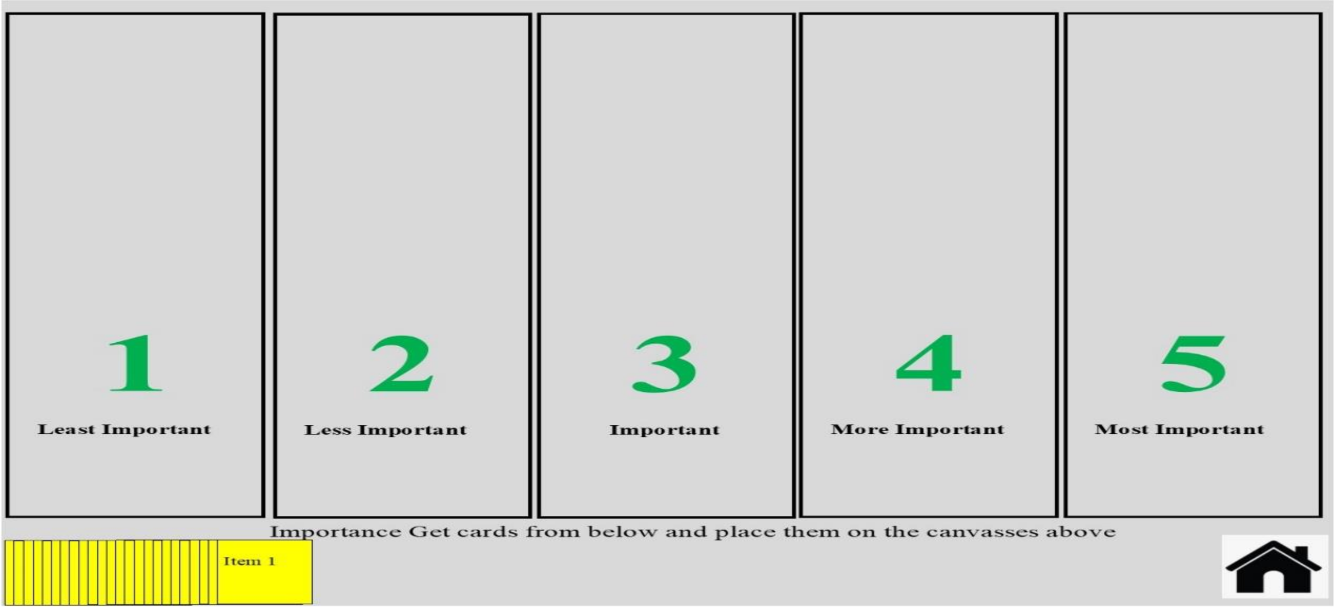

Figure 2: Prioritizing task home page

Here we can see five vertical coulumns and a pack of yellow cards (at the lower left corner) containing the statements generated from the interview. The 'home ' button at the lower right corner allows us to exit.

Step 2: Drag the cards into the vertical columns

We need to drag each card from the pile and place it into In one of the five vertical columns. These cards need to be organized into the columns based on what you think is important for nurse-doctor communication. The column at the left represents 'least important' whereas the column at the extreme right represents 'most important'. In figure 3, we can see that items 1-19 have been moved from the deck into the columns.

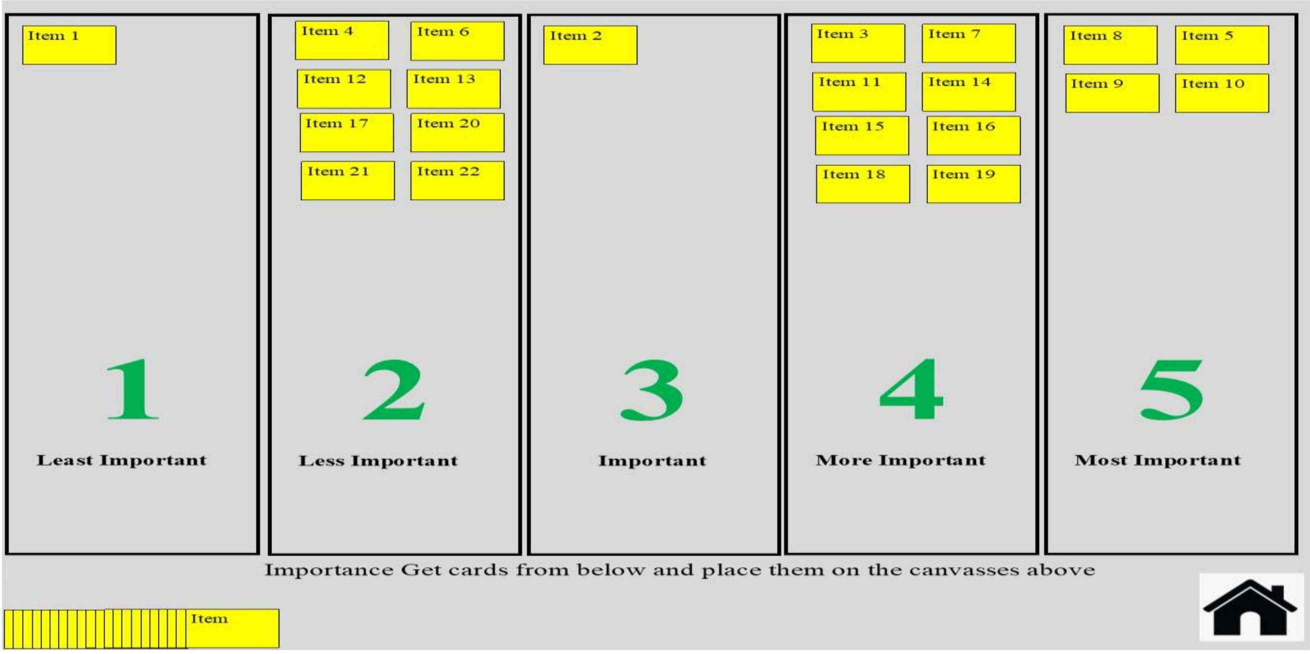

Figure 3: Moving cards from the deck to one of five vertical columns

Step 3: Moving cards from one column to another

If we think that we have placed a card into the wrong column, we can drag the card from one column to another column.

For example we can drag the card "item 22"(figure 4) from column 2 'less important' (picture above) to column 5 "more important" (figure 5).

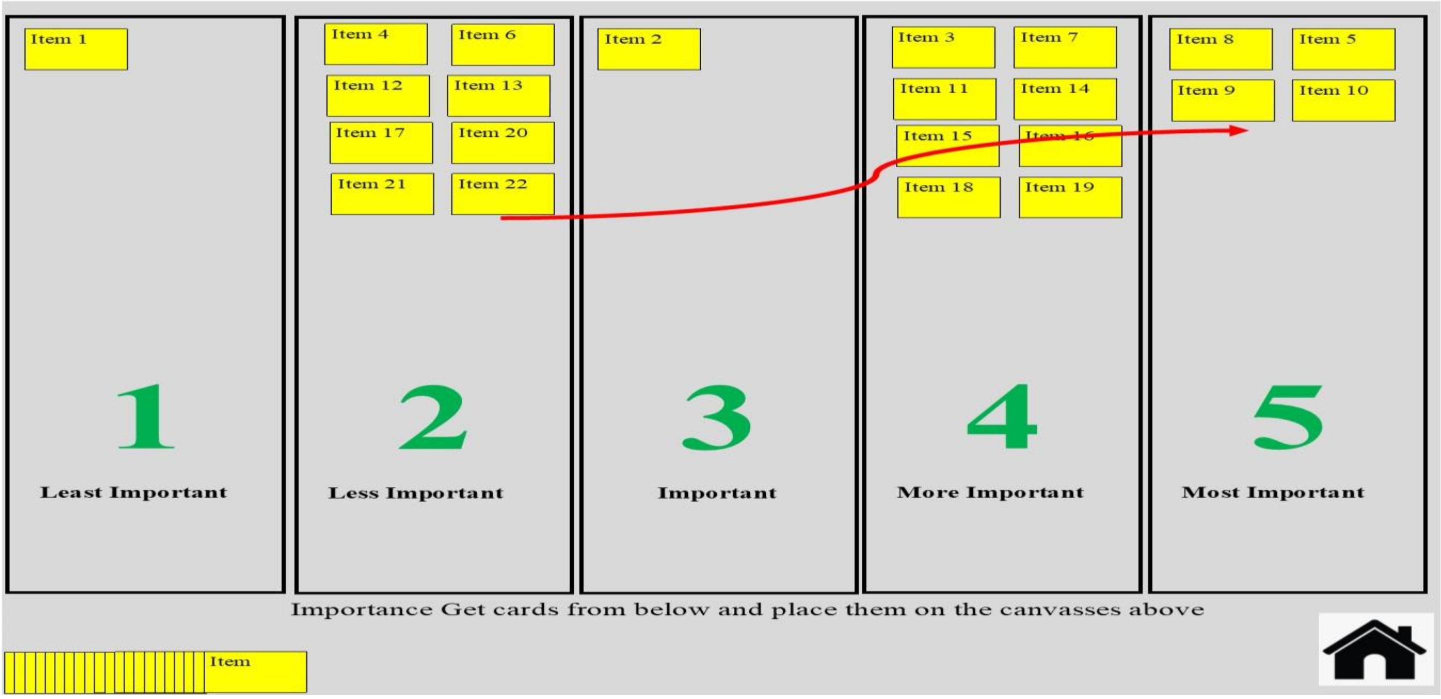

Figure 4: Moving statements between vertical columns

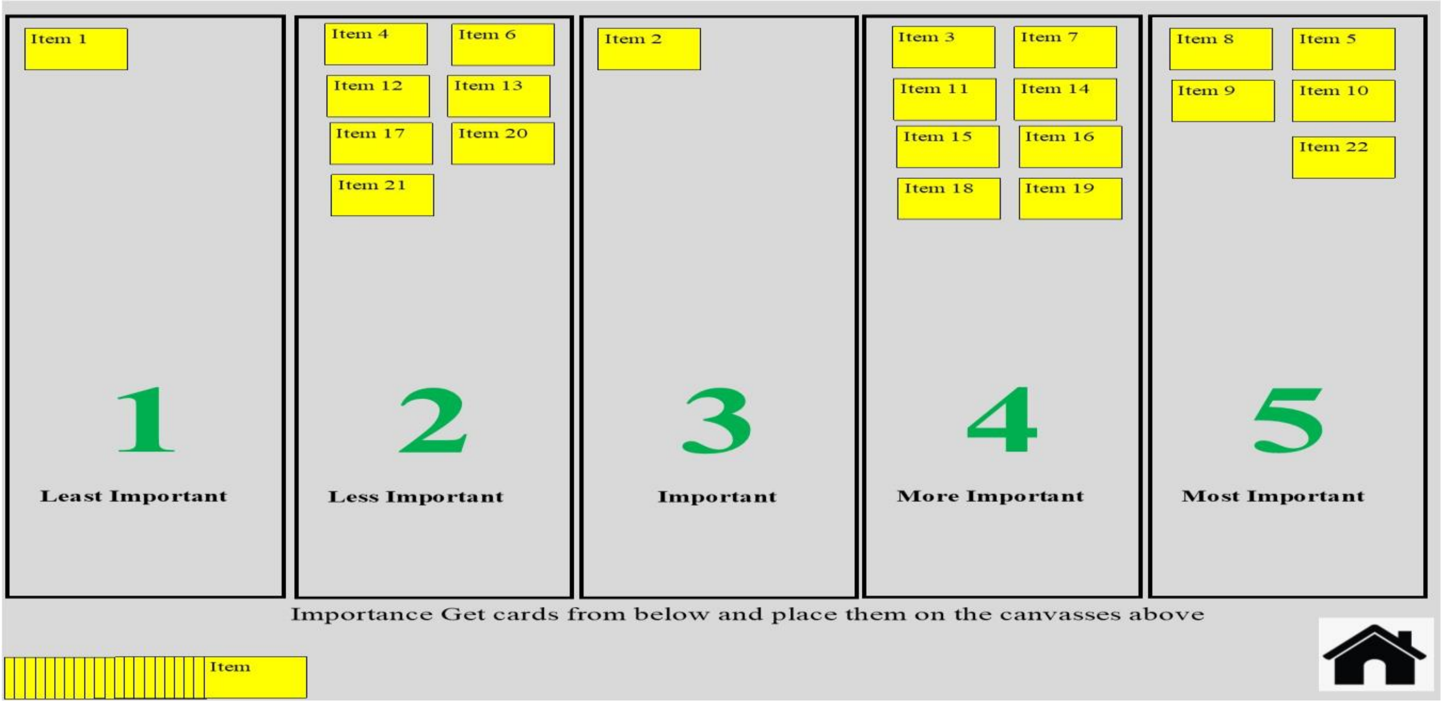

Figure 5: Moving statements between columns

Step 4: Ensuring that each column has equal number of cards

We need to ensure that equal number of cards are placed in each column. If the number of cards in one column exceeds the maximum number of cards that it can have, it will not be saved and these cards are placed back into the deck (at the bottom left corner where all cards are seen at the beginning).
For example, in figure 6 “item 78” exceeds beyond the capacity of column 4. If we have to place the card in this place, we don’t have any more space here; so we need to create space for column 4.

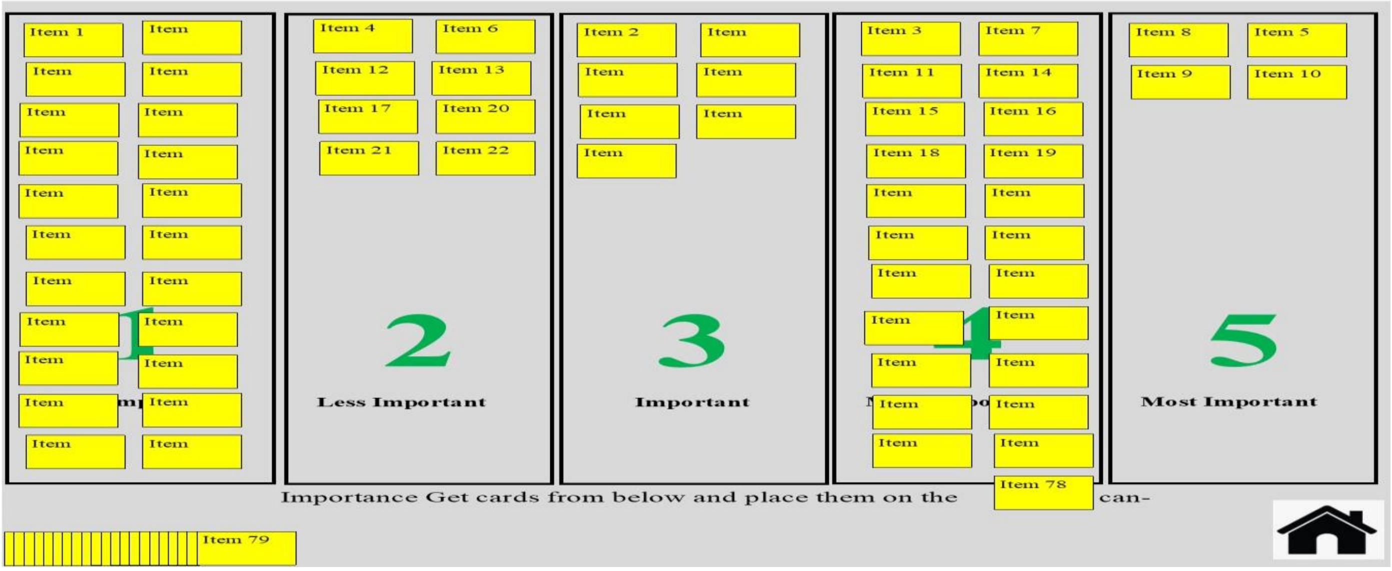

Figure 6: Managing excess cards in one column

For this, we need to remove a card from this column. We can do it by selecting a card (say for example “item 11”) which we think might not be suitable for this column and placing it back to the deck. The card “item 11” needs then to be organized into one of the four remaining columns before we can proceed further.
We need to continue till we complete placing all the cards from the deck into one of the five columns.

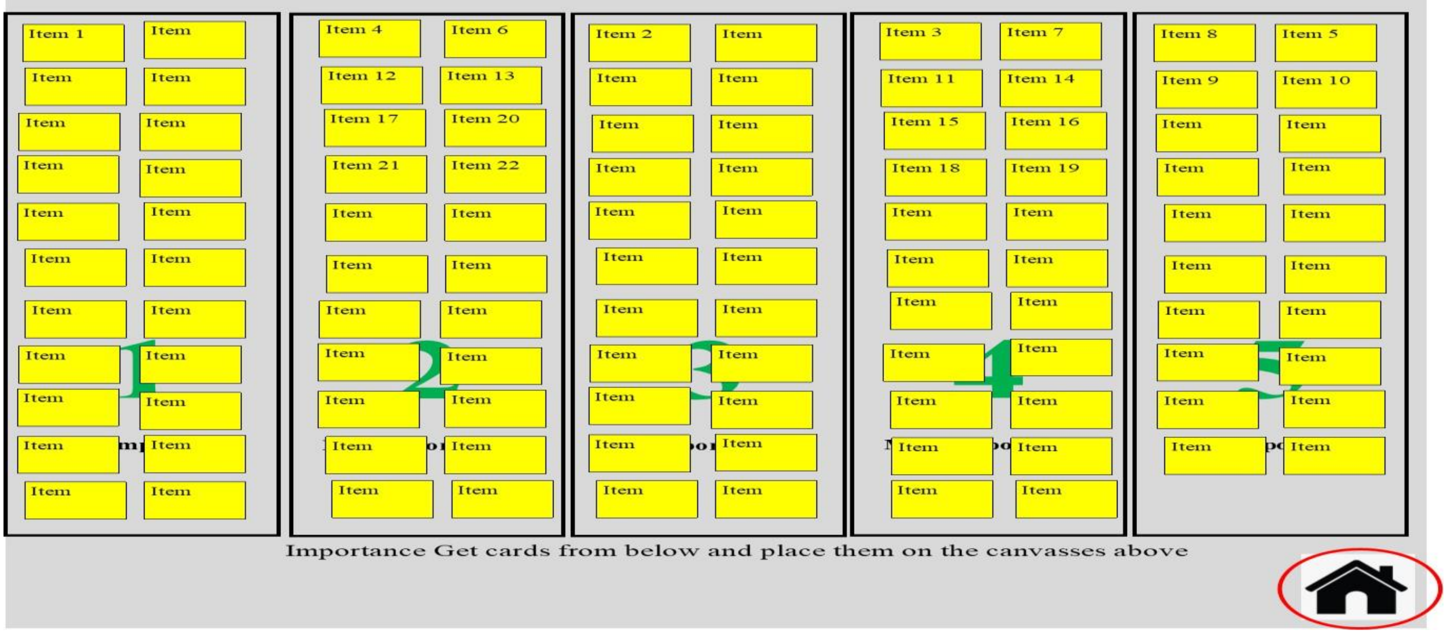

Figure 7: Prioritization task home screen after completion

We need to click the 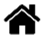 botton to return to the main home page (Figure 1).

Task 2 Clustering

For this task, we need to group the statements, based on their similarity. We need to place the statements into if one group if we think they are related to each other. We can create as many groups as we would like. We can use the individual statement only once. After we complete arranging the statements into individual groups, we need to provide a suitable name for the individual group.

Step 5: Navigating to the clustering task

After completing the prioritization task, we need to click the home button. It will take us to the "Ariadne" home page. Now please click on clustering (figure 1).

Once we click the clustering task, the following screen appears in our computer.

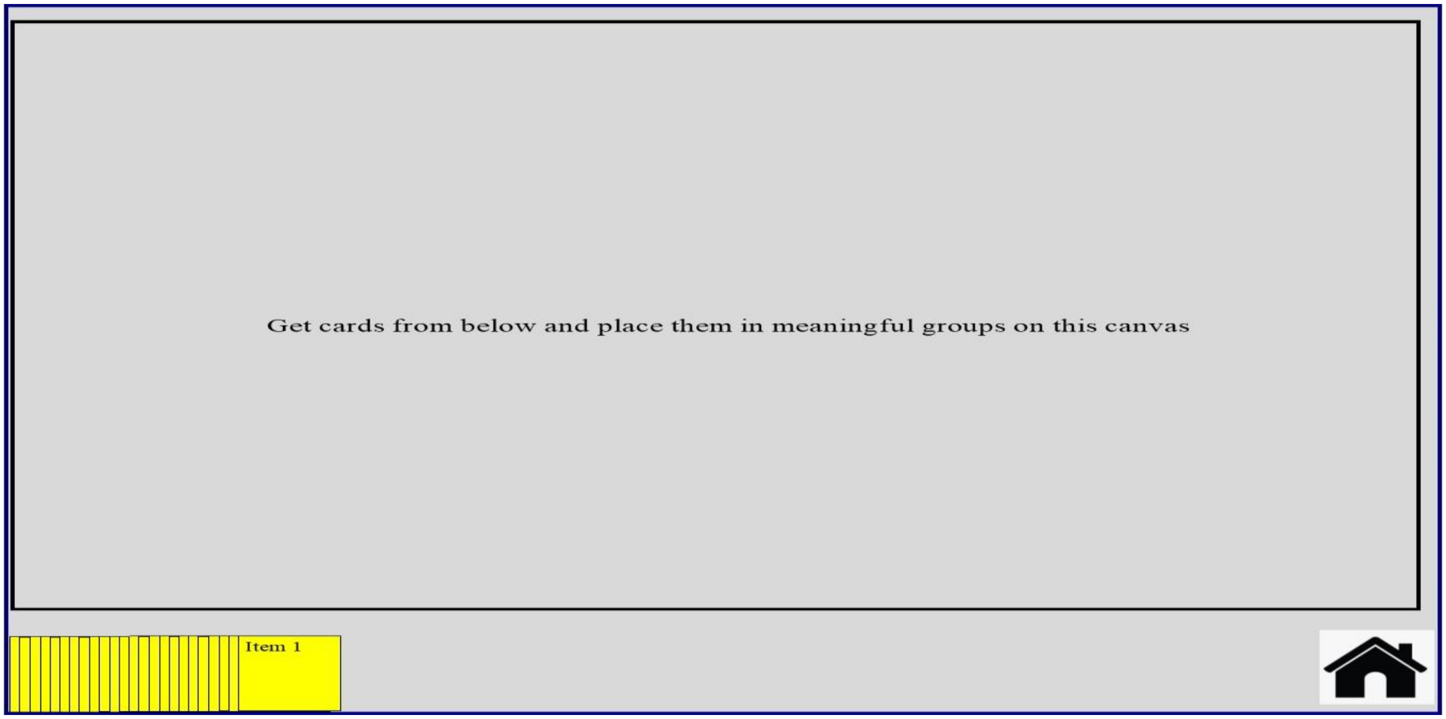

Figure 8: clustering task home page

Step 6: Putting cards into clusters

We can drag cards from the deck (right bottom corner of the screen) to the canvas above and place the cards into clusters. Here, we need to note that we can place the cards at any part of the upper canvas.

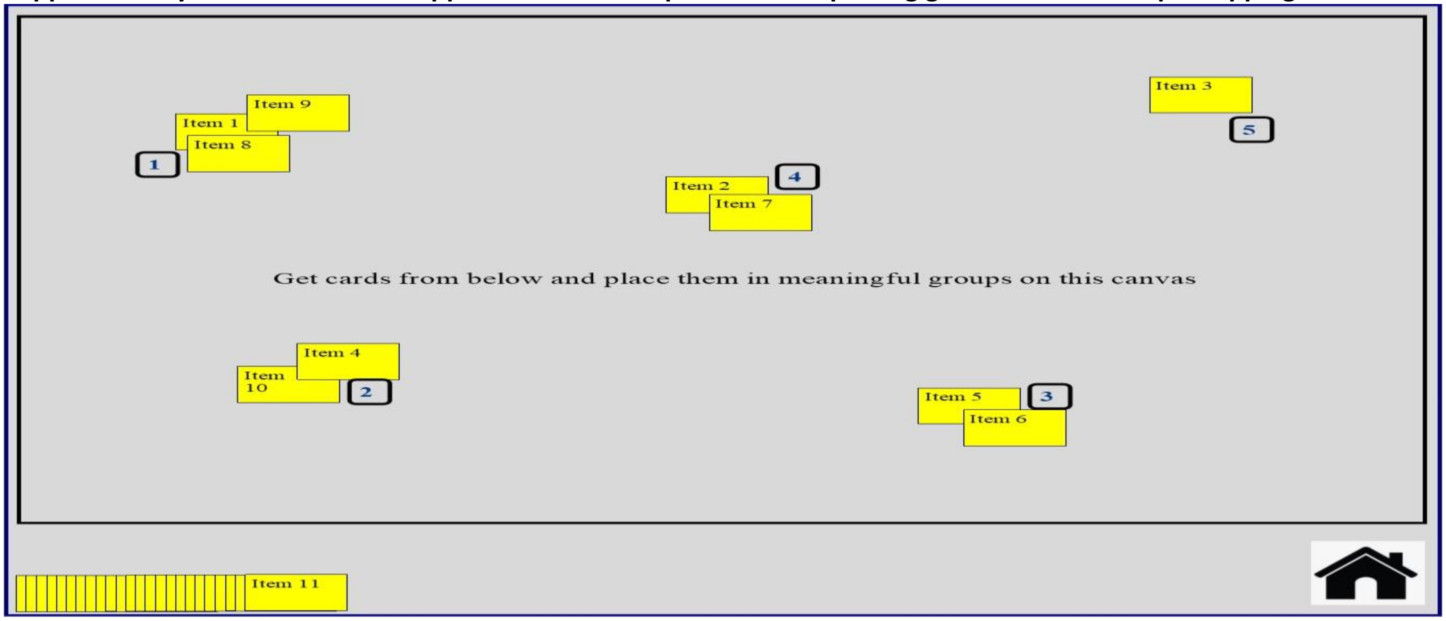

Figure 9: Moving the cards from the deck for clustering

In figure 9, we can see that the first ten cards have been moved from the deck and arranged into five clusters at different parts of the canvas. Ariadne automatically names the cluster as number. For example, the five clusters created in figure 10 have been named as 1-5.

We need to carry out this step till we complete all the cards from the deck.

Step 7 Renaming the clusters.

Once we complete the tasks, we need to provide a meaningful name to the cluster that can represent the statements in the cluster.

For naming the cluster, we need to click on the number in the cluster, and type in the name.

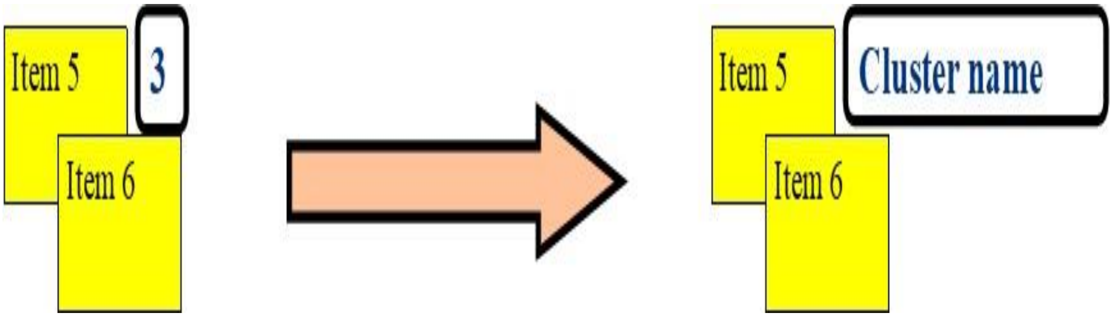

Figure 10: Renaming the clusters

For example, the group 3 has been renamed as “cluster name”

Step 8 Saving the clustering task

Data will be automatically saved on a real time basis.
