# Supplementary material for: Development of a Reporting Guideline for Trochim’s Concept Mapping
Source: Methods Protoc. 2025 Mar 3;8(2):24. doi: 10.3390/mps8020024 (PMC11932253; doi:10.3390/mps8020024)
Supplement: Supplementary file 1 [file mps-08-00024-s001.zip › Supplementary document 5, List of statements generated from brainstorming.pdf]

1. There needs to be some details about the method
2. There needs to be some details about the approach
3. Stating the dates when the study was conducted
4. Information on how the study was conducted (Online totally, face to face data collection, interviews used initially for brainstorming…all sort of things)
5. Study approach needs to be clear
6. Clarification as to what the focus prompt is (so that is fully understood that the approach used is relevant to the focus prompt and for the participant groups)
7. Sometimes it is important to offer a figure of the research process
8. Description of the setting/context of the different phases of concept mapping process
9. Details on each of the activities (for example brainstorming)
10. Who the participants were?
11. Information on the number of participants at different stages of concept mapping (who answered the questions, brainstorming activities and so forth)
12. Some description around their demographics depending on what we are asking (for example in research with students, we need to understand the language skills of the participants)
13. The role of the facilitator in the process should be explained
14. Information on how concept mapping software is being used
15. Clearly talking about the four steps to analysis and whether there was any movement away from the traditional four steps
16. Making sure that the data cleaning (and acceptance) process is in place
17. A description of the data cleaning process
18. Tables to show people the demographics
19. The original (raw) statements need to be provided
20. List the full statements in the appendix
21. Information about the ratings of the clusters
22. Examples of the statements to demonstrate the individual clusters (for example four of the statements and of all statements in the appendix, in the table)
23. Report on the Stress value (and the acceptable range so that a person reading for the first time understands if it is within the range)
24. Description of the map and what the points represent
25. Relationship of the cluster and statements
26. Talk about how the statements are distributed within the clusters
27. Description of the cluster labels (the actual description of the map, the clusters and numbers and the name them and provide the figure of the cluster maps with labels and the statements as points)
28. Give an understanding of what axis means
29. Provide cluster characteristics (a table of cluster characteristics)
30. Report point and cluster map
31. Report on each of the cluster rating map (if used more than one rating scale)
32. Depending on the type of GCM and what we do next, we may need to report a go-zone and what it is for
33. Ladder graph depends on what we want to illustrate from the piece of work (some of the reports are optional)
34. If go-zone is reported, a figure displaying the statements in relation to two rating scales (can be overarching or individual go-zones; examples and total number of statements from each of the quadrants)
35. Top five most important statements in the two rating scales
36. In describing a ladder graph, a figure with description to support the figure themselves
37. Increasing transparency is about engaging the right people
38. Provide the references of all the papers we included
39. Demonstration of how raw statements changed during the reporting process
40. Role and engagement of the facilitator and the advisory team is important
41. Information on the data analysis process (who the people were with when we went through the analysis process)
42. Explaining the process of preparation (to enable people to understand and be able to provide a valuable contribution)
43. There needs to be an introduction about the grant of the study
44. Some information about ethical approval for the study (whether ethical approval was sought…and who was that sought from… the committee and committee dates when approval was given…any identification code given to the ethical approval)
45. We need to explain the aim of the study
46. We need to explain the purpose of the study
47. Information on the focus of the study

48. Information about the aims/objectives of the concept mapping study
49. We need to explain why we need to adapt the concept mapping approach in our study
50. Information to justify why concept mapping approach was the most suitable one for the study
51. Need to provide information about the target participants involved in the study
52. We need to illustrate the preparation information in the report
53. Generate a general question (focus prompt) to ask the participants
54. We need to include the steps adapted in the study
55. Details of what was conducted (in each of the six steps of) concept mapping
56. We need to provide information about the number of participants in each stakeholder group
57. We need to provide information about contribution/engagement of the participants for different steps in concept mapping
58. Information about the number of statements generated from the participants
59. Information of how the statements were handled (were they returned to all the participants or did we recruit a separate group of participants to rate the statements)
60. Information on statements returned to the participants for clarification/validity
61. If the statements are returned to the participants for validation, we should note which group was involved in the validation of the statements
62. Information on what procedure was adopted to sort the statements by the participants
63. How authors arranged the sorting of statements
64. Illustrate the rating/importance that we used (need to explain which Likert scale we used) Information about analysis we conduct to represent statements into a map
65. We need to report which method was adopted to analyse data
66. Details of the procedure to name the clusters
67. Present a description of the concept map generated (in the study)
68. We need to present the final product of the concept mapping research
69. Need to use the concept map to generate further product to achieve the aims of the study (for example instrument/protocols)
70. A clear information on the type of maps generated and presented in the report (it may be possible that we integrate a few maps together and present the final map)
71. Present a flow of participants in the study
72. Present a flow steps followed in the study
73. It is important to have a clear description of how the concept mapping process is done
74. Should provide information on the research question
75. Background should give a clear explanation of why concept mapping is the right solution for answering the research question.
76. We should clearly explain that our research goal fits the methods
77. Clear explanation or description on how you exactly set up/organize these different stakeholders
78. Some information on how representative our target population is
79. Information should be provided on the process (steps) of concept mapping
80. Explanation of how we combined the ideas
81. It is important to mention on which scales, you rate the ideas
82. Who and how many people decided the interpretation process is also important?
83. It is important to be clear about how and analysed the contents to create a concept map
84. It is important to clearly describe the process of how we came up with the best solution for the number of clusters
85. Further analysis of the content within the cluster is important to identify the pattern in the data
86. Elaborate the process of naming the clusters by the target population, or whether you did it yourself.
87. If you could get an explanation whether you interpret the results correctly.
88. Any additional calculation should also be really reported
89. it would be great to have all ideas belonging to each class in clusters as an additional file
90. Translation to English if study is carried out on a different language
91. It is important to be able to go back to all individual ideas belonging to the different clusters.
92. It is okay to provide information on a group level for the studies
93. In the discussion, we address the results from our concept map
94. It is important to include the ethical aspects of the study
95. A clear description of the methodology
96. We should report the methodology
97. All the individual steps should be reported.
98. Information on who develops the focus prompt

99. The development of the focus pump is important
100. Involvement of participants in the development of the focus prompt should be documented
101. Reporting the individual steps depends on the audience
102. It is important to describe the participants that we work with
103. Information on how you identified the participants
104. Information on participants across the different phases (whether same or different)
105. Information on how much detailing was required on the brainstorming session to get out the responses
106. We need to pay attention to the how data is analysed
107. Description of how we came up with the rating scales
108. The sorting of the data needs to be described very well
109. Information on how card sorting task was conducted (online or with like clue cards)
110. The main concept map should be described.
111. Description of other concept maps that had could have been generated under the data
112. Justification of why one concept map was chosen over the other
113. Description of how cluster names were labelled
114. It is also important to have some guidelines around on how to determine cluster names
115. Having a pattern match and at least a go-zone along with the main concept map is desired
116. Information on what is the limitations purely based on the methodology
117. Information on (contextual) basic demographic information collected during the study
118. Putting it into context of the study
119. It is important that the actual focus prompt is reported
120. Description of how we selected your sample
121. Description of how why you selected the people/stakeholders
122. A discussion to address the issue of sample size
123. Some information about the participants in the various spaces (number of people in focus groups and number of people who did the sorting/rating)
124. Basic demographic information about participants whatever may be relevant
125. Information about whether the same people participated in the various steps (if yes, how many)
126. Mention the number of statements made during the brainstorming sessions
127. Mention the number of statements we use for the rest of the process
128. Report on the rating (either for individual items/statements or at the cluster level)
129. If we use more than one rating, we need to report the correlation between the two ratings
130. Note of how we have instructed the participants during brainstorming
131. What instruction was given to the participants when they did the sorting/rating task
132. Some detailed information on the actual analysis
133. Map needs to be presented both with and without the clusters
134. To report the stress index/value that relates to the multidimensional scaling
135. It should also be clear to what extent participants were involved in interpretation of the maps, (labelling the clusters, and the higher order interpretation and so on)
136. Report on the breaching values which we get both for individual statements and the clusters
137. Need to report something about the sort of Integrity of the clusters
138. If possible, it's valuable to do report higher order interpretation of the map (like you have several clusters and you see like those who are in a certain region of the map they seem to belong together represent something)
139. Further accounts in like in the discussion section on future implications (where do we go from here? What do we do with this?)
140. Need to have a clear goal and aim of what authors are trying to achieve.
141. There should be a clear explanation of what is the purpose of the concept mapping (for local, policy development, develop a measure, develop a mission)
142. There needs to be something around the status of the participants/actors.
143. Some narrative about how participants are approached, recruited, and included.
144. A note on what participants were asked to do item generation stage
145. It is important to know what the instructions for participants were during the item generation stage
146. A focused group research would always try to be transparent about the power dynamics that potentially influence what's going on
147. A simple sort of factual condensing of a lot of that information (brainstorming)
148. A note on how the card sorting task was done (paper based, online, use of software)
149. Information on how much support people needed to complete the card sorting task (It is worth as reflecting where your work well - it can be a factor within the analysis, that if participants are helped by researchers or family members,

it may influence the way sorting exercise is completed, for example – older people tend to group things in bigger group but if supported can come up with finer distinctions within the card sorting)

150. We need to report how statistics (multi-dimensional scaling) leads to the software to produce stuff we desire.
151. Information about who was involved in interpreting the clusters
152. Any sensitivity analysis (correlational matrix) that needs to be considered?
153. Did you do any sensitivity analysis?
154. Syntax (code) for data analysis should be provided so that people can go through the data and reproduce the map
155. An online appendix
156. Clear idea of how we are going to use the concept mapping.
157. Is there a standard way of reporting items that group in the middle and are conceptually no where in any of the clusters?
158. Obvious things around socio demographics.
159. We need to be able to understand the nuts and bolts of what's going on behind the scenes
160. We need to report on the group that is doing the planning (Is it researchers, academics, or stakeholders; and obviously, it's best to have a group that's composed of all of these)
161. Information on how we came up with our focus prompt
162. A description of the preparation, planning phases.
163. The demographic information of the participant/stakeholder groups (will be different for each study - depends on what we need to include in our study)
164. It is important for to report the exact methods that are used to understand what some of the concepts mean
165. Information on the statements added by the research team
166. Include what your indicators are (feasibility, importance etc.)
167. Information on the program we use
168. Justification on the program we use
169. A description on the preparation on the actual software (for example – translation of language)
170. We need to report the differences between different stakeholder groups
171. Need to report the study design
172. Having a rationale for why concept mapping it was the chosen method
173. How you identified the topic
174. How stakeholders were identified (who were the stakeholders)
175. How stakeholders were included throughout all the different steps
176. Need to describe the context and the setting
177. Need to describe how participants were recruited (helps people know how inclusive or exclusive you were in your study and in your recruitment)
178. Each step of the process is clearly described (in terms of what people did and what happens through each of the steps)
179. How ideas were generated
180. The number of unique ideas that are sorted should be included
181. Need to report the rating scale
182. Need to justify the rationale behind the rating scale
183. How those ideas were sorted
184. It is important to talk about who participated in for each of these different steps
185. Need to report the process of deciding on the concept map from sorting data
186. Need to report how the data was analysed and represented
187. We need to report on the statistical analysis
188. We need to report the program used for statistical analysis.
189. Note on who participated in deciding the concept map (understanding who is making those decisions is important)
190. We need to justify why stakeholder group were not involved in interpreting the map (the other thing is that if there if there's a smaller group interpreting the maps, that doesn't include all this initial participants or stakeholder groups, that should be clearly stated about why they were not a part of the group that interprets the map)
191. The number of clusters that are chosen in the concept map needs to be reported
192. Stress value needs to be included (so that people have an idea of just how well the sorting went right or the idea is too diverse)
193. And then including the concept map itself, or its what's included the results
194. It is important to report quite transparently the five different stages of the concept mapping (ideas generation, statement reduction, sorting, rating, and analysis and interpretation)
195. We need to be as transparent as possible around those five broad areas.
196. It is important to report all kind of standard areas around your target sample
197. How did you identify those participant groups to take part in the concept mapping?

198. And how did you approach the participants?
199. Out of the people that you approached, who (how many) decided to take part?
200. How have you recruited participants?
201. Who you've selected to take part in the concept mapping?
202. How brainstorming information was obtained (I think group concept mapping can be delivered in lots of different ways. It can be done completely as an asynchronous activity where everybody participates in their own time on a one-to-one basis or as a group activity)
203. How was the card sorting activities undertaken (online or using pen and paper)
204. It is important to report who took part in each of the stages
205. It is important to report what impact different groups of participants might have on the data (for example, if the sorting data was primarily undertaken by a particular stakeholder group like staff, how might that affect the concept maps that you then go on to produce)
206. Need to report on the stress value and the acceptable range
207. Need to report characteristics of participants in different phases (they may be same or different)
208. It is important to have limitations on methodological issues.
209. It is important to get a balance between reporting issues that might impact on data quality (for example, participant groups, more practical issues around how the data is collected - collected in person or remotely; did the participants fill it in directly by themselves or was it done as a pen and paper activity and then uploaded by researchers)
210. How was the sample recruited?
211. How were people selected?
212. The characteristics of the of the participants
213. What was the response rates?
214. Was there any warm-up statement?
215. What was the statement for brainstorming?
216. How was brainstorming session conducted (face to face or online or using software)?
217. How many brainstorming sessions were conducted?
218. How long were the brainstorming sessions?
219. Who were present in the brainstorming sessions?
220. The number of ideas generated,
221. Did the participants get any incentives for joining?
222. What steps were done in which step?
223. Who did the analysis?
224. How was the number of clusters decided?
225. The number of clusters generated from the statistical analysis
226. Was the clusters shown to the participants to ask if it represented their ideas?
227. Were the items reorganized after the clustering?
228. What were the criteria used to reorganize items after clustering was done? Was it checked with the participants?
229. The number of clusters produced after the researchers may have reshuffled things
230. The double checking of all in the beginning needs to be reported
231. Information on what things went differently than planned initially (drop out, disturbance during meetings)
232. Everything that happens in the data collection need to be reported
233. Information on participant flow in the study (number of participants that were interested, participated, or dropped out)
234. Reasons for dropping out by the participants
235. Explanation of how the concept mapping as a methodology has added value to the study
236. It is important to understand what people have done to prepare to use this methodology.
237. Enough attention to the language is needed
238. Enough attention to the syntax is needed
239. Methodology should explain how decisions were made
240. Methodology should explain what the process was
241. We need to report the issues involved in the concept mapping process
242. Any issues on language translation is to be reported
243. The procedural part of how people worked through selecting of the clusters (the problems as well as the good stuff)
244. The actual experience of concept mapping process should be talked in the discussion
245. We need to report the limitations of the study
246. Providing a simple setting for brainstorming would be better for the participants
247. Simplicity with something how maybe something more tangible for participants is important
248. Details on how sorting was carried out
249. Information on how data was entered

250. We should explain about how we tackle issues during our data collection
251. Abstract/title should contain concept mapping as a methodology
252. Abstract should contain basic information about what we found
253. Introduction should explain why we choose the method
254. Justification for choosing concept mapping as a method
255. Report should contain explanation on the method
256. Aim of the study
257. Explain the steps of concept mapping and provide references for that
258. Development of focus question – either in the introduction or in methods
259. How we came to the focus (research) question should be explained
260. How the focus question was developed
261. Why did we choose the specific question?
262. Relevance of the focus question to real life (or the topic) we are researching
263. Number of stakeholder groups
264. Justification for the choice of all stakeholder groups
265. Participant selection process - How stakeholder groups were chosen
266. Need to explain how many participants we had and who they were
267. Details on all phases of data collection process (brainstorming, prioritization, and clustering)
268. Mention and explain key steps of methodology
269. Any changes to the methodology should be explained with references
270. Justification of the process for item gathering (brainstorming) session
271. Method of statement reduction and finalization (duplicates and how we put statements together into concepts)
272. How we ensure that concepts represent the statements properly (wording cannot always be same…what we do…how many people are involved in that process)
273. Any use of software for reducing the statements
274. Explanation of any deviation from the protocol or to the process of concept mapping should be mentioned and justified
275. If any more steps are added, they need to be reported along with any decisions that led to it
276. Any other decisions (or changes) made throughout the study should be reported
277. Explanation of methods to participants (questionnaire about how they understand it …may be a good idea or may be not; explanation of data analysis)
278. Need to explain the participants of how we analyse prioritization and clustering task
279. To ensure that qualitative and quantitative parts are analysed according to the required standards (should be reported in standard relevant to those methods)
280. Properly explain the data analysis
281. Use of any data analysis software
282. Importance ratings of items and clusters
283. Items identified
284. Clusters identified
285. How we came across the clusters
286. Concept map
287. Groups of participants
288. Note of any outstanding items that do not belong to any of the clusters and how we manage that in data analysis
289. Any comparisons based on the aim of the study
290. Participant number needs to be noted
291. Description of characteristics of each stakeholder groups with basic demographic information
292. If we are comparing the views of different stakeholder groups, we should report information separately (information about stakeholders' understanding of the topic)
293. What are the differences among the different stakeholder groups?
294. Map could help creation of meta-clusters
295. We may need to report the superclusters in the discussion (if there are too many clusters)
296. Discussion mostly depends on the results.
297. Any points in discussion should be included in the results section
298. Main axis of the concept map should be named
299. Two-dimensional concept map should be reported along with its axis
300. Should contain a concept map with visualization of all the concepts
301. Main themes around the statements and clusters should be named.
302. Utilization may depend on the study aims
303. Sometimes it is not obvious to report utilization of the maps

304. Ethics needs to be reported
305. If there are sensitive groups, there can be different ethical approaches.
306. Proper reference to anything used
307. the very important thing is to introduce your concept mapping
308. the very important thing is to justify why you're using the concept mapping
309. We need to be clear about why we are using concept mapping process and why this is going to be beneficial for our study.
310. Talk about the focus
311. Detail about the study population and the setting
312. We need to justify our participant related to the study
313. Need to justify how our stakeholder group can be beneficial for our study
314. talk about inclusion and exclusion criteria for each group
315. How many are going to participate in the study?
316. How we are going to recruit them
317. Note on what demographic data we are going to collect.
318. How do we collect their demographic data?
319. A timeframe of how long to complete the individual stages (collect the data)
320. We need to report the limitations around the time frame between two phases (Doing two phases together can create bias - if you do those two processes too close together, there is a risk of the participants remembering what they've said in the statements; the people who done it last are quicker at prioritizing and clustering because they remembered what they've said, and that was important to them)
321. Tell the number of ways people could communicate for the brainstorming (How we are going to do it -whether it's going to be focus group, email, or via telephone)
322. Talk about segregating those focus groups (mixing participant groups may contribute one group influencing another group)
323. Talk about how you are going to run those specific focus groups (having more than a certain number may impact people’s ability to have a voice and to be able to contribute to the focus groups)
324. We need to be specific about how we are going to collect the data (because with the original concept mapping, they focus very heavily on focus groups and getting people together but more current literature talks about different ways of collecting this information)
325. how we are going to determine if a statement was a quality
326. We need to justify how we are going to reduce our statements.
327. Talk about structuring the statements
328. Talk about how participants could undertake card sorting tasks (on a computer or in person)
329. Information given to the participants for clustering and prioritization
330. Information about the content of the program we are using
331. Mathematics behind the program we are using
332. how do you find the best map?
333. Talk about how we are going to get consent from our participants (also outline the withdrawal of consent and how to manage that).
334. Information on the ethical considerations on the study.
335. We need to justify under each one of those six stages, why we are doing that to make that stage
336. We need be describing how our program undertakes the analysis
337. We need be describing how our program generates the desired results
338. It is important to talk about the limitations of the concept mapping project process
339. There should be either group concept mapping or mixed methods in the title.
340. We need to explain why concept mapping approach is helpful in addressing this problem
341. Need to explain what is the problem or gap that we are trying to address
342. Rationale for choosing concept mapping to address the gap in the literature in the introduction
343. We need to include the focus (brainstorming) prompt
344. We need to explain how the target population/sample was defined
345. Who were the participants?
346. How were the participants selected?
347. Demographic information for the brainstorming participants.
348. We must be clear with the instructions provided to the participants during the process of concept mapping (can they make as many piles as they want? Are there any rules? Do you do you name the piles or do they name the piles)
349. It is important to talk about how brainstorming was conducted (remotely, or in-person)
350. It is important to talk whether any software was used for brainstorming

351. We need to report if there is any power dynamics playing during the brainstorming sessions.
352. We need to talk about the training of the researchers and to what extent do they have training in group concept mapping as well
353. How many items were there in the original pool?
354. Information on who was involved in the decision making for reducing the statements
355. We need to clarify how we whittle down the initial pool of statements from brainstorming
356. How many items were there in the final pool that you sorted?
357. Information on the Likert scale used for rating question
358. Report on the differences between participants for brainstorming versus card sorting tasks (they may be same or different or a subset of initial sample)
359. Information on who decides the cluster names (researchers' words/participants/both)
360. Information on the point map
361. Information on hierarchical cluster analysis
362. Information on multi-dimensional scaling
363. Information on how many models we reviewed
364. Information on how cluster names are decided
365. The titles of the clusters needs to be reported.
366. A table of all items with their respective clusters
367. Need to report the point map with explanation of how to interpret (what does it mean when the little dots are close together, versus when they're far apart)
368. Explaining multi-dimensional scaling
369. A table with information on the average rating score
370. If we have a go-zone plot, then we need to include in the table what quadrant each item falls
371. Need to report the cluster map with explanation of how to interpret
372. We need to include the stress value
373. Include the median stress value for concept mapping studies so that the reviewers can understand what that means
374. Report on ladder plot if we want to see comparison between the stakeholders
375. Explain how clusters are created by superimposition of point map
376. Need to explain how the piles translate into the point map
377. Explain how we decide the number of clusters that are selected
378. Explain why you chose one cluster solution over other cluster solutions
379. The decision-making process is crucial
380. We need to report how do we make those decisions (to reduce statements), on what things we let go of
381. If relevant, information on the role of the participants within the organization or their job titles, should be provided
382. Discussion on implications for future research
383. Discussion on how the concept map can this be helpful in day-to-day life.
384. Discussion on how the concept map fill a gap in the research
385. A background to explain what your problem is
386. A background to explain what the gap in research is
387. Aim of the study should be explained
388. A background to explain why the concept mapping methodology could help address gap in literature
389. We need to explain how the study has been designed with different stakeholders
390. It is important to describe the stakeholder groups/members
391. We need to explain why we chose our sample
392. A clear description of how many stakeholders were involved in the different stages of concept mapping.
393. We need to justify the inclusion or exclusion of different stakeholder groups
394. We need to report on the response rates from different stakeholder groups
395. Note on the dropouts
396. We need to report on the details of pilots to get our research prompt (If pilots had been performed)
397. We need to describe how the participants were engaged (and why; if not, why not)
398. We need to be clear on different stakeholder groups included in each stage
399. We need to properly describe the different steps that we are undertaking
400. Data collection process needs to be clear.
401. Information on the quality measures taken during the study
402. We need to have full insight in how the process was performed.
403. We need to report what the results are in the different stages of concept mapping.
404. A flowchart of the methodology is good to visualize the concept mapping process (that's a personal preference, and maybe that should be optional)

405. Discussion should include results from your research questions
406. Discussion should include a reflection on the methodology (if it worked, or if it didn't work)
407. We need to report on the software we are using
408. We need to report the codes used for the data analysis
409. We need to report which software we used for sorting of the data (software - optimal sought or optimal workshop – explore more about it)
410. We need to be clear about the different stages
411. We need to justify why we are not using certain steps in the concept mapping process
412. We need to report on the objectives of the research question
413. The purpose of why concept mapping is the methodology is an approach of choice.
414. Define the target population to answer the research question
415. Need to report how people are recruited.
416. Explain how the brainstorming activities were conducted (synchronous/asynchronous, use of software, in-person or virtually
417. Explain how the card sorting sessions were conducted (in-person or virtually)
418. We need to provide a reflection to the concept mapping process (steps of statements synthesis session, like exercise and activity - how you are condensing statements in a way that is then translatable to the next stage for sorting and reading)
419. Demographic information is contingent on what is relevant/important for the research question
420. We should report the detailed process of how concept map was selected (the decision-making process should be unpacked)
421. I think it is important to report on ethics
422. The abstract should always explicitly mention that it's a concept mapping study
423. The initial question or prompt used in the study should be clearly (explicitly) defined.
424. Some background information on the participants is important
425. The relevance of the question that's being answered to concept mapping is clearly indicated.
426. It is important that it is sufficiently clear why concept mapping is the best method to use to answer the question
427. Describe criteria used to filter the initial set of statements
428. It's important to provide detailed information on who did the filtering.
429. Describe how many statements were discarded in the filtering phase.
430. It is important to note how the prioritizing was done.
431. Information on the scale used to prioritize the statements (number of Likert scale).
432. It is important to indicate how many clusters of statements every individual created.
433. It is important to report on the specific statistical procedures used
434. A note on the use of software
435. The type of cluster analysis used needs to be reported
436. It is important to report on the process of selecting the final number of clusters
437. We need to describe the procedure used to derive at the final concept map
438. It is important that a systematic approach is considered for interpretation of cluster analysis (map)
439. Need to report on the degree in which the individual concept solutions accurately represent the final concept map (stress value or Ryan's square value)
440. We need to report the interpretation of the meaning of the clusters
441. Mention how these cluster labels are produced
442. It is important to explain how the axis were labelled
443. It is important to report on who does the interpreting
444. It is important to report on how interpretation was done
445. The criteria used for interpretation should be mentioned
446. All those sorting solutions should somehow be made available (as an appendix or an additional document or something like that)
447. It should be clearly indicated that what someone did was, in fact, group concept mapping, or at least the kind of thing we're talking about right now.
448. Researchers are precise in reporting (with justification of the choice) how they conducted different steps of concept mapping
449. Figures also are important pieces of information.
450. It is important that researchers mention how the report the results are going to be used.
451. Concept mapping study could be done without a direct application attached to it
452. The degree of representation (the quality of how well the final concept map represents what individuals came up with in the earlier steps of the concept mapping procedure).

453. Researchers report on how the different decisions were made throughout the process
454. It is important that researchers demonstrate some awareness of the regions of concept mapping of strengths and its weakest point
455. If possible, concept mapping data should be made available
456. Contextualization to the type of methodology
457. We need to show the different components of it.
458. There needs to be a definition of the concepts
459. An idea of the link between the different concepts
460. We need to describe how we did it (need to describe the framework that we used)
461. We need to mention what were the outcomes based on the methodology.
462. We should have an overview of the results per stage
463. We need to discuss the results with focus on the preceding outcomes
464. A general conversation to indicate the main findings for the stages
465. A general conversation to indicate the concepts that came out as a figure.
466. Information on the concept is that you will be mapping.
467. Information on who participated in the process.
468. Note on any tools that we used
469. Description of the process for data collection
470. How was analysis done?
471. We would need to justify every single thing around the decision that we make.
472. Information on how credibility, trustworthiness was applied in the interviews conducted during the study
473. A clear research question is needed
474. It is important to get exactly the right question the right nuances, in the right boundaries
475. The research question should be written out in the manuscript
476. Explanation of why we chose concept mapping for this study
477. Every concept mapping study needs to explain concept mapping in the methodology
478. Justification of why we selected our participants
479. We need to know how we selected our participants (to ensure participants are recruited in a fitting way)
480. Description of how our sampling was done (to ensure a good sampling of the target population)
481. You need to describe the steps that you took in the concept mapping methodology.
482. Explanation of how decisions on different steps were taken should be detailed (whether you took them alone, used guidelines, co-authors, or independent peers to make that decision with you)
483. Provide information on raw statements
484. A description of criteria on how statements were reduced (was it one set research alone, were there independent co-author who will assess that and their level of cooperation)
485. Explanation of why you chose the specific scales for the rating task
486. Need to explain the data interpretation process (choices and considerations)
487. Diversity in the participants (demographic characteristics) shown usually in table
488. Description of participants for different stages of concept mapping
489. A detailed description of what the results of the study were
490. We need to show different stages of the maps
491. We need to show the considerations that we made for the maps
492. It is important to report on the stress value (to show whether the data you're basing your concept mapping your concept map on has actual integrity or whether the reliability is reasonable)
493. We need to justify the steps taken for the concept mapping process
494. We need to show how we implement mandatory steps
495. Report on the pilot testing of the focus prompt
496. It may be important to contrast concept mapping to other methods to answer that question
497. It is important to justify the use of concept mapping as a method to the research question
498. Note on whether everyone from brainstorming sessions participated in the subsequent step
499. If multiple sessions occurred with the same focus prompts, how responses were aggregated across the sessions
500. Description of how we assessed the saturation of the conceptual space (at what point do you stop doing sessions – how many sessions were required to get adequate sample size)
501. Why did you prefer brainstorming to an interview or other qualitative techniques?
502. The number of people conducting the (brainstorming) session
503. We need to detail on who are taking the taking those brainstorming sessions
504. We need to detail on how we have trained the person conducting those brainstorming sessions
505. We need to detail the process of how we conduct the interview

506. Information on whether the any sessions was recorded
507. We need to talk on the gap between participant generated brainstorming content with the sorting statements
508. The extent to which similar responses in brainstorming were combined or managed by the research team
509. Was sorting done for the statements generated from the individual session or on the aggregated statements across multiple sessions?
510. Information on how statements were consolidated after the brainstorming (separate, combined and consolidated across multiple sessions)
511. Information on changes made on the brainstorming material based on the recording
512. How were the ratings dimensions decided?
513. If in-person sorting is done - How many people needed help with the task (keep track of how many times the team sat down with the participants and helped them sort)?
514. The extent to which sorted material was managed or edited by the research team (like reworded)
515. The extent to which sorted material isn't verbatim from out of the participants mouth
516. The basis for exclusion of any sorts (sorting data)
517. The basis of decision to the final cluster solution
518. Whether or not final cluster solution decision was made in collaboration with participants from the original study
519. Information on what basis the cluster names were changed after participants decided with a cluster label (after generating it in collaboration with the original sample of people who generated the brainstorming material)
520. Information on decisions to remunerate the participants
521. The decision of how participants were engaged in data collection (online or in-person, alone or in groups)
522. Whether some assessment of literacy was made.
523. Demographics that would allow readers to generalize study results
524. Limitations of the database on how to use GCM as opposed to something else
525. Explanation to the comprehensiveness of the conceptual space that you've mapped.
526. Discussion on the generalizability of the findings (If you had another group different from the group you got - less educated, more educated, different race, different nationality, different country, whatever would they have said the same thing)
527. A table of the original brainstorm results
528. A table of the cluster
529. The cluster map should be presented
530. The point map should be presented
531. Stress value has to be reported
532. Bridging values for all clusters and statements need to be reported
533. How many potential solutions did you consider
534. Want some description of the theoretical framework used to understand the data we are collecting.
535. The paper has to describe its methodology
536. We have to publish our data set at the same time
537. Declaration of the source of funding
538. Declaration of any conflict of interest
539. We need to talk about the question
540. We need to talk about why they pick that question
541. We need to discuss what they hope to learn from this specific prompt
542. It is important to discuss how participants relate to the topic
543. It is important to report information on the participants of the study
544. We need to report if anybody influenced in selecting the participants (in one of the studies, the funder was bringing people that were tangential to the topic)
545. Information on how we made sure that those who participated in the study were the right people for the study
546. To me reporting on how many people participated
547. We need to report how brainstorming was done (in person or online)
548. Any language barrier should be reported
549. We need to report how people worked to categorize them
550. We need to succinctly describe all the different steps of concept mapping process
551. We need to describe how the process went
552. We need to explain the data analysis so that people can understand what it is all about.
553. We need to explain all the statements
554. We need to report if participants were engaged to describe the map
555. Explain how concept maps are formed from the scatter maps is important
556. Explain the different components of the maps

557. Need to provide an explanation of the areas under the map
558. We need to report if the researchers followed what they were doing
559. General description of how concept mapping is being used in the research (traditional way or participatory)
560. Rationale and details of how and why of the concept mapping study (how and why did they do, what they had done and what are the reasons of doing it)
561. Positioning of the methodology within the literature
562. Positionality of concept mapping in quality research
563. Justify why concept mapping is reliable methodology for the research question
564. Defining the focus prompt for concept mapping
565. Details of how focus prompt was developed/identified (was it researcher driven or identified by an advisory committee/community/stakeholders)
566. Information on pilot testing of the focus prompt (whether it was piloted; if yes, how)
567. Ensure that there is a mix of different types of stakeholders
568. Definition of community bounding (who stakeholders are, community/system of people whom we are focused on)
569. Definition of who is the stakeholder's system in the research is focused on
570. How do we ensure that we had enough/sufficient participation from different stakeholder group?
571. How sampling was conducted (snowball, maximum variation sampling)
572. Information on how people were reached out
573. Information on how many of the people responded?
574. Information on how responses were gathered (redcap, email, person, or combination)
575. Number of people who responded to the generation of ideas
576. Section to talk about the ideas generated from the study
577. Information on how we cleaned the ideas (how did we get to those 100 or less ideas) (research team, advisory board, or techniques – content analysis)
578. Number of people who participated in the sorting process (number approached, no of people responded, percentage responded, total number of responses received)
579. Section describing the structuring of the ideas
580. Information on how ideas were sorted (electronic card sorting or using actual index cards or both
581. Details about how ideas were rated
582. Number of people involved in structuring (rating and sorting)
583. Detail the process of concept mapping
584. Reporting by the specific steps followed in concept mapping (not all concept mapping studies follow all the six steps)
585. Did we give instructions at different phases of concept mapping process
586. Step 4 (representation) can be a part of data analysis
587. Describe what data analysis are (MDS and HCA)
588. Describing how multidimensional scaling was used
589. Describing how hierarchical cluster analysis was conducted
590. Need to present results by specific steps
591. Samples may be different in the different stages of the concept mapping
592. Results should show what we had done – ladder zone, go graph
593. Information on the stress value
594. Information of how scaling and cluster analysis was applied for example – point map, cluster analysis
595. Information of how cluster solution was chosen (did the research team choose it, if any mathematical algorithm was applied)
596. Any additional methodology/analysis if performed should me described (for example content analysis on top of sorting and rating ideas)
597. Justification/Explanation of do the findings of the research specifically answer the research question
598. Describe the research story (answer to three questions - how, why, and so what) in every single step of concept mapping
599. Interpretation and utilization have less place in typical journal articles
600. Interpretation should be under combined on how we came with the final concept map (how the final concept map was identified – whether determined by the research team or participant group or the advisory group)
601. Description of how the final map will be interpreted after the identification of the final cluster solution
602. Information on how the map is being used (may be even after the generation of the concept map. At the time of the manuscript, it may not have been used but would like to know how the map is planned to be used)
603. Only the demographic characteristics relevant to the research question should be reported (no need of irrelevant demographic characteristics that do not matter for the research question)
604. General demographics – age, gender to say that we have a diverse group of respondents

605. Demographic profile of participants in the different phases of the study (which step and what characteristics are relevant)
606. To ask the participants what their primary role within the project is
607. Need to have sections under the different steps
608. Information on working with an advisory group if involved
609. Purest approach – follow the exact steps in concept mapping
610. Applied/practical approach – every concept mapping study may not exactly be the same
611. Sections that add value to the research question should be detailed
612. Justification of why any steps were not followed (if any)
613. We should include the focus prompt question used for the study
614. Include whether any theory was used to derive the focus prompt
615. Report who was involved in developing the focus prompt (whether it was the research team or any community members)
616. If we did not follow one of the steps that should be mentioned in the methodology
617. Detailing how participants were involved in idea generation (focus group, nominal group technique)
618. Each of the methods used to generate the ideas should be carefully described (in-person or online)
619. We should also talk about how many sessions were held
620. How many people attended each session?
621. Who facilitated the group?
622. Was the person a concept mapping facilitator or trained in the methodology used to generate the ideas
623. How many items were generated during those sessions?
624. The average number of participants for each session
625. How many people attended, overall?
626. Description of participant demographics
627. How many statements were generated overall?
628. Details on the statement reduction process (Specific about how many statements for example, were deleted if they were redundant with other statements? How many were deleted, for example, if they weren't relevant to the focus prompt, so specifically underscore detailing, the generating the number of statement reduction process so that it is scientifically rigorous and reproducible)
629. What process was used by the research team to refine the statement set?
630. How many statements you ended with?
631. Information on number of statements added by the research team, if any? (if any of the members of the research team felt that there were gaps in the statement set)
632. Details on the origin of all your statements
633. Information on how structuring sessions were carried out (whether in person, or online)
634. Information on the instructions that are given to participants prior to engaging in the card sorting exercise (specially when done in person)
635. Information on the demographics of structuring sessions (the number of sessions held, number of participants attending the session, average number of participants in each session, average length of the session, average time perhaps taken for card sorting)
636. Note on any restrictions to card sorting process
637. Detailing on the rating process (number of rating questions, rating options used, number of participants completing rating)
638. Information on any additional demographic data
639. Information on the retention of participants across the different stages of concept mapping
640. Were new participants recruited for card sorting tasks?
641. talking about the minimum sample size to do the structuring to have a reliable structuring data
642. Using the checklist developed about reliability/validity of concept mapping (Using the article developed by Scott Rosas et al., as a guide for reporting some of those critical elements)
643. A description of how data is analysed by the concept mapping software (if used)
644. Information on how the clustering data is used in analyses
645. Information on how the rating data are used in analyses
646. Talking about the process of drawing the boundary (point map boundary)
647. We need to talk about how the final cluster solution was determined (Process of cluster solution reduction, and who was involved – research team, community members)
648. Information on why the how the clusters are positioned in the way that they are
649. The orientation of the cluster map and the cluster labels and the points.
650. Information on meaning of the clusters

651. Talk about the number of iterations for the concept map
652. Talk about clusters by label and thickness to show the relative importance of each cluster.
653. How were the clusters labelled?
654. Who was involved in labelling the clusters?
655. Give few examples of the cluster ranges data about the least and most important clusters
656. Providing examples of two or three highest rated statements within each cluster
657. Report on the stress value
658. A discussion on the meaning of the stress value
659. We should also describe any of the post hoc analyses used, and what those may mean as well.
660. Describe how the concept map will be utilized (any next steps that you'll use based on the content mapping findings)
661. Show how many people attended each step of the content mapping system
662. Information on any kind of post hoc analyses that you did (for example, if you're using a go-zone, or the ladder graph)
663. We can export some of the results and upload the raw data file
664. I think there might be supplementary files (with all statements, cluster maps with the cluster labels, the average rate cluster rating, the number of statements per rating, and all statements)
665. Describing whether your project is theory informed
666. We need to link the results back to either theory or conceptual framework
667. Describing whether someone has been trained in the concept mapping
668. Being very clear about what your utilization is going to be for the platform for the concept mapping
669. A description of what a concept mapping is
670. A description of how concept mapping has been used
671. A description of how concept mapping is relevant to the current project
672. We need to talk about how concept mapping is the right fit for answering the research question
673. A description of what the brainstorming question or focal prompt was
674. The focal prompt is critical
675. A description how/why people were sampled
676. Justification on the sample size
677. Justification on how sampling fits the goal of the research
678. Description of the steps you are implementing in the concept mapping
679. Explain the methods based on the steps in concept mapping (It is difficult to disentangle the methods from the results because they build on each other)
680. The mode of conducting the brainstorming sessions (online or in person or a mix)
681. Description of how the items from the brainstorming were used to generate the main list to use for sorting and rating.
682. Information on how many items did you come up with
683. The mode of conducting the card sorting task (online or in person or a mix)
684. It is fair to report the level of engagement of the participants.
685. A description of the results
686. Present those visuals (maps) in your paper
687. I think it is important to explain a point map (because it helps people to walk through why it's important, but that's also part of the method as well)
688. Details on how the final cluster solution or map was determined
689. How did you determine the final cluster solution?
690. Describing who was involved in the decision making should be explained
691. Presenting the concepts with illustrated items in each
692. The stress test is a good one to present how good is the fit
693. The correlation coefficient can be helpful if we are looking at different rating scales.
694. Tell the pieces and show how you get from one thing to the next
695. Not just the outputs, but the process of how you got there is critical
696. It is fair to report some of the challenges that you experienced during the study (for example, sometimes there are issues related to literacy level and comfort level with sorting, and if that's a real issue, how much should certainly work for the project, it should certainly be addressed in the limitations – and how we addressed those challenges)
697. It is important to report how we addressed those issues
698. We need to explain what you be done with the results (how the work is going to be used, where does it take us)
699. We need to present a good rationale of why we are doing the study
700. The conceptualization of the study needs to be made explicit
701. The background needs to present the rational of doing the study
702. The background section should include information leading to the study design
703. We need to justify in the introduction on why we are doing a concept mapping

704. The methodology needs to be briefed
705. People need to report exactly on all their stakeholder groups
706. The process of development of the focus prompt should be explained (how was the focus prompt developed in relation to the study aim)
707. Report on how stakeholder groups were identified
708. Details on the recruitment of the stakeholder group
709. A detail on the composition of sample as it applies to the study aim
710. Sample size is important
711. How did the researchers make sure that they recruited people with a proper representation of the stakeholder group?
712. How did the researchers ensured diversity of participants within the stakeholder group?
713. Mode of data collection – how were the initial data collected
714. Process of how interview data was processed and made into statements
715. How were the statements consolidated/refined for further analysis?
716. Who collated the statements?
717. How were people identified for sorting?
718. How was the sorting done?
719. Was there any overlap between participants who generated the statements and sorted statements?
720. General statement of how many people took part across the different phases of the study
721. Tabulation of the final statements in the analysis along with the ratings
722. A priori statement of what the acceptable solution would look like in terms of cluster analysis
723. What criteria was used to decide on the optimal cluster
724. Graphical representation of the final cluster solution
725. A detailed discussion on how many clusters were decided
726. Who was involved in final cluster solution development and refinement?
727. Was there any feedback on final cluster solution from the stakeholders?
728. Qualification and training of people undertaking the concept mapping study (research and data analysis)
729. Information on what are we going to do with the results
730. In the discussion, there should be application of the results – how the results are going to be used
731. Summary of the findings – and how does fit with the bigger literature and how it will help us
732. Limitations of the study
733. Some basic information about the focus prompt for the study
734. Information about who the participants are
735. Justification of why selected stakeholders are experts in the topic that is being discussed
736. We need to report methods used for developing the brainstorming tasks (focus prompt)
737. We need to report methods used for conducting the brainstorming tasks (can be conducted using surveys, literature review, systematic analysis, clinical guidelines, best practice guidelines, in-person/online brainstorming, and researchers can even generate an initial list of potential statements and tell the participants to provide feedback and incorporate this feedback to supplement the statements)
738. Present how many how many statements were generated through the statement synthesis
739. We need to report the decisions made about which data to include for card sorting task
740. Present the final number of statements that were included in the sorting rating process
741. We need to report methods used for developing the card sorting tasks
742. We need to report methods used for conducting the card sorting (online/paper based)
743. Presenting what the rating questions have been asked
744. Presenting the scales that have been used for the rating questions
745. Report on the number of people participating each of the different phases of the study
746. Reporting how many participants completed which parts of the study
747. Information on incomplete data should be reported (number of participants who did not complete the card sorting exercise - clustering and ranking)
748. Criteria used for excluding any data generated during the study should be explained
749. Presenting the mean and standard deviations of statements for each rating question
750. Information on some measure of variance in the rating
751. Reporting differences across the rating questions within the subgroups may be valuable depending on what question is being explored
752. Presenting data as a visual rating point map can be useful (may not always be important)
753. The cluster map should be presented
754. Then the basic information of the concept map itself
755. Presenting the mean value for each cluster

756. Presenting data as a rating cluster map can be useful
757. The generation of a similarity matrix can be useful
758. It is important to present the stress value for the map
759. We need to describe the interpretation of the stress value
760. It is relevant to present the bridging values that are generated
761. Report on the demographic factors that are relevant to the study (Demographics need to demonstrate that the person who is participating has some degree of expertise in the topic being discussed -there is no single set of demographic information that is relevant to every study. For example, age itself is always not an important component to report)
762. Demographics need to be reported for any type of subgroup analysis.
763. There is always a process of discussing whether system generated concept map is the most appropriate representation of the data or whether there is a need to redraw some of the boundaries to present that data in a more meaningful way
764. We need to present either via the map or in the text information about any additional interpretation of the data beyond the purely statistical interpretation
765. We need to report redrawing of any boundaries in cluster maps that have been done based on that data interpretation should be presented with overlapping maps indicating how the boundaries have been redrawn.
766. Some supplementary data to clearly present how statement synthesis process was done is valuable
767. One other component might be the opportunity for journals to provide supplementary information to support published studies
768. The title should describe the core problem being investigated and the participants
769. The title should describe the participants
770. Abstract should clearly state the study had used concept mapping approach
771. Abstract should have a clear description of the different participant cohorts
772. Abstract should explain the traditional core objectives of the study
773. Abstract should reflect on the different analytical approaches for the study
774. Abstract should reflect on the methodological steps
775. Introduction should include the ideal methodological approach to address this research question
776. A justification as to why concept mapping was selected as an ideal methodological approach to answer the question
777. Introduction should cover a rationale as to why we selected concept mapping approach
778. The research question is clearly stipulated
779. A little bit about the methodology in the introduction section of the paper
780. We need to provide a detailed description of the participants
781. We need to detail how participants were recruited
782. We need to provide demographic information of the participants
783. We need to outline each of the steps of concept mapping process
784. The approach used for the different participant groups at each phase of the concept mapping study needs to be explicitly stated (for example, if they were recruited differently, if brainstorming was collected differently…you had consumers and clinicians and clinicians do it online and consumers in face-to-face)
785. We need to detail the environment in which brainstorming exercise was conducted (for example, what did the room looked like, was it a neutral room, was it in the researcher's office… everything that can potentially influence ideas generation should be explained in detail because that can put pressure and bias on what comes up)
786. Was the brainstorming sessions conducted separately for the different stakeholder groups needs to be clearly mentioned (Were the participants being mixed, or were they separated and for what purpose? For example - so that they can feel more comfortable talking about each other)
787. It is important to describe the role of the moderator of the brainstorming session (to look for potential researcher induced bias… need to talk about what their potential biases and lenses are to make sure that they are delivering the question in a neutral time and how they interact with the group)
788. We need to provide some detail on how much interaction occurred within the group (group dynamics during the brainstorming session… some commentary around the level of engagement and participation of the people in the room in a brainstorming activity, the feeling in the room, were the participants were working collectively and adding to each other's and in a constructive way, or whether there was this, I guess, a tone of disagreement, if anything else happened in the session)
789. We need to report what recording equipment was used
790. We need to report on how the sessions went
791. We need to document how long the brainstorming sessions went for.
792. We need to give a number to how many brainstorm segments were yielded overall.
793. We need to report how brainstorming data was transcribed/translated

794. We need to describe the approach used to reduce the statements (some people have a goal for the final set of statements, some leave similar statements, and some retain similar statements- it can influence the rating and clustering task; if we have statements that are so similar, they will almost be grouped together because of a similarity)
795. We need to detail the level of engagement of the research team on editing and refining the statements
796. We need to describe the researchers' involvement in either removing redundancies or retaining similar statements
797. We need to provide some examples of what the authors felt was redundant or duplicates to clarify their approach
798. We need to give information on how many statements were used for the subsequent grouping and rating activities
799. We should report the exact wording of the statements used card sorting tasks
800. Need to tell how card sorting task was conducted (in person or online, whether they have done it individually or sitting next to each other and helping each other; whether participants were feeding onto each other or participants were individually doing their grouping and rating tasks)
801. Need to tell the level of support provided to the participants to undertake card sorting tasks.
802. The rules given to the participants for card sorting task should be described
803. We need to report the instructions given to the participants on the number of groups they can create
804. We need to report the mean and the range of the number of groups per participants (so that we can make that judgement on ourselves whether if that fits over analysis by the research team)
805. Reporting of the stress index
806. Applying the split half reliability test to measure the validity of a map should be a mandatory step (randomly divide participants into two groups and then create two maps and then use to compare if they are essentially same with each other to check the reliability of the validity of the whole cohort map; it helps to make decision as to whether a further investigation of the data should be done by the cohorts being combined or separate)
807. We need to provide a reference for the reporting the reliability/validity testing (stress value, split half reliability)
808. We need to undertake a Welsh t-test if we analyse the pattern/ladder graph to see if there is a statistical difference between the ranks.
809. It should be strongly recommended to test the level of significance whenever a ladder graph is generated
810. A go-zone graph should be used to get cohort comparisons (comparing participant groups may not be reliable if they do not produce equal contribution in brainstorming, card sorting tasks, and map interpretation because the people perhaps more likely to vote their own idea as higher depending on what the topic is about and what is put in front of them)
811. We need to clearly describe the contribution of different participant groups during the different phases of the study
812. We must think about the precise wording and make sure that wording of each statement truly makes sense with each of the rating questions (we need to somewhere really emphasize that the wording for these statements – that all be whether in the third person or the first person or whatever makes sense with the dataset. If proper wording are not used – participants may be confused on how to rate a task and final output becomes somewhat vague and we cannot interpret it properly)
813. We should include participant demographics by cohorts.
814. If there is a demographic question specific to a population, we need to add some point of references to say whether that is normal (for example, if you're saying that I'm generally reporting participants using hearing aids for hours day, some point of reference to say that this is significantly lower than what is generally reported on hearing aids use)
815. We need to be clear who has made what decision whether you put that in methods or results
816. We need to be very clear on who provided the description of the concept maps produced from the statements generated by the participants
817. We need to provide a clear description of how cluster configuration was selected (what rules we used for selecting cluster configuration
818. We need to be clear on how the axis were labelled (we need to be explicit that further clustering identification of domains across map is purely done by the researchers or the participants)
819. We need to tell how the map was generated
820. We need to provide information on the different axis on the map
821. We need to make it clear to the readers how the map should be interpretated
822. We need to talk about the bridging scores (helps to provide the rationale behind map selection))
823. We should use the Cronbach's alpha for an estimate of internal consistency
824. We should undertake a test of significance to the rank order data of the ladder (pattern match) graph
825. We need to present any analysis that helps to determine the validity of the go-zone graph
826. Discussion should focus on the purpose of the paper on the first place.
827. There should be a limitation section
828. Limitation section should explicitly state omissions or deviations from the concept mapping methodology and analysis (for example, rating, Cronbach's alpha, split half reliability testing, stress value)
829. A full disclosure on all the steps taken or not taken

830. Conclusion should be the summary of the core findings from the study
831. We should reference the original creators of the methodological approaches
832. The context in which the group concept mapping exercise was conducted should be reported
833. The focus prompt should be stated
834. A description of the focus prompt development process should be included.
835. A description of the participants sample should be included
836. The approach used to generate participants sample should be included.
837. A clear description of how researchers approach their sampling for group concept mapping
838. A clear description to the idea generation phase (Brainstorming being one of the techniques that someone would use to generate ideas, but it's not the only technique. Brainstorming occurring during a video interview is different than say somebody logging into the site and generating asynchronous brainstorming statements to a focus prompt - there needs to be a distinction about how researchers are going towards generation of ideas)
839. Some clear distinction about the mechanisms used to engage around brainstorming (we need to have a clear indication of how they're going about accessing their participants, whether that's via some technology or whether that's interpersonal and in a face-to-face setting)
840. How long did someone spend during brainstorming?
841. How long was the brainstorming/idea generation period open?
842. We should be reporting on length of engagement around the different phases
843. The total number of statements that were generated during the process needs to be reported
844. We need to describe/detail the idea synthesis step.
845. We need to report on a sorting participant.
846. We need to report how sorting/rating activities are sequenced (Whether people are doing sorting and rating together as one activity, or whether sorting and rating is two separate activities)
847. The authors should report on their quality review process for sorting/ranking task (whether any data sets were rejected - basically saying, how many good data sets were included versus how many problematic data sets were excluded)
848. Authors should report the statistical package (and the version) they have used
849. The authors should clearly mention the underlying analytical steps carried out in a software package to better interpretate the results
850. A report on the outputs of the mechanisms for analysis (multi-dimensional scaling and hierarchical cluster analysis)
851. It is important to indicate how many times the multi-dimensional scaling analysis ran before it converged on its final representation.
852. It is important to discuss the quality of the multi-dimensional scaling output (For example, stress value is one of those indicators used as a function of understanding the degree of fit between the similarity matrix and the and the final point map for example; if we report a stress value of 0.4, we need to explain what it means and contextualize or explain in relation to goodness of fit with other concept mapping study) ,
853. It should be clear to a reader how the final decision for the cluster map solution (in a group of researchers, decisions made with an external stakeholder group outside of the map process will come, or by an individual researcher)
854. A detail on how the interpretation session was carried out
855. Information on who facilitated the interpretation session
856. Information on who was involved in the final labeling of the cluster map
857. Authors should report on the steps they went through to agree upon the final cluster solution.
858. If the authors have conducted a cluster level ratings analysis, they report pattern matches, go-zones, and correlation value of those patterns.
859. Authors should report the distribution of the ratings across those patterns.
860. Any further analysis specific to a software package needs to be transparently reported
861. If the authors have done an item level ratings analysis for each cluster, they should report a R value
862. If authors conduct means tests between clusters, they need to report t-test output (mean values, variation, t-score, p-value, and degrees of freedom)
863. Any adjustment made in the cluster map should be reported (for example, if cluster boundaries have been redrawn, or items relocated in different clusters based on that interpretation)
864. We need to be transparent on qualitative, quantitative and mixed-methods components of concept mapping process (must be comprehensive enough to incorporate all the qualitative, quantitative, and mixed methods elements that are associated with group concept mapping)
865. A manuscript should have a good description of relevant literature
866. Study report does not need to include deep details of the analysis but very least need to explain what is done (multi-dimensional scaling, hierarchical cluster analysis, or principal components analysis)
867. Need to mention the analysis that is used in the study (not the name of the software)
868. We need to add a description of the analytical tool that is used

869. It is appropriate to say the statistic algorithm if SPSS, R, or SAS software is used for analysis
870. Need to explain why a group is a way it is (for example, sorting group is often smaller and more involved in the project because the response rate for sorting is quite high)
871. Response rate for rating
872. We can get into the issues of sampling while we talk about sorting
873. Rating (value data) is the most important way to collect data, but it is not the only way
874. We need to look for patterns within the clusters/whole data
875. Sometimes the pattern differences between two groups or two measures are important
876. We could look at the item ratings to look at the patterns
877. Cluster rating map (to look for pattern differences between measures/groups) could be included based on researcher's decision
878. It should be a conscious decision whether to include cluster rating map or not
879. A good estimate of map or sorting is the stress value which is not usually reported.
880. We need to explain the type of data (ordinal, nominal, or ratio) for which we are reporting the stress value.
881. Need to provide a stress value and compare it with relevant literature
882. There should be clarity about what kind of stress value is computed.
883. Need to explain about the reliability of the map
884. Research and proprietary studies may have different aspects on reporting
885. Polishing a study needs clear methods and analysis with a critique
886. Rationale for why the groups are composed
887. Rationale for where the groups are (sometimes sample of convenience, sometimes random sample of a group of population)
888. A rank order rather than the actual mean value for the ladder graph (ladder graph helps to understand where the agreement is, where there is a lack of alignment and facilitated discussion on that)
889. Eigen values are very important in reporting (it is because the eigenvalues give indication of how much of the variance is accounted for in the in the concept mapping)
890. I would say most important thing to report is the images.
891. How the images are setup?
892. We need to show the eigenvalue above the eigen vectors in the report.
893. Exclusion of the ratings/sorting carried out randomly.
894. People who do not complete sorting need to be excluded (people who just answer the ratings that are not interested in teamwork)
895. Point map and description of the dimensions (left, right) is important
