# Supplementary material for: Development of a Reporting Guideline for Trochim’s Concept Mapping
Source: Methods Protoc. 2025 Mar 3;8(2):24. doi: 10.3390/mps8020024 (PMC11932253; doi:10.3390/mps8020024)
Supplement: Supplementary file 1 [file mps-08-00024-s001.zip › Supplementary document 6, statement and cluster rating score.pdf]

List of statements in each of the 11-clusters

Cluster/Statement Description	Mean (± SD)
**Cluster 1 Rationale and description of the concept map (4.08 ± 0.6)**	
85. Describe the final product [clusters and axis] of the concept mapping research.	4.76 (± 0.7)
92. Present the final number of statements included in the card sorting (clustering and ranking).	4.4 (± 0.9)
55. Make it clear to the readers how the map should be interpreted.	4.2 (± 1.1)
45. Relationship of the cluster and statements [should be described]	4.08 (± 1.2)
15. Examples of the statements to demonstrate the individual clusters.	4 (± 1.2)
22. An overview of the results [for each stakeholder group] per stage.	3 (± 1.4)
**Cluster 2 Title and abstract (3.56 ± 0.4)**	
44. Summary of how study findings fit with the bigger literature and help us.	4.24 (± 1.06)
56. Describe how the concept map will be utilized.	3.96 (± 1.06)
58. The title and abstract clearly states the study used a concept mapping approach.	3.84 (± 1.35)
38. Explanation of why concept mapping [in background] is the right solution for this research question.	3.8 (± 1.33)
57. The conclusion is a summary of the core findings from the study.	3.72 (± 1.43)
87. A manuscript/report has a good description of the relevant literature with proper references.	3.64 (± 1.23)
11. The title and abstract describes the core problem being investigated.	3.6 (± 1.61)
23. The abstract contains basic information about what we found.	3.48 (± 1.48)
61. The abstract reflects on the methodological steps.	3.04 (± 1.31)
69. The abstract reflects on the analytical approaches for the study.	2.92 (± 1.42)
78. Abstract has a clear description of the different participant cohorts.	2.92 (± 1.29)
**Cluster 3 Process for statement reduction (3.4 ± 1.0)**	
41. Information about the total number of statements generated from the participants.	4.08 (± 1.0)
60. Provide some examples of what the authors felt was redundant or duplicate statements.	2.72 (± 1.1)
**Cluster 4 Selecting cluster solution (3.35 ± 0.2)**	
81. Look for patterns within the clusters/whole data.	3.6 (± 1.0)
24. Talk about cluster thickness to show the relative importance of each cluster.	3.48 (± 1.3)
34. Give a few examples of the cluster range data (least and most important clusters).	3.48 (± 1.1)
42. Report a higher-order interpretation of the map (if done).	3.24 (± 1.2)
49. If we have a go-zone plot, include in a table into which quadrant each item falls.	3.2 (± 1.4)
39. Report on ladder plot if we want to see the comparison between stakeholders.	3.08 (± 1.1)
**Cluster 5 Selecting cluster solution (3.3 ± 0.5)**	
1. Provide a clear description of how cluster configuration was selected.	3.84 (± 1.0)
5. Any adjustments made in the cluster map should be reported.	3.84 (±1.0)
12. Note of how we managed outstanding items (during data analysis) that do not belong to any clusters.	3.48 (± 1.1)
30. Note on how many cluster solutions were reviewed before the final solution was determined.	3.48 (± 1.5)
80. Information on the Likert scale used for rating question.	3.28 (± 1.3)
77. Information on incomplete or excluded data is provided.	3.04 (± 1.5)
72. Justify the rationale behind the rating scale.	2.96 (± 1.1)
94. Some supplementary data to clearly present how the statement synthesis process was done.	2.48 (± 1.1)
**Cluster 6 Sample size (3.28 ± 0.0)**	
9. Talk about the minimum sample size to have reliable structuring data.	3.36 (± 1.2)
37. Description of how we assessed the saturation of the conceptual space.	3.2 (± 1.2)

**Cluster 7 Process for recruiting and retaining stakeholders (3.27 ± 0.6)**	
40. The initial question or focus prompt used in the study is clearly (explicitly) defined.	4.68 (± 0.8)
35. Ethical considerations are detailed and discussed.	3.92 (± 1.2)
25. Explain how researchers ensured broad representation within the stakeholder groups.	3.68 (± 1.1)
6. Justify the different stakeholder groups included in each stage.	3.64 (± 1.0)
3. Describe the contribution of the stakeholder groups during different phases of the study.	3.6 (± 1.3)
62. Any deviations from the study protocol are explained/justified.	3.6 (± 1.2)
16. A detailed description/justification of the phases of concept mapping that may include a flowchart.	3.48 (± 1.5)
86. Information on working with an advisory group (if involved).	3.12 (± 1.4)
10. A concept mapping research is transparent about the power dynamics.	3.04 (± 1.6)
28. Report the exact wording of the statements used for card sorting tasks.	3 (± 1.3)
26. Report if anybody influenced the selection of the participants.	2.96 (± 1.3)
71. If statements are returned to participants for validation, we should note which group was involved.	2.92 (± 1.2)
14. Justify why stakeholder groups were not involved in interpreting the map.	2.8 (± 1.4)
51. Any issues on language translation are to be reported.	2.68 (±1.0)
64. The planning phase of the study is clearly described.	2.64 (± 1.3)
74. Information on decisions to remunerate the participants.	2.48 (± 1.1)
**Cluster 8 Researchers' reflection on concept mapping (3.04 ± 1.0)**	
63. Talk about the limitations of the concept mapping project/process.	3.76 (± 1.3)
19. The actual experience of the concept mapping process is talked in the discussion.	2.32 (± 1.3)
**Cluster 9 Transparency of research reporting (3.04 ± 1.0)**	
73. Information about who was involved in interpreting the clusters.	3.56 (± 1.2)
20. Was there any feedback on the final cluster solution from the stakeholders?	3.52 (± 0.8)
7. The extent to which sorted material was managed or edited by the research team.	3.32 (± 1.3)
70. Information on how the card sorting [clustering and ranking] data is used in analyses.	3.28 (± 1.1)
27. Information on how credibility, trustworthiness was applied in interviews conducted during study.	3 (± 1.5)
32. Provide details on the origin of all statements.	2.48 (± 1.4)
46. Provide some details on how much interaction occurred within the groups.	2.12 (± 1.2)
**Cluster 10 Data analysis procedure (2.93 ± 0.5)**	
31. Present the stress value (with interpretation) for the map.	3.72 (± 1.4)
8. Describe how (hierarchical) cluster analysis was conducted.	3.56 (± 1.3)
84. Present the mean value for each cluster.	3.56 (± 1.4)
43. Report the mean and the range of the number of groups [clusters] generated by the participants.	3.48 (± 1.4)
52. Further analysis of the content within the cluster to identify the pattern in the data.	3.28 (± 1.1)
54. Information on multi-dimensional scaling.	3.04 (± 1.4)
29. The authors mention underlying analytical steps carried out [statistical algorithm] in software.	2.96 (± 1.4)
65. Presenting the mean and standard deviations of statements included in the prioritisation task.	2.96 (± 1.2)
68. Any further analysis specific to a software package is reported.	2.88 (± 1.4)
91. Report an R-value if the authors conduct an item level rating analysis for each cluster.	2.8 (± 1.3)
93. Report a test of significance to the rank order data of the ladder (pattern match) graph.	2.76 (± 1.4)
36. If authors conduct means tests between clusters, they need to report t-test output.	2.68 (± 1.3)
47. The correlation coefficient can be helpful if we are looking at different rating scales.	2.68 (± 1.1)
67. Applying the split-half reliability test to measure the validity of a map.	2.56 (± 1.3)
95. Use Cronbach's alpha for an estimate of internal consistency.	2.32 (± 1.1)
82. Show the eigenvalue of the eigenvectors.	2.28 (± 1.3)

50. Did you do any sensitivity analysis?	2.24 (± 1.1)
**Cluster 11 Methodological details (2.84 ± 0.6)**	
66. Approach used for [recruitment of] participant groups at each phase of concept mapping is explicitly stated.	4.08 (± 1.0)
4. Explain how the card sorting sessions were conducted.	3.52 (± 1.0)
88. Each of the methods used to generate the ideas [brainstorming] is carefully described.	3.48 (± 1.2)
53. Report how card sorting (prioritising and rating) activities are sequenced.	3.36 (± 1.2)
75. Instructions provided to the participants [for different tasks] is clearly described.	3.28 (± 1.1)
79. Information on how interview data was processed and made into statements.	3.24 (±1.0)
90. What was the process of developing the research/focus question?	3.2 (± 1.3)
59. Report how brainstorming data was transcribed/translated.	2.84 (± 1.1)
21. Talk about the [number of] brainstorming sessions.	2.76 (± 1.2)
33. Information on who facilitated the interpretation session.	2.76 (± 1.4)
2. Describe the role of the moderator of the brainstorming session.	2.72 (± 1.1)
48. Information on how much detailing [level of support] was required during brainstorming/card sorting.	2.6 (± 1.4)
13. How do we collect their [participants] demographic data?	2.56 (± 1.3)
89. Report on the details of pilots (if performed) to get our research prompt.	2.48 (± 1.2)
76. Was there any warm-up activities prior to idea generation?	2.16 (± 1.2)
18. Information on whether any [brainstorming] sessions was recorded.	2.08 (± 1.4)
17. Talk about the training (of the researchers) on group concept mapping.	2 (± 1.1)
83. A timeframe of how long to complete the individual stages.	1.88 (± 0.9)
**Rating score for the statement excluded from final analysis.**	
96. Information on the use of concept mapping software at different phases of the study	2.96 (± 1.5)
