# Supplementary material for: Development of a Reporting Guideline for Trochim’s Concept Mapping
Source: Methods Protoc. 2025 Mar 3;8(2):24. doi: 10.3390/mps8020024 (PMC11932253; doi:10.3390/mps8020024)

2-Cluster solution

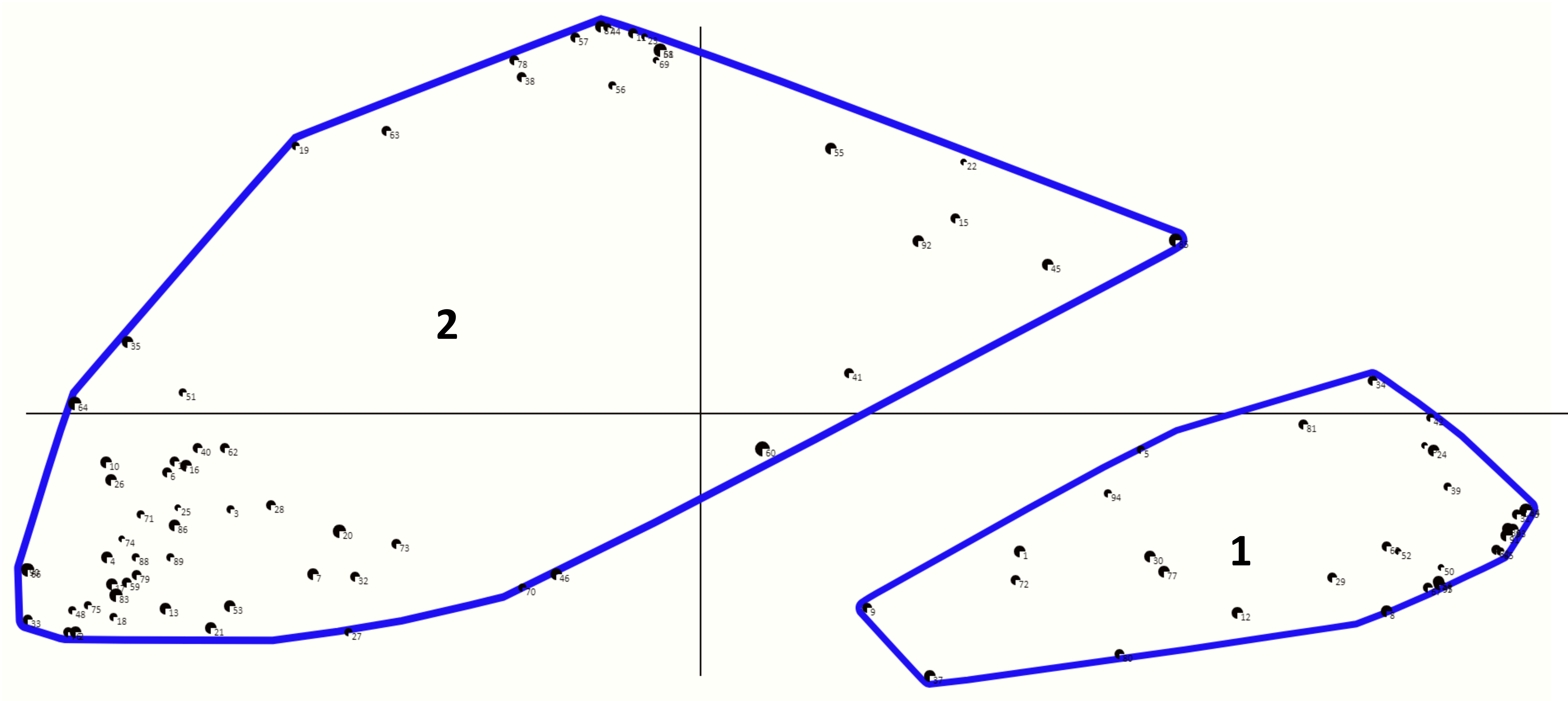

3-Cluster solution

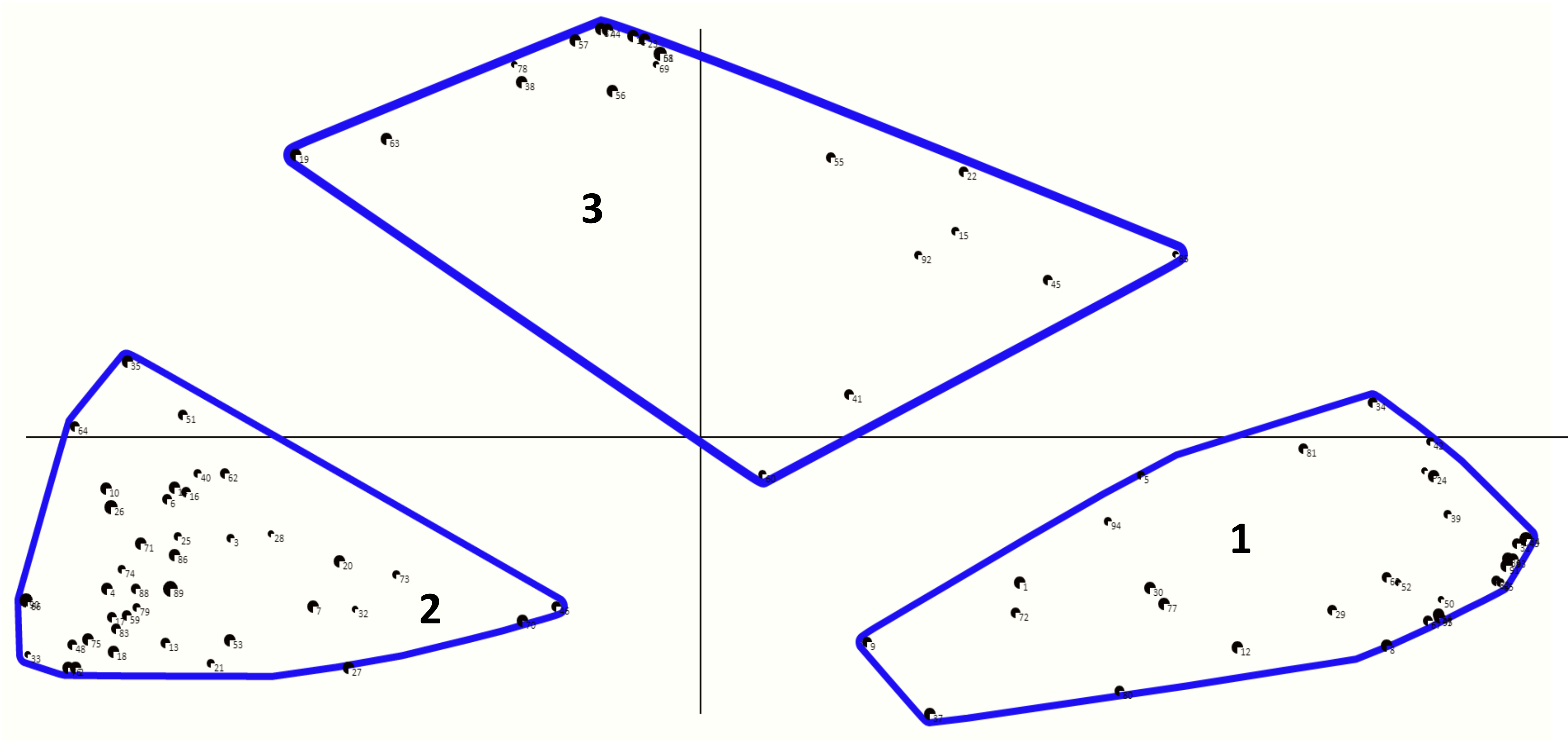

4-Cluster solution

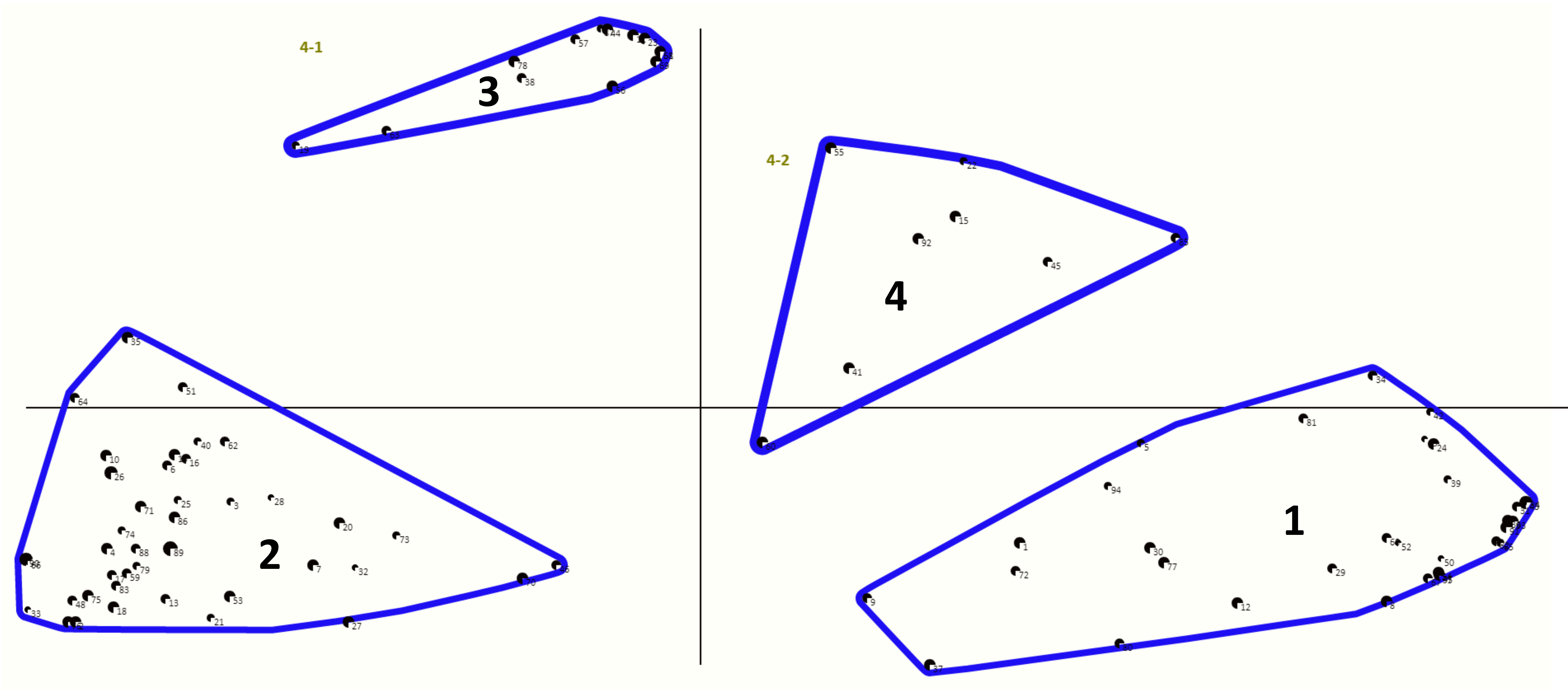

5-Cluster solution

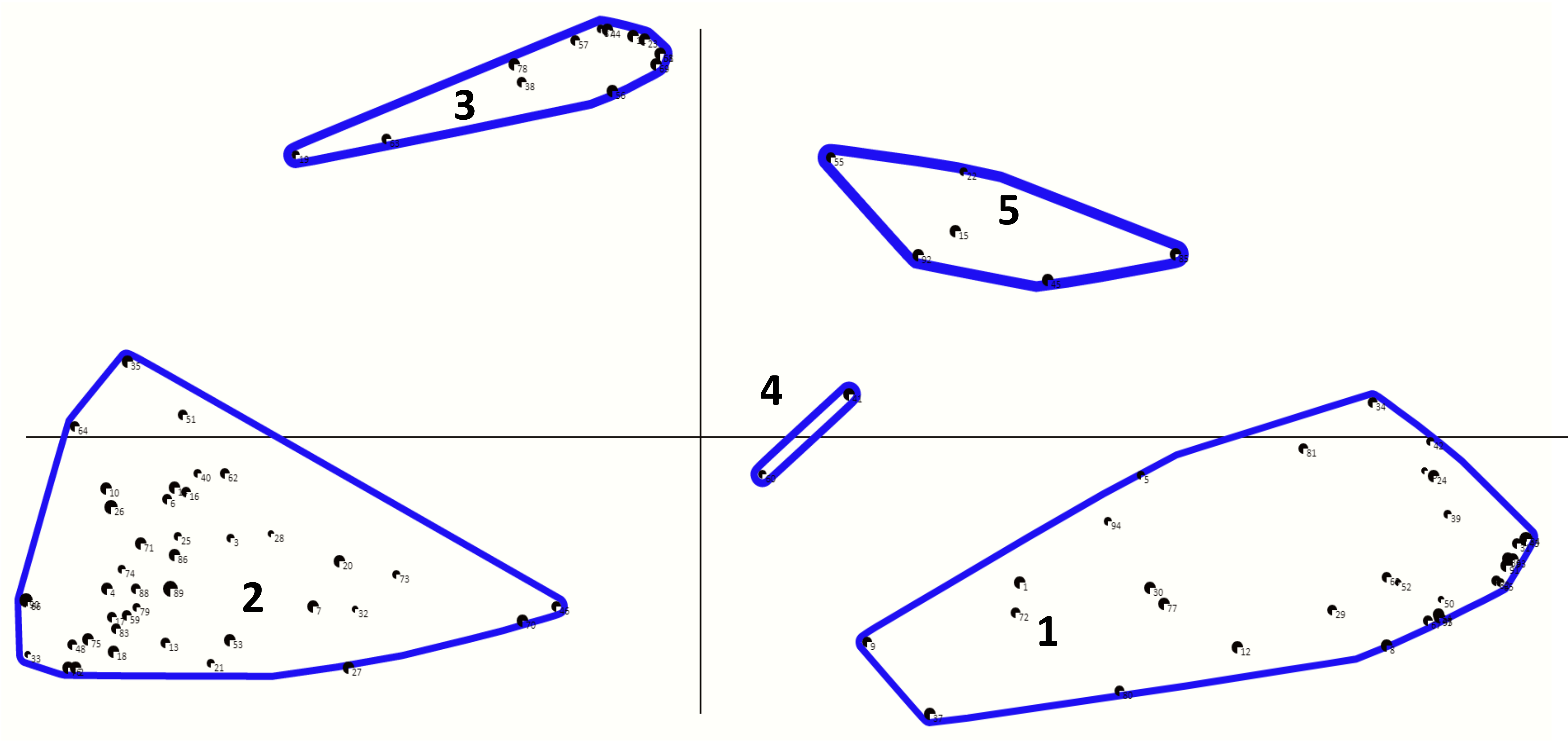

6-Cluster solution

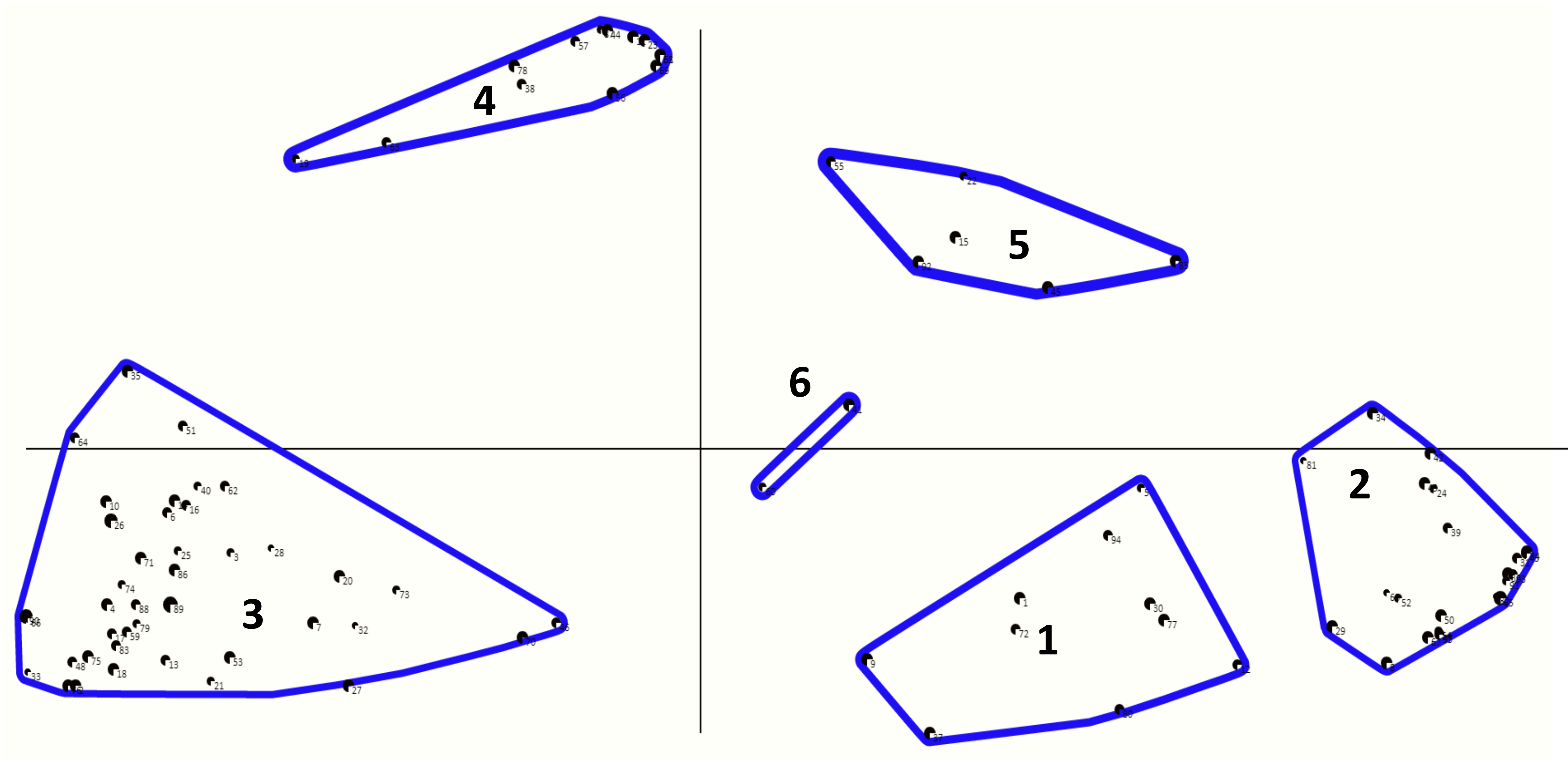

7-Cluster solution

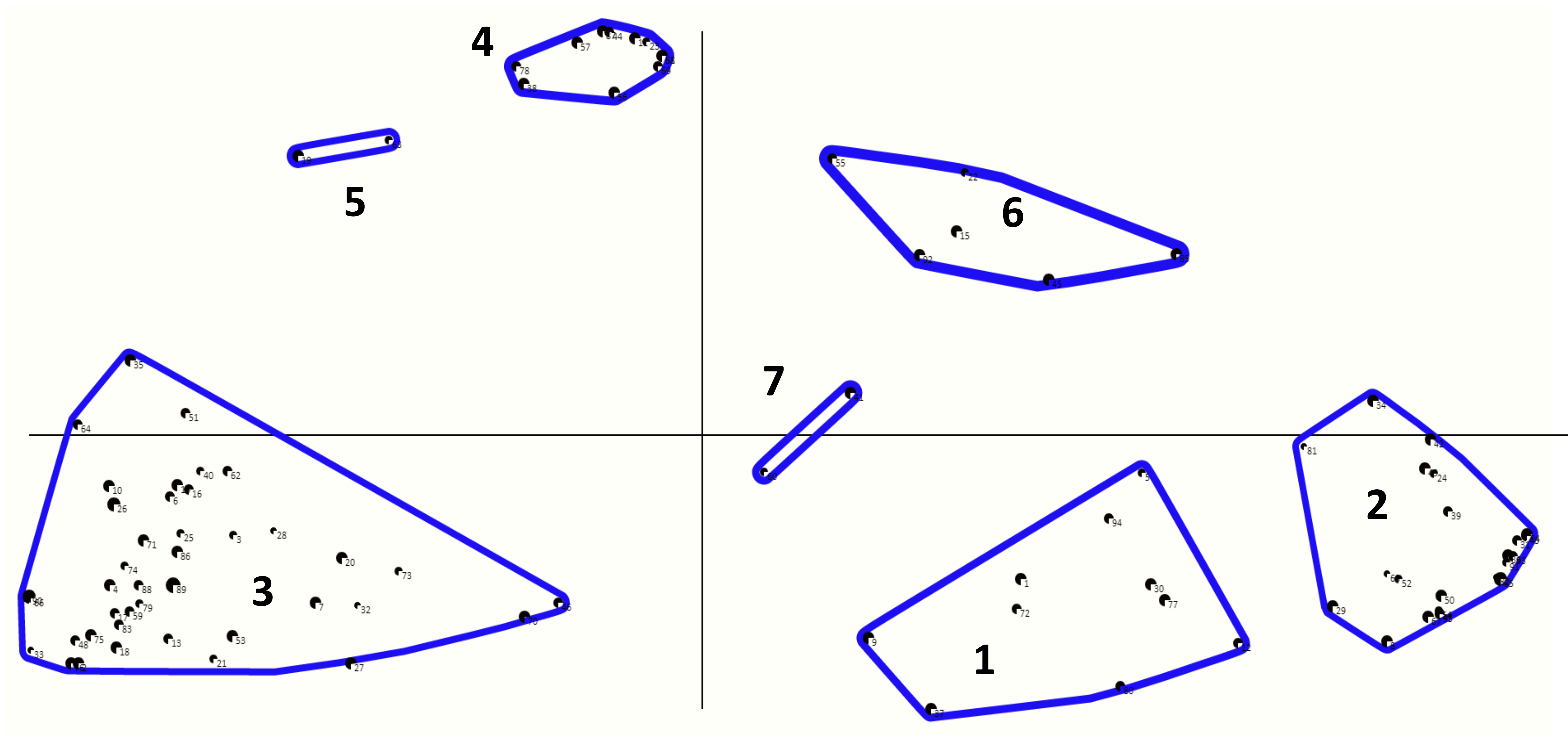

8-Cluster solution

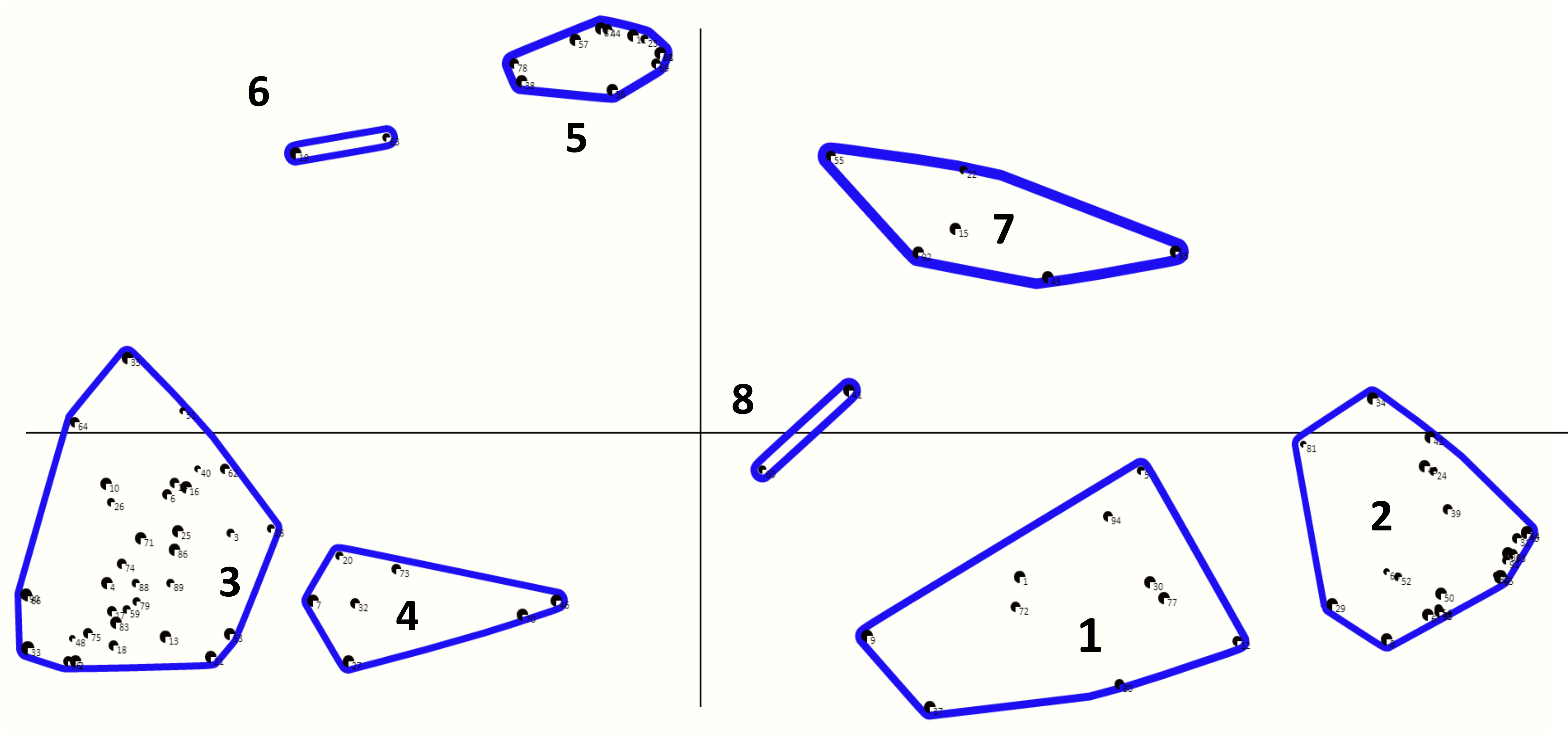

9-Cluster solution

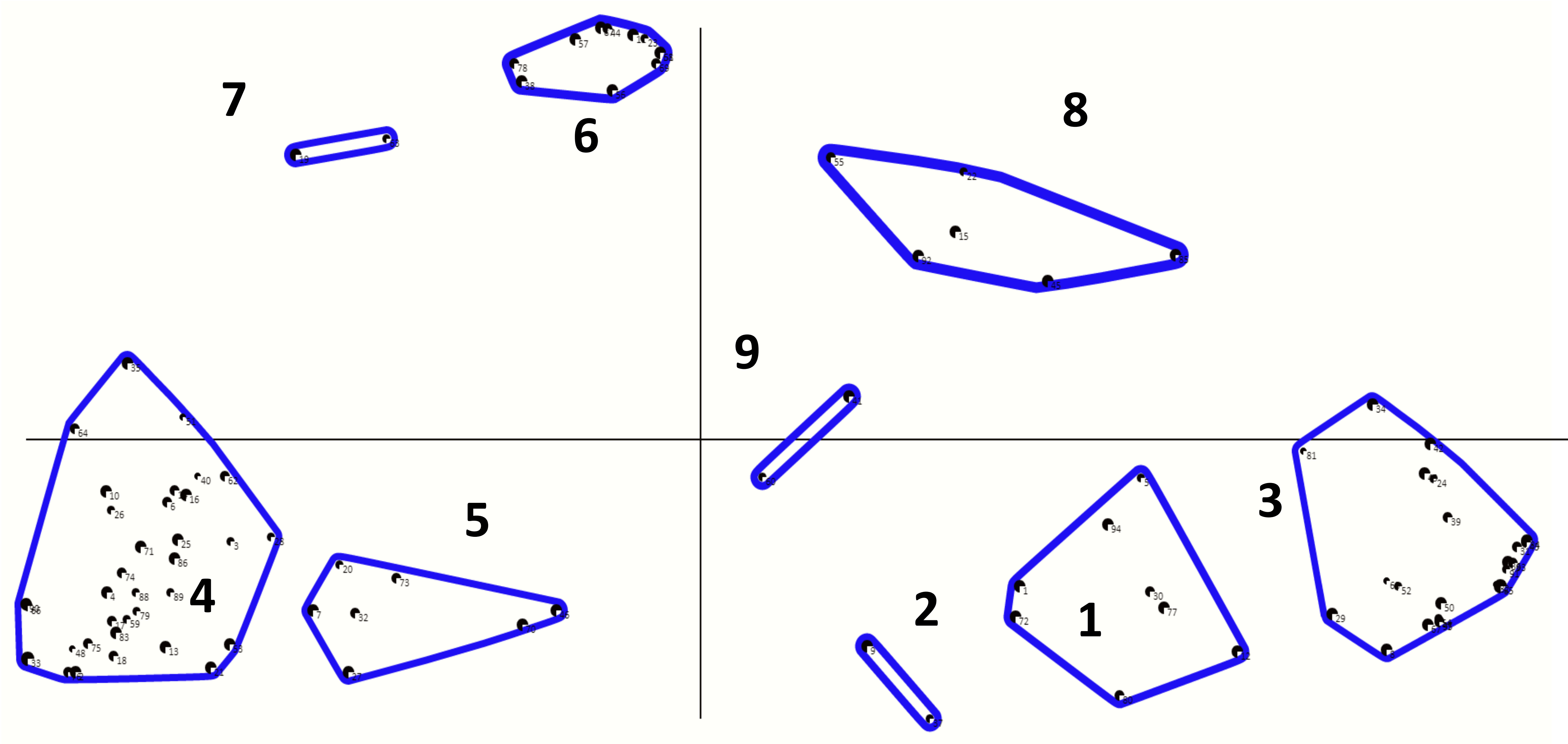

10-Cluster solution

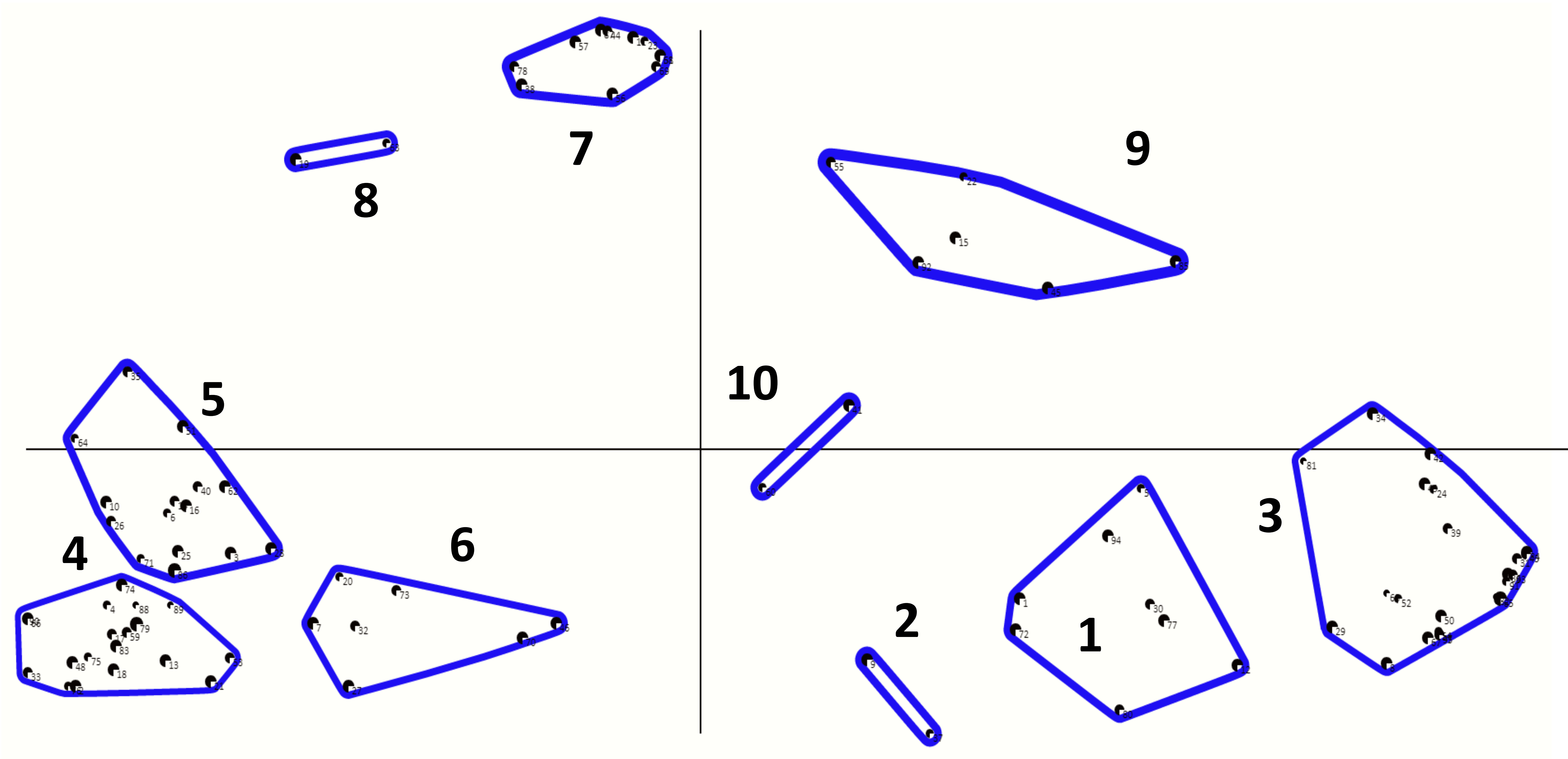

11-Cluster solution

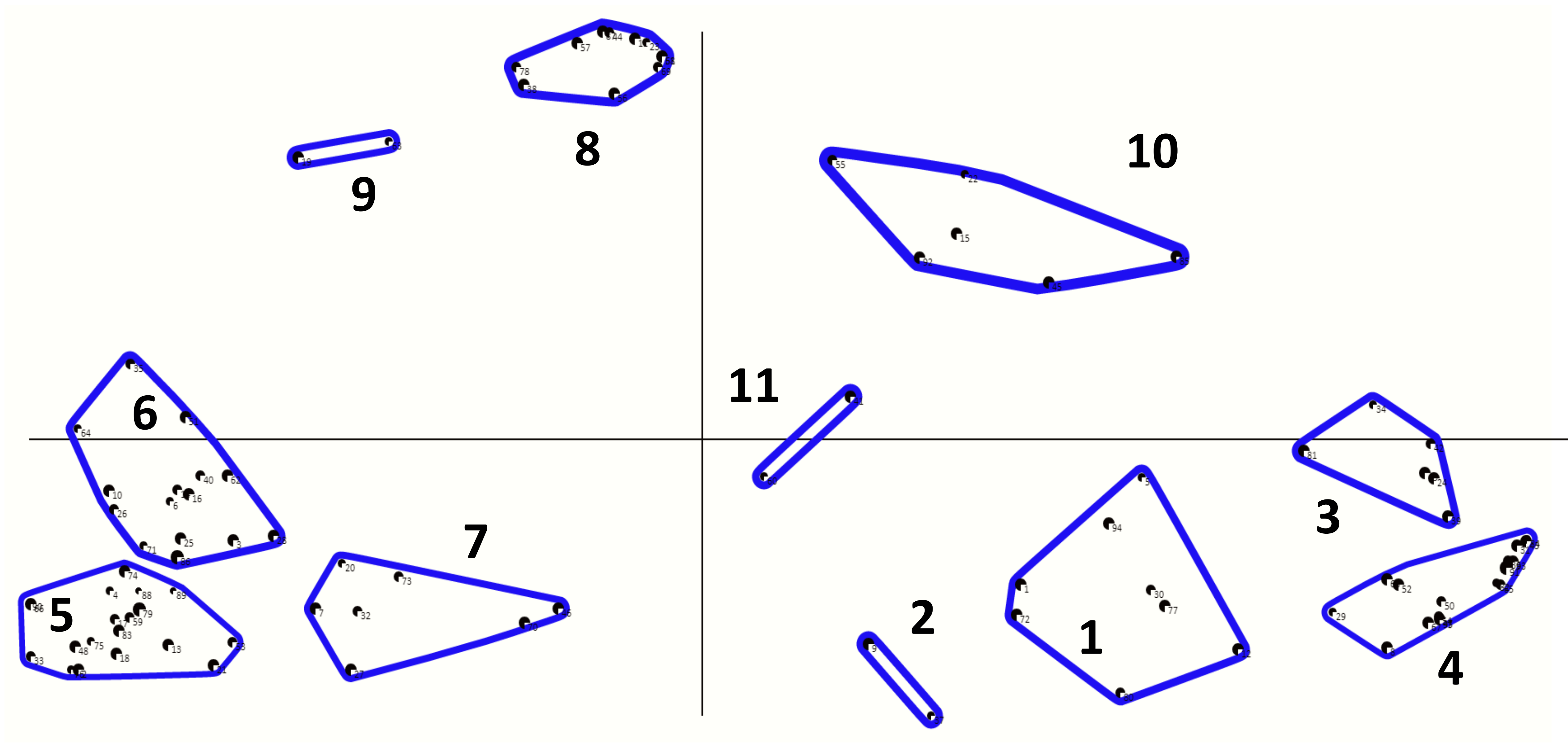

12-Cluster solution

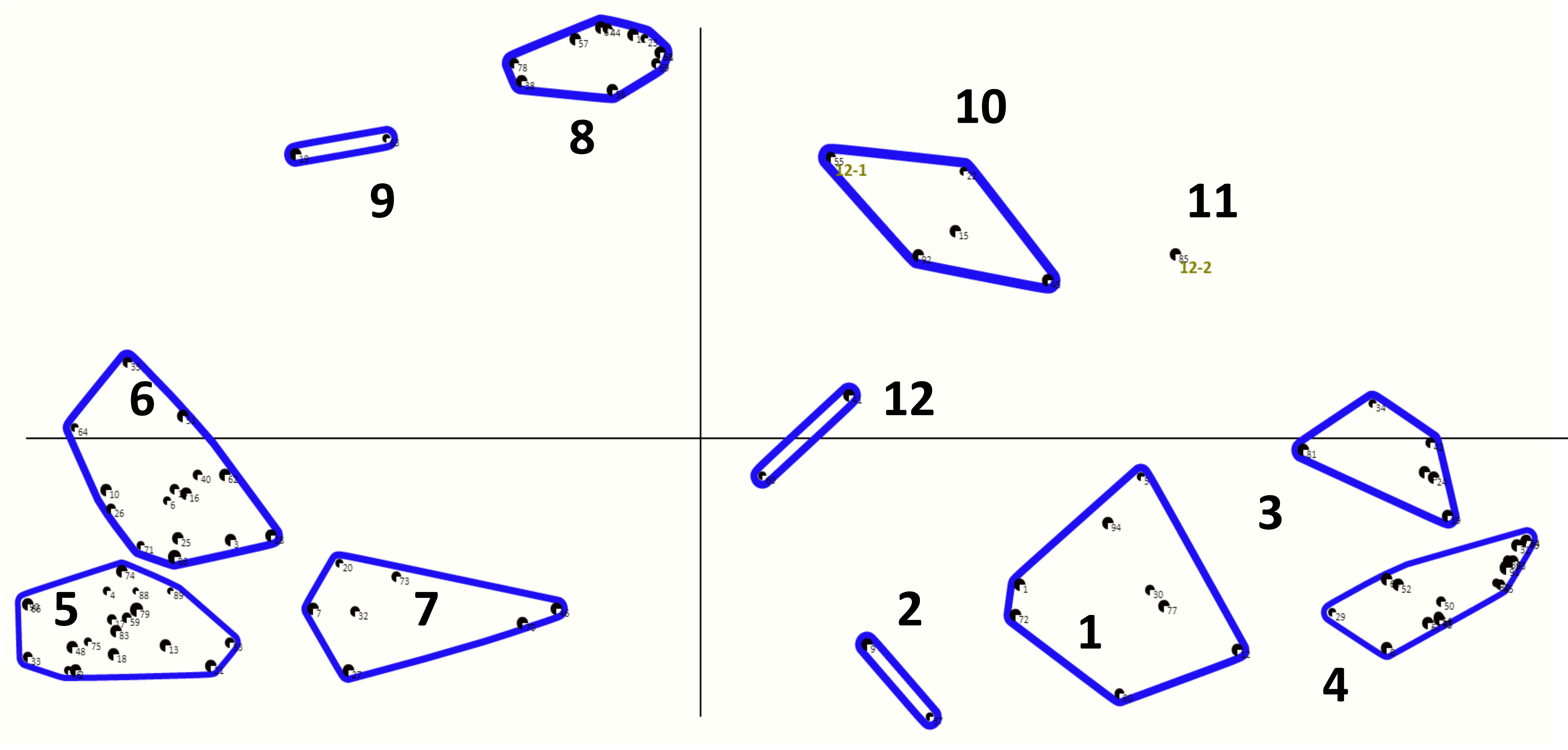

13-Cluster solution

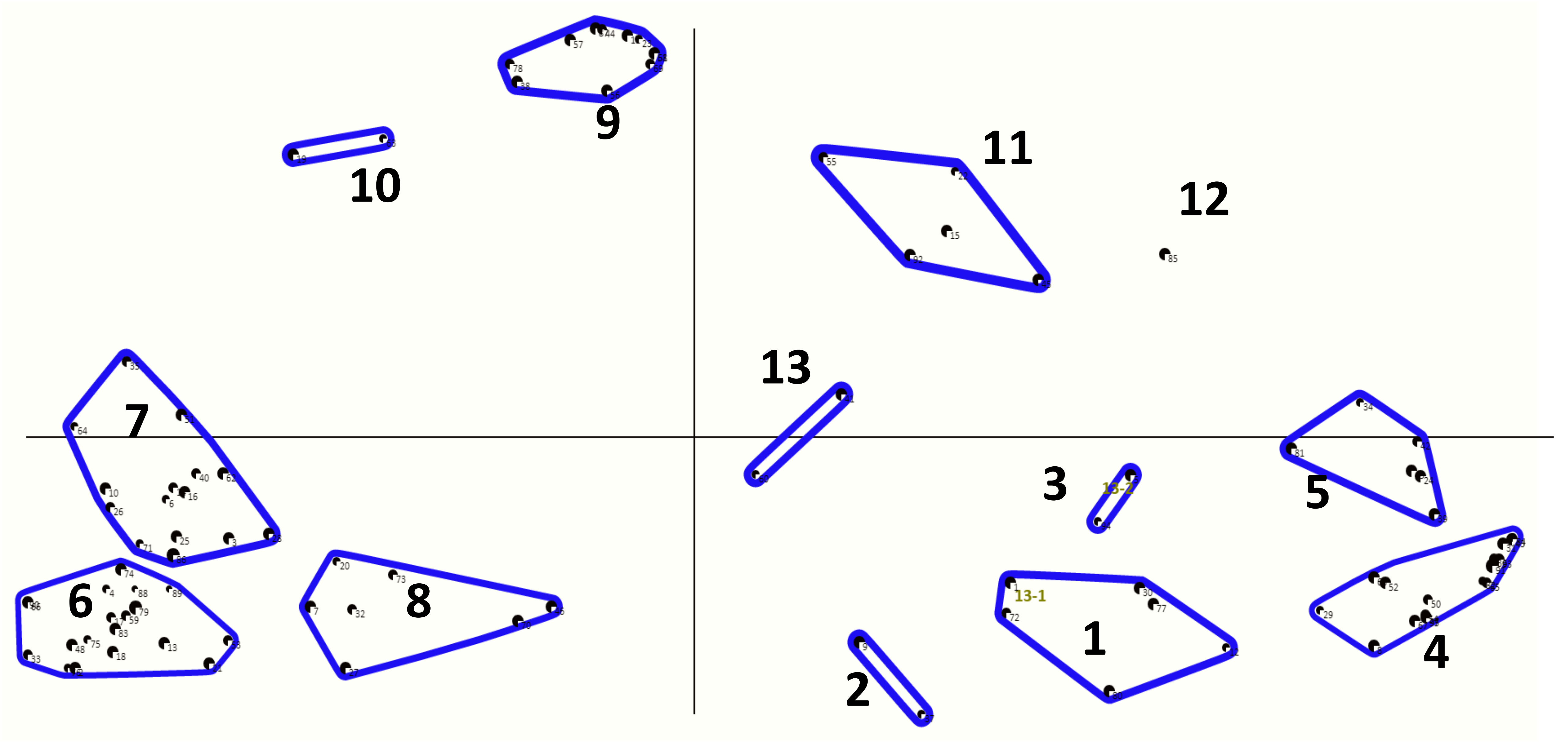

14-Cluster solution

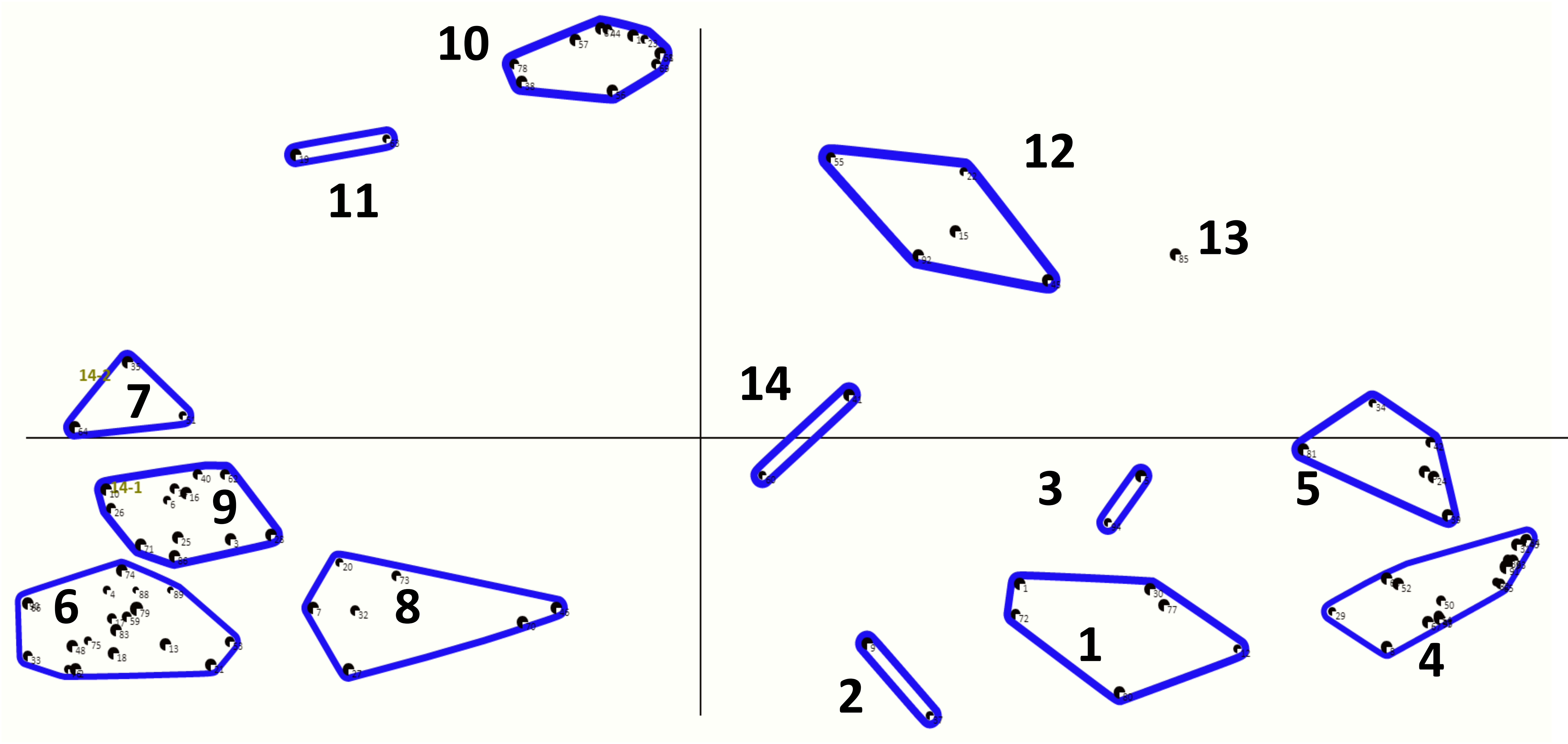

15-Cluster solution

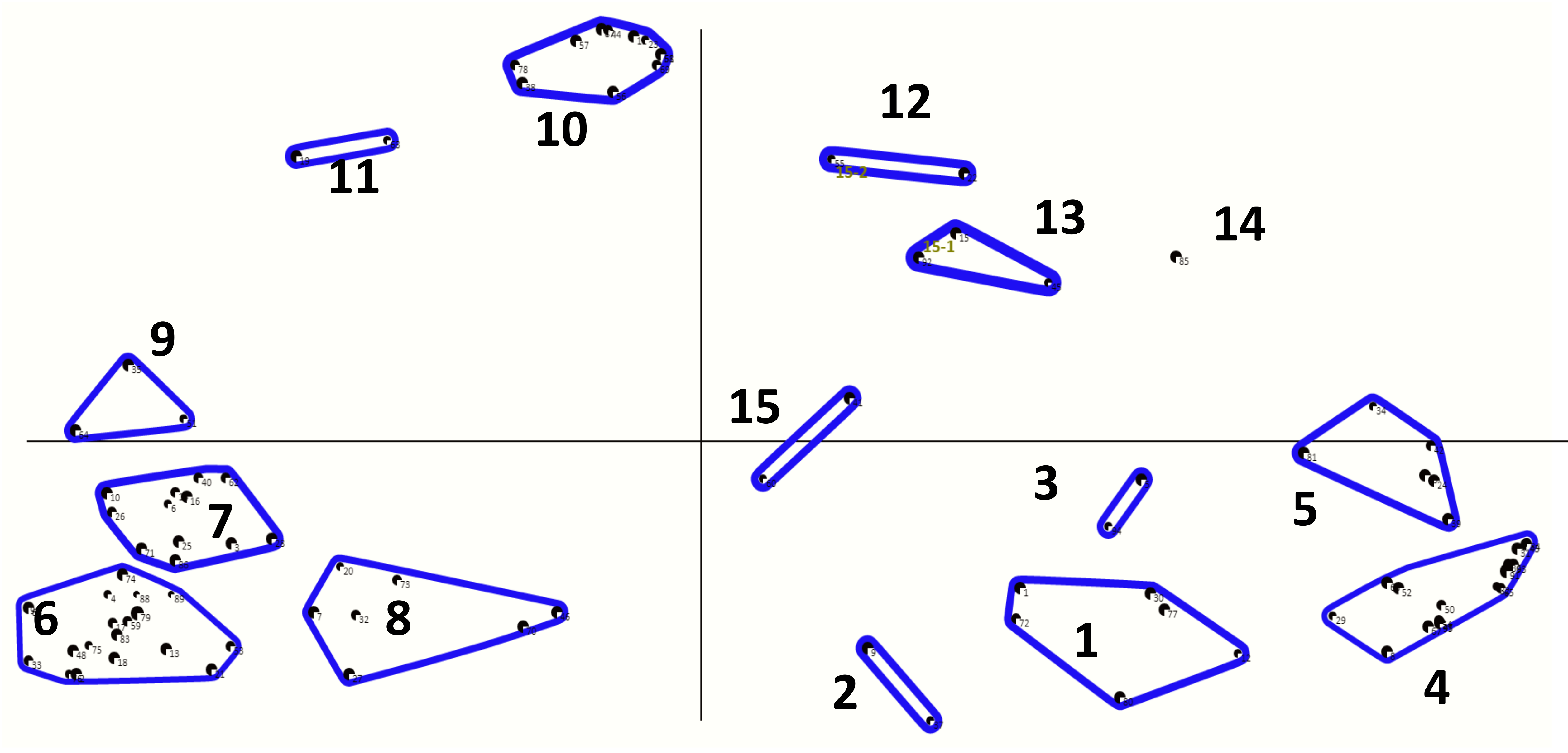

16-Cluster solution

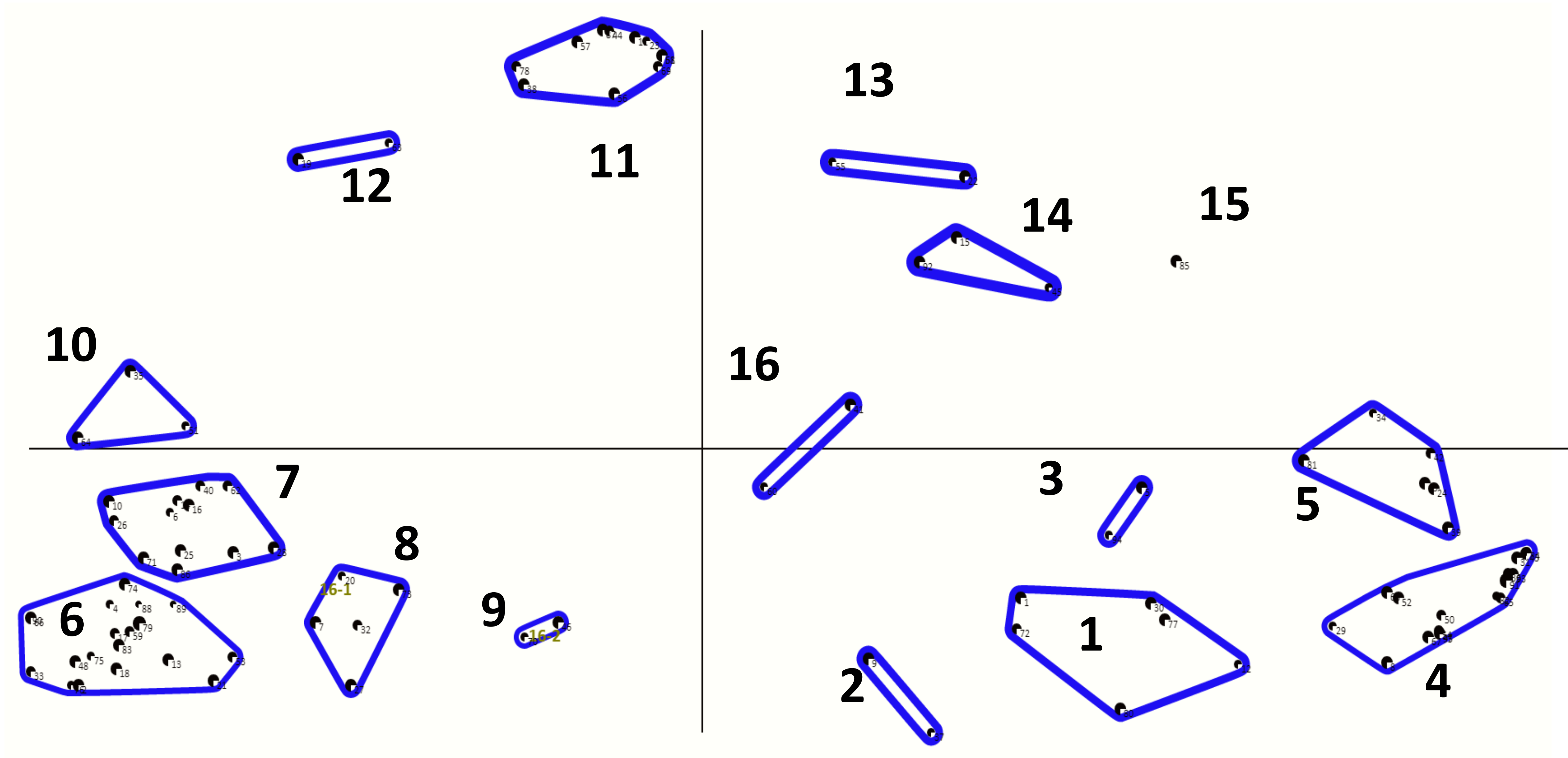

17-Cluster solution

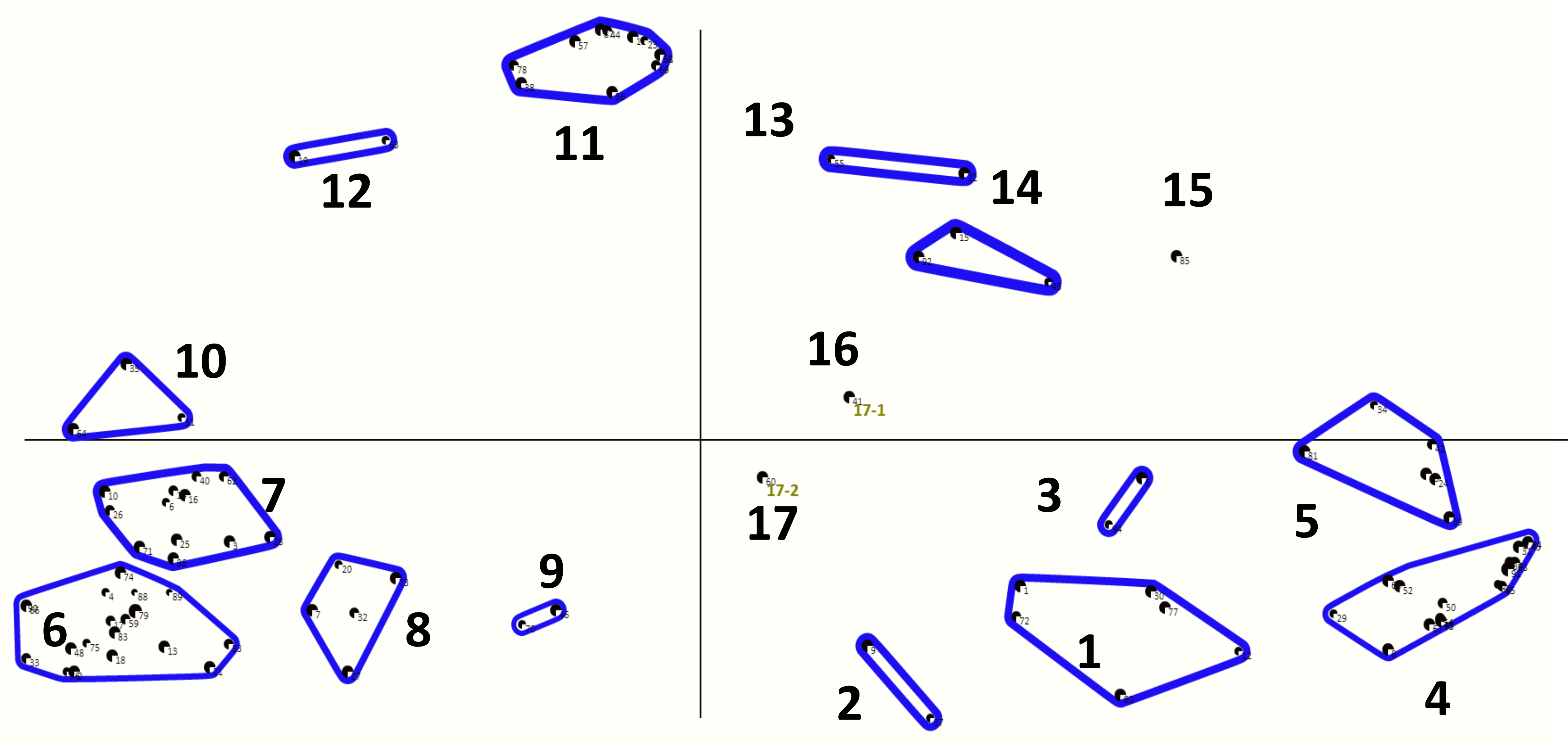

18-Cluster solution

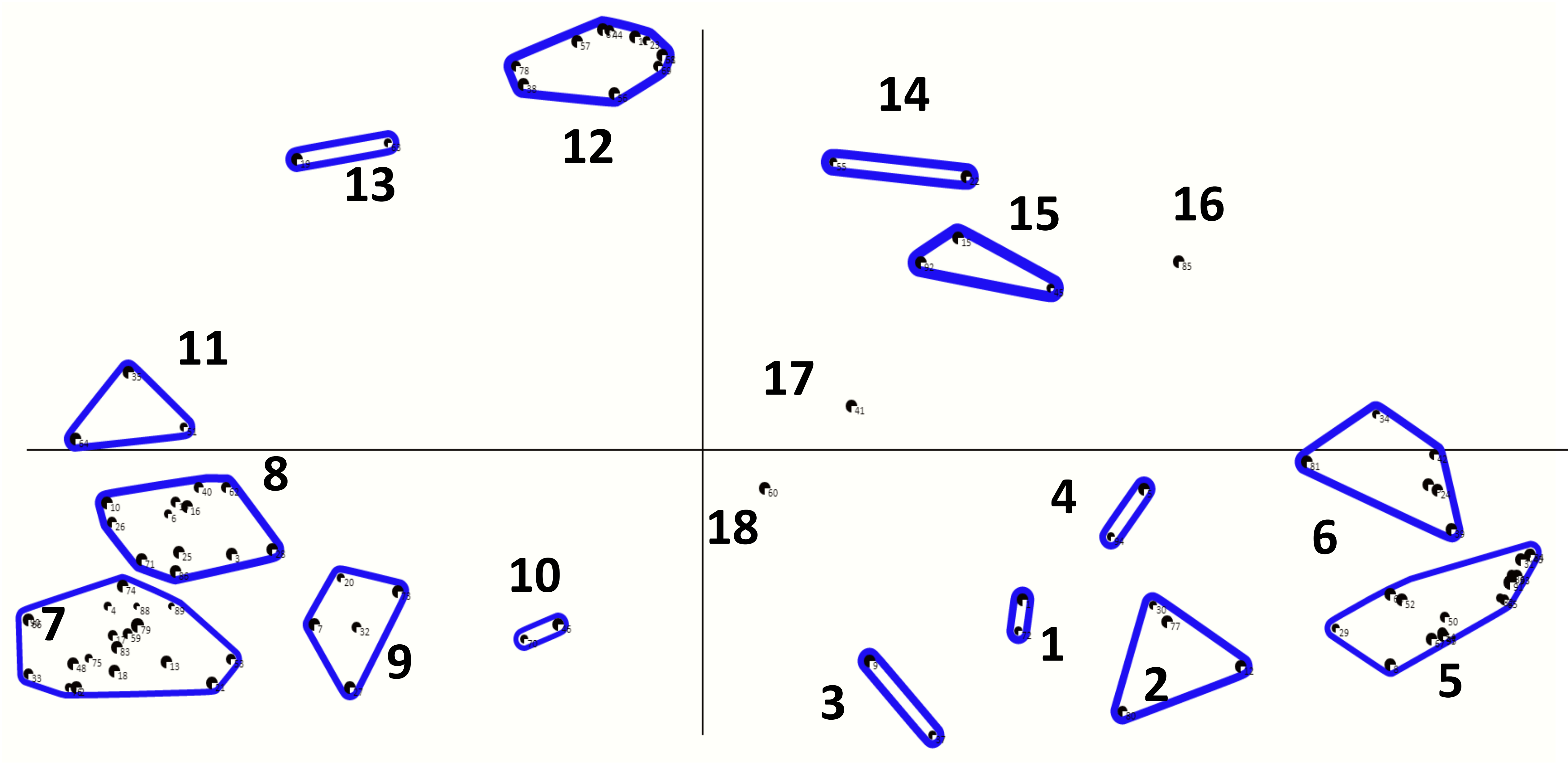

Supplement: Supplementary file 1 [file mps-08-00024-s001.zip › Supplementary document 9, Candidate concept maps with 95 statements.pdf]
