# Supplementary figures and images for: Development of a Reporting Guideline for Trochim’s Concept Mapping
Source: Methods Protoc. 2025 Mar 3;8(2):24. doi: 10.3390/mps8020024 (PMC11932253; doi:10.3390/mps8020024)

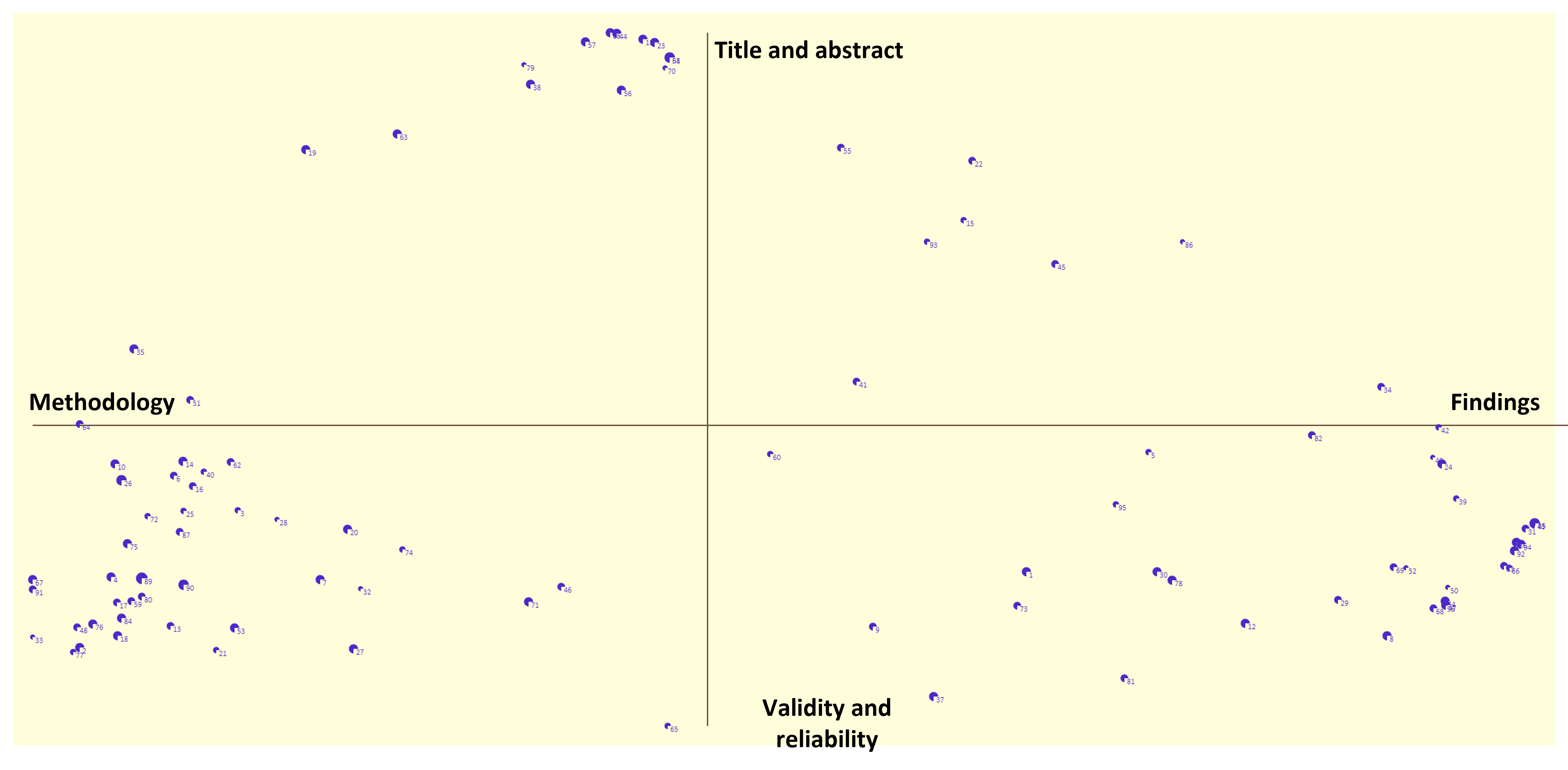

Supplement: Supplementary file 1 [file mps-08-00024-s001.zip › Supplementary document 7, Point map with 96 statements.png]
